# Psychology's Questionable Research Fundamentals (QRFs): Key problems in quantitative psychology and psychological measurement beyond Questionable Research Practices (QRPs)

**DOI:** 10.3389/fpsyg.2025.1553028

**Published:** 2025-08-25

**Authors:** Jana Uher, Jan Ketil Arnulf, Paul T. Barrett, Moritz Heene, Jörg-Henrik Heine, Jack Martin, Lucas B. Mazur, Marek McGann, Robert J. Mislevy, Craig Speelman, Aaro Toomela, Ron Weber

**Affiliations:** ^1^School of Human Sciences, University of Greenwich, London, United Kingdom; ^2^BI Norwegian Business School, Oslo, Norway; ^3^Norwegian Defence University College, Oslo, Norway; ^4^Advanced Projects R&D Ltd, Auckland, New Zealand; ^5^Ludwig-Maximilians-Universität München, Munich, Germany; ^6^University of the Bundeswehr Munich, Neubiberg, Germany; ^7^Department of Psychology, Simon Fraser University, Burnaby, BC, Canada; ^8^Jagiellonian University, Kraków, Poland; ^9^Sigmund Freud University Berlin, Berlin, Germany; ^10^Mary Immaculate College, Limerick, Ireland; ^11^University of Maryland, Baltimore, MD, United States; ^12^Edith Cowan University, Joondalup, WA, Australia; ^13^Tallinn University, Tallinn, Estonia; ^14^Faculty of Information Technology, Monash University, Melbourne, VIC, Australia; ^15^School of Business, The University of Queensland, Brisbane, QLD, Australia

**Keywords:** measurement, quantitative psychology, psychometrics, language models, ontology, epistemology, methodology, semantics

## Abstract

Psychology's crises (e.g., replicability, generalisability) are currently believed to derive from Questionable Research Practices (QRPs), thus scientific misconduct. Just improving the same practices, however, cannot tackle the root causes of psychology's problems—the Questionable Research Fundamentals (QRFs) of many of its theories, concepts, approaches and methods (e.g., psychometrics), which are grounded in their insufficiently elaborated underlying philosophies of science. Key problems of psychological measurement are critically explored from independent perspectives involving various fields of expertise and lines of research that are well established but still hardly known in mainstream psychology. This comprehensive multi-perspectival review presents diverse philosophies of science that are used in quantitative psychology and pinpoints four major areas of development. (1) Psychology must advance its general philosophy of science (esp. ontology, epistemology, methodology) and elaborate coherent paradigms. (2) Quantitative psychologists must elaborate the philosophy-of-science fundamentals of specific theories, approaches and methods that are appropriate for enabling quantitative research and for implementing genuine analogues of measurement in psychology, considering its study phenomena's peculiarities (e.g., higher-order complexity, non-ergodicity). (3) Psychologists must heed the epistemic necessity to logically distinguish between the study phenomena (e.g., participants' beliefs) and the means used for their exploration (e.g., descriptions of beliefs in items) to avoid confusing ontological with epistemological concepts—psychologists' cardinal error. This requires an increased awareness of the complexities of human language (e.g., inbuilt semantics) and of the intricacies that these entail for scientific inquiry. (4) Epistemically justified strategies for generalising findings across unique individuals must be established using case-by-case based (not sample-based) nomothetic approaches, implemented through individual-/person-oriented (not variable-oriented) analyses. This is crucial to avoid the mathematical-statistical errors that are inherent to quantitative psychologists' common sample-to-individual inferences (e.g., ergodic fallacy) as well as to enable causal analyses of possibly underlying structures and processes. Concluding, just minimising scientific misconduct, as currently believed, and exploiting language-based algorithms (NLP, LLMs) without considering the intricacies of human language will only perpetuate psychology's crises. Rethinking psychology as a science and advancing its philosophy-of-science theories as necessary fundamentals to integrate its fragmented empirical database and lines of research requires open, honest and self-critical debates that prioritise scientific integrity over expediency.

## Questionable Research Practices (QRPs): Surface-level symptoms obscuring fundamental problems still largely overlooked

Psychology's crises in replicability, validity and generalisability reflect a lack of scientific and societal confidence in its research findings (Newton and Baird, 2016; Open Science Collaboration, 2015; Schimmack, 2021; Yarkoni, 2022). Many psychologists attribute these crises to the improper application of established research methods—termed *Questionable Research Practices* (QRPs; John et al., 2012). These involve hypothesising after the results are known (HARKing), analysing data relentlessly to obtain statistically significant results that support the researchers' hypotheses (*p*-hacking), testing statistical associations of randomly combined variables without any theoretical hypotheses (fishing) and other questionable practices (Andrade, 2021; Earp and Trafimow, 2015). For the meticulous method expert, these flaws are readily identifiable, as are their remedies—larger samples, more robust statistics, more data transparency (open science, preregistration; e.g., Nosek et al., 2015; Zwaan et al., 2017). Thus, do psychology's crises arise just because psychologists are more prone to scientific misconduct than scholars in other disciplines?

### Psychology's Questionable Research Fundamentals (QRFs)

Most quantitative psychologists use approaches (e.g., research designs) and methods of empirical inquiry (e.g., rating ‘scales', statistical analyses) that are well-established in the field. Its leaders focus on advancing and applying these standards meticulously, wary of Questionable Research Practices (QRPs). We believe, however, that psychology's recurring crises cannot be overcome by just improving the same practices. We believe a fundamental rethinking is necessary.

Like all scientific activities, the approaches and methods of quantitative psychology are built on *presumptions*, which inform their rationales and operations—thus, on ideas that are taken for granted with confident belief until it can be proved otherwise. All theories, approaches and methods are also built on beliefs about what exists for us to know about, how we can generate knowledge and what is possible for us to know and in what ways. These *presuppositions*—fundamental, often unstated beliefs that underlie a system of knowledge—guide the decisions that any empirical scientist must make about what to study, what to regard as fact, what questions to address, what procedures and operations to use for exploring these as well as how to interpret results (Collingwood, 1940; Fleck, 1935/1979; Kuhn, 1962/1970; Uher, 2013; Valsiner, 2012; Weber, 1949). These fundamental beliefs may not be considered explicitly by everyone doing quantitative research. Still, as generalised views on how to do science, they influence all scientific activities in a field.

We have come together as scholars from different backgrounds and disciplines to critically reflect on quantitative psychology's research fundamentals and its current problems because a classical review, which always provides just a few authors' views, is insufficient. There are also no criteria on which a classical review could be based—because what is required is a rethinking of the very fundamentals on which many established practices are built. We therefore do not discuss ways to improve specific quantitative methods and approaches (e.g., statistical modelling) or their meticulous application, as commonly done. In our view, questionable research practices are just surface-level symptoms that distract from and obscure the root causes of psychology's crises—the *Questionable Research Fundamentals (QRFs)* of many of its theories, concepts, approaches and methods. Therefore, our focus is on making explicit and scrutinising the fundamental principles and rationales on which quantitative psychology is currently built. We outline alternative ones on which it could and should be built in the future.

This also requires critically analysing and elaborating the underlying *philosophies and theories of science*. Their relevance for quantitative psychology, however, is often overlooked. Many regard them as a mere specialist field, studied by just a small minority of psychologists. But all scientific research is based on a philosophy and theory of science—otherwise it would not be science. Specifically, all science is aimed at understanding the ‘world'—that is, it has a basic ontological orientation. All science is also concerned with our knowledge of this ‘world'—thus, it also has a basic epistemological orientation. Now, what does this involve?

#### Philosophy and theory of science—The fundamentals of scientific inquiry: Ontology, epistemology and methodology

Philosophy of science is concerned with the most fundamental questions of scientific inquiry. It involves ontology, epistemology, methodology and further branches of philosophy. *Ontology*, the philosophy and theory of being, is concerned with the most fundamental kinds of being that may be taken to exist, especially with their categorisation, structures and relations. *Epistemology*^1^, the philosophy and theory of knowing, is concerned with the nature and scope of knowledge that we can generate about specific kinds of being. This involves, amongst others, the justification of knowledge claims, concepts of ‘truth', logic and rationality. Epistemological presuppositions influence how researchers frame and design their research as well as how they view the relation between themselves and their objects of research—between the researcher and the researched. *Methodology*, the philosophy and theory of methods, in turn, connects abstract ontology and epistemology with empirical research. It provides justification for why specific procedures and operations (methods), but not others, are suited to explore specific objects of research and specific questions (Ali, 2023; Hartmann, 1964; Mertens, 2023; Poli and Seibt, 2010; Uher, 2022b, 2025; Valsiner, 2017).

In psychology and other sciences, many different ontologies, epistemologies and methodologies have been developed for different objects of research, different aims and purposes, and from different worldviews. This leads to pronounced differences in the specific ways of doing science that are pursued in a field—thus, to different paradigms.

#### Research paradigms in psychology: Diversity in the ways of doing science

A *paradigm* is a distinct framework that provides a coherent set of theories, models, concepts, terms, instruments and practices that are often considered conventional in a field and that build on a specific worldview and specific presuppositions and values. Paradigms may arise in a field from a single scholar's research that serves as an exemplar for solving fundamental problems (e.g., Newton's). Its successes promote consensus among other scholars and agreement on the framework on which it is based. Often, however, paradigms emerge gradually over time from theoretical, methodical and empirical advances that are made by many scholars in a field, each exploring specific problems and questions. Some paradigms are already more elaborated and coherent in their philosophical fundamentals, whereas others are more implicit and still awaiting coherent elaboration. Paradigm-specific jargon, however, often makes it difficult to immediately see commonalities and differences between paradigms. Their elaboration, however, is important to recognise the implications that paradigmatic differences have for empirical research. This is also necessary to understand the different quality criteria and standards of evaluation that apply to different paradigms and that often preclude direct comparisons (incommensurability; Bird, 2022; Kuhn, 1962/1970).

These complex fundamentals are worth exploring in their own rights (Ali, 2023; Fahrenberg, 2013, 2015; Holzkamp, 1983; Jovanović, 2022; Mertens, 2023; Toomela and Valisiner, 2010; Uher, 2018a, 2021c; Valsiner, 2017). But we do not aim to systematically elaborate them here and such is not necessary for our analyses. Like all scholars, we have our specialisations. Not all of us are scrutinising and elaborating the philosophy-of-science fundamentals of theories, concepts, terms, approaches and methods. Still, in this article, we want to create and increase awareness of the philosophical and theoretical dimensions underlying quantitative research in psychology and the disciplinary crises that it encounters. Therefore, we highlight important points to enable a more in-depth understanding of the current problems and their underlying Questionable Research Fundamentals (QRFs).

For this purpose, we aim to provide a more comprehensive overview of independent perspectives that can and should be taken on quantitative psychology's current status and development as a science. These involve many established lines of research from smaller communities of research and practice, often published outside of mainstream journals and thus, outside most psychologists' focus. As experts in our respective fields, we independently provide a critical reflection of what we see as quantitative psychology's main problems and what as the key tasks that must be tackled. We present solutions that have already been developed, explain their fundamentals and direct readers to key publications. This highlights another crucial point.

#### Diverse perspectives, philosophies and theories of science required in psychology

In any given discipline, there can be no single one-and-only right way of doing science—especially not in psychology, given that it explores phenomena as diverse as brain morphology, physiology, behaviour, experience, social interaction, language and other socio-cultural products of the human mind (Uher, 2021c). This highlights a further key point. Diverse perspectives, philosophies and theories of science are not just possible in psychology—they are even necessary, also in quantitative psychology. This requires, first and foremost, awareness and efforts to make basic presuppositions explicit and thus, accessible to elaboration and analysis. This also requires scholars to be tolerant and open to different perspectives to be able to not just pinpoint and critically discuss differences but also to identify communalities—because these may not always be obvious (Uher, 2024).

Indeed, although each of our independent contributions has its own focus and rationale, they also show systematic connections with one another, thereby creating a poly-perspectival and more comprehensive overview than any review by single authors could provide. With our compilation of different perspectives, ways of thinking and doing science, we also aim to foster the scientific spirit of an open debate in which we can make explicit our most basic philosophical presuppositions, challenge established concepts, theories and practices, advance novel ways of thinking and exchange controversially—yet constructively and collegially—about scientific psychology.

### Outline of this article

**Figure 1:**
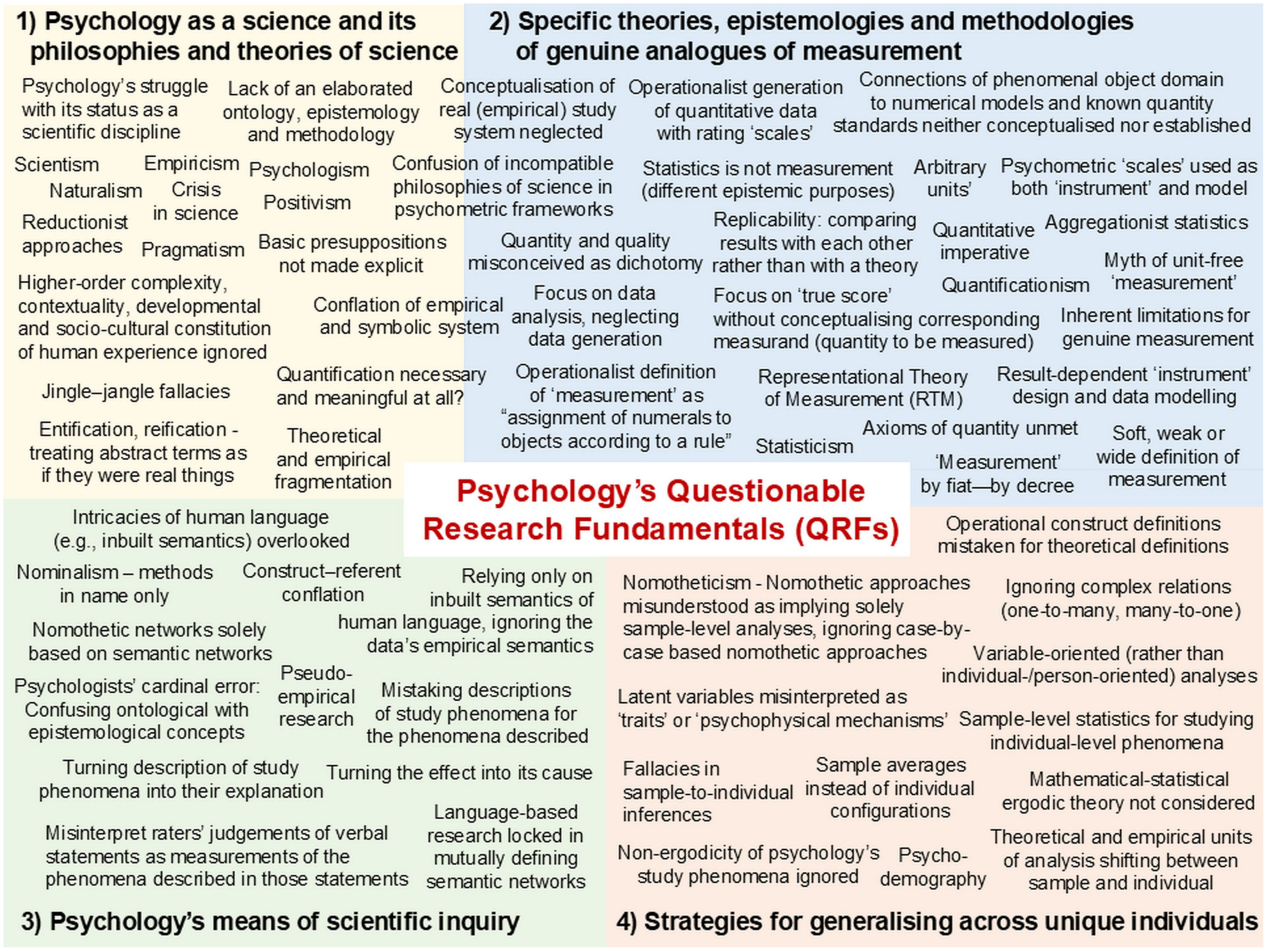
Psychology's Questionable Research Fundamentals (QRFs): Four main areas of development.

Our critical analyses are grouped into four main areas that cover different topics, problems and research questions and that, in our view, require remediation, elaboration and further development (Figure 1). Topic 1 starts by exploring quantitative psychology as a science. *Lucas Mazur* reflects on psychology's struggle with its scientific status and on the problems, promises and perils of scientism. *Aaro Toomela* elaborates on what science actually is as well as on the imperative to advance psychology's ontology, epistemology and methodology and to align them to one another to develop coherent paradigms. *Jack Martin* reminds us of the inherent contextuality of human experience that makes up personhood and draws conclusions for quantitative and experimental psychology.

Topic 2 is devoted to the specific epistemological, methodological and theoretical foundations of psychometrics and psychological ‘measurement', highlighting fundamental differences to physical measurement that are still not well considered. *Jana Uher* explores the conceptual problems entailed by psychology's operationalist definition of ‘measurement' and quantitative data generation with rating ‘scales', and highlights incompatibilities in the epistemological framework on which psychometrics is built. *Jörg-Henrik Heine* and *Moritz Heene* locate the failed promises of psychological ‘measurement' in the impossibility to establish one–to–one relations between the phenomenological object domain and the mathematical metric space of positive real numbers. *Paul Barrett* concurs that, without meeting the axioms of quantity and the human mind's peculiarities, quantitative psychology cannot implement genuine measurement processes. He highlights that the increasingly popular use of generative language algorithms cannot solve these fundamental problems. *Robert Mislevy* derives from the contextuality of human experience and learning a socio-cognitive approach that re-conceptualises the theoretical and philosophical framework that is necessary for making justified inferences from quantitative educational assessments in applied settings, while avoiding conceptual errors inherent in current conceptions. *Jana Uher* demonstrates that statistics and measurement are different scientific activities designed for different epistemic purposes. She specifies basic criteria and methodological principles and explains the system of modelling relations that are epistemically necessary for establishing genuine analogues of measurement in psychology.

Topic 3 explores the intricate relations between psychologists' study phenomena (e.g., participants' beliefs) and their means for investigating these phenomena (e.g., descriptions of beliefs in rating ‘scales' and models). *Jana Uher* highlights that their logical distinction (in each study) is an epistemic necessity to avoid conflating ontological with epistemological concepts—psychologists' cardinal error. *Jan Ketil Arnulf* therefore demands a more critical reflection on the role of human language in scientific inquiry. He demonstrates the epistemic necessity to distinguish between empirical and semantic research problems by showing that the inbuilt semantics of item statements, analysed through natural language algorithms, produces results similar to those obtained from empirical rating studies. *Ron Weber* analyses the ontology of construct–indicator and indicator–instrument relationships and introduces novel ontological concepts to analyse the applicability of constructs and their operationalisations (indicators) to different subsamples of populations, highlighting their implications for instrument development.

Topic 4 critically analyses psychology's approaches for generalising findings across unique individuals. It demonstrates that psychology's default use of sample-level statistics to explore individual-level phenomena ignores the mathematical-statistical foundations of such inferences (ergodic theory), the non-ergodicity of psychology's study phenomena as well as the peculiarities of complex living systems and therefore entails various inferential fallacies. *Craig Speelman* and *Marek McGann* highlight that the common sample-to-individual inferences build on the ergodic fallacy, thereby contributing to psychology's inferential and reproducibility problems, and they present pervasiveness analysis as an alternative approach. *Jana Uher* shows that, to avoid fallacies when making sample-to-individual inferences, psychology must advance case-by-case based (not group-based) nomothetic approaches, implemented through individual-/person-oriented (not variable-oriented) analyses. This is essential for identifying actual commonalities and differences among individuals as well as for enabling causal analyses to unravel (possibly) underlying structures and processes.

We close with general conclusions and future directions, highlighting that just minimising scientific misconduct and exploiting the new generative language algorithms to design ‘scales' and constructs, as increasingly done, will not remedy but only intensify psychology's problems and crises. Instead, tackling psychology's Questionable Research Fundamentals (QRFs) requires critical self-reflection and a fundamental rethinking of doing science in psychology.

To give new impetus to the current debates, we now discuss each of the four areas of development that we have identified (Topics 1 to 4) and present various independent perspectives, each focussed on specific problems and research questions. We analyse commonalities and differences of established and alternative ways of doing science in quantitative psychology, highlight their underlying philosophies of science, pinpoint key issues and provide novel insights.

## Topic 1: Quantitative psychology as a science: Key assumptions and the necessary philosophy-of-science fundamentals

Quantitative psychology has been developed in response to continued doubts, first voiced by Immanuel Kant in the 18th century, on psychology's ability to become an exact experimental—thus, a ‘real'—science. But even in the 21st century, psychology is still struggling with its status as a scientific discipline (Uher, 2021c).

### Why does psychology continue to struggle with its scientific status? The blinding promises and perils of scientism

In the attempt to make the field indubitably “scientific”, quantitative psychologists often end up embracing *scientism*, the belief that “only scientific knowledge counts as real knowledge” (Williams, 2015, p. 6). Why do many continue to believe in the promises of scientism, while ignoring the problems and even perils that it brings? In his line of research on psychology's history and philosophy of science, Lucas Mazur explored this question conceptually (Mazur, 2015, 2017, 2021, 2024a; Mazur and Watzlawik, 2016). In his empirical research, he encourages interpretive, anti-naturalistic (treating psychological phenomena not like natural facts), dynamic and contextualised approaches—even when making use of quantitative methods (Mazur et al., 2022; Mazur and Sticksel, 2021; Mazur, 2022, 2024b,c).

#### The problems

In psychology, there is a persistent blindness to the problems of scientism. These include *quantificationism* (viewing quantitative information as generally superior to qualitative information), *naturalism* (viewing research data as raw, objective ‘natural' facts in need of little or no interpretation), *statisticism* (viewing statistics as a complete or sufficient basis for scientific methodology) and *psychologism* (reducing thought and knowledge to internal psychological characteristics of individual minds), amongst others (Lamiell, 2018, 2019a; Sugarman, 2017; Uher, 2022b). From time to time, the problems become so undeniable as to demand a response (e.g., the replication crisis). But, at those moments, many quantitative psychologists perennially respond with *more of the same* (e.g., open science, robust statistics)—in effect, “kicking the can down the road” (Steinmetz, 2005; Tolman, 1992). As Valsiner and Brinkmann (2016) suggested,

“[it] cannot be the case that this unfortunate situation occurs only due to the intellectual transformations within the history of psychology itself. There must be some societal catalytic process for the meta-theoretical blindness in the field” (p. 87).

For the fact that many researchers do not look the problems of scientism squarely in the face, there is, amongst many others (Uher, 2022b), a two-sided societal reason. This is the blinding power of scientism, particularly the belief that quantification is a step towards prediction and ultimately control (Hacking, 1990; Porter, 1995). On the one side, many researchers are so thoroughly pulled towards scientism that they do not reflect on this choice of direction. Indeed, they do not even see it as a choice but as the only way to go—rendering the matter “too obvious” to warrant consideration and its potential loss as their lodestar too disorienting. On the other side, if they paused for serious reflection, they would see a vision that forces them to close their eyes in disgust, or even horror, which is likewise deterring proper reflection. This tension creates a form of collective avoidance that perpetuates problematic meta-theoretical and methodological assumptions (Mazur, 2021).

#### The promises

The gravitational forces of quantification and mechanistic causality have become so powerful that they distort many researchers' very perception of ‘reality', as reflected in the belief that “science is the only path to understanding” (Gnatt, 2018). This view has become deeply entrenched in quantitative psychology, where it is widely believed that human experience and behaviour can be reduced to measurable, predictable units (Michell, 2022). The contemporary emphasis on optimisation—both in academia and society at large—exemplifies this mindset. It is often reinforced, paradoxically, even by attempts to resist this trend: calls to “unplug” or “slow down” frequently come packaged in the language of optimisation as quantifiable steps leading to quantifiable benefits.

Over a century ago, social theorists presciently identified that this shift towards quantification was part of the broader process whereby scientists become tools of their own tools (Danziger, 1990; Daston and Galison, 2007; Poovey, 1998; Valsiner, 2007, 2012). Max Weber (1904–05/1992) noted how commitment to non-calculable goals was increasingly viewed as irrational. Durkheim (1893/1984) recognised the cultural dominance of this new rationality but struggled to envision alternatives. Simmel (1900/1978, p. 443) similarly observed “the growing preponderance of the category of quantity over that of quality, or more precisely the tendency to dissolve quality into quantity”. Today, this tendency has become further intensified under the auspices of neoliberal consumerism, which further privileges quantity over quality (Sugarman, 2017). Therefore, many researchers keep their eyes fixed on the horizon of ‘progress' that this quantity-focused worldview promises, driven simultaneously by an unspoken anxiety that any deviation from it might halt humanity's collective march forward.

#### The perils

The disturbing implications of scientism become apparent when we examine its logical conclusions. As Maslow (1966, p. 75) noted, in scientism, “the blueprints are more real than the houses. The maps are more real than the territory”. For many psychologists, this is already a disturbing denial of our humanity. However, the prioritisation of measurement, prediction and control at the cost of all that does not fit the mould points in even darker directions. This became apparent, for example, in *classical positivism*, which is built on the presupposition that the social ‘world' can be explored just like the natural ‘world' through observation, experimentation and measurement by independent researchers who work objectively and separated from their own values. The founders of classical positivism, Henri de Saint Simon and Auguste Comte, even called people resistant to positivism “parasites” and mere “dung-producers”, arguing that they “transmit to their successors no equivalent for what they received from their predecessors” and therefore “should be treated like cattle” (de Lubac, 1995).

This dehumanising language and logic haunt the boundaries of both science and morality—of what *could* and what *should* be done. It is no coincidence that such thinking appears in dystopian works like Animal Farm, Brave New World as well as Frankenstein (“the modern Prometheus”). It has also found its expression in eugenics, communism and National Socialism. Meanwhile, embracing scientific approaches to understanding human nature has become second nature to many researchers. However, when this embrace becomes exclusive and dismissive of other perspectives, researchers risk creating the very scenarios that science fiction—and history—have long warned against. The tendency to quickly pass over figures like Comte and Saint-Simon in psychological teaching and textbooks may perhaps reflect not just the *naturalisation* of social science—its treatment like a natural science—but also an unconscious recoil from its more troubling implications.

#### The prospects

To be swept away by a scientistic vision of humanity is to soar on the wings of Icarus. Once in the air, many either keep their eyes focused on the blinding sun towards which they are heading (the promises), or they keep them closed in terror before the fall (the perils). Either way, they do not want to *see*. Below one can hear the flapping of the perilously glued-on wings:

“If I could only discover some external indicator of, for example, happiness or anxiety, some litmus paper test of the subjective, I would be a very happy man. But happiness and anxiety now exist in the absence of such objective tests. It is the denial of this existence that I consider so silly that I won't bother arguing about it. Anyone who tells me that my emotions and my desires don't exist is in effect, telling me that I don't exist” (Maslow, 1966, p. 47).

Beneath the dismissal of this “silly” suggestion, one can hear unease, even dread, but also desire—the simultaneous allure of, and repulsion at, scientistic thinking. By contrast, social psychologist Gustaw Ichheiser wrote:

“[S]ocial scientists should, in my opinion, not aspire to be as ‘scientific' and ‘exact' as physicists or mathematicians, but should cheerfully accept the fact that what they are doing belongs to the twilight zone between science and literature.” (Ichheiser in a Letter in 1967, cited in Rudmin et al., 1987, p. 171).

This perspective suggests a practical path forward: integrating insights from the humanities more explicitly and thoroughly into psychological inquiry (Aeschliman, 1998; Bruner, 1990; Freeman, 2024; Mazur, 2015, 2017, 2021, 2024a; Sugarman and Martin, 2020). This is not a rejection of science. It is a recognition that—even after scientific methods have been applied in their proper scope to a limited range of phenomena in psychology (Mazur and Watzlawik, 2016; Taylor, 1985)—there remains much to study from other points of view and via other methods of investigation. While echoing the warnings against unreflective quantification, including against the faulty assumption that psychometrics could enable genuine measurement (Uher, 2021a), this is not a rejection of the thoughtful use of numbers as meaningful depictions of psychological phenomena (Mazur, 2022, 2024b). Indeed, both quantitative and qualitative methods can be both useful and problematic (Bevir and Blakely, 2018; Holzkamp, 2013). Psychologists do not even have to stop trying to positively impact the social ‘world' around—after all, most of what is thought of as “psychological” already involves active engagement with that social ‘world' (Ichheiser, 1943; Smedslund, 2016; Wittgenstein, 1953). This, however, is a reminder of how the temptations of power and control—which in psychology take the form of scientism—can blind many psychologists to the perpetual challenge of human hubris.

“Let me warn you, Icarus, to take the middle way, in case the moisture weighs down your wings, if you fly too low, or if you go too high, the sun scorches them. Travel between the extremes.” (Daedalus to his son Icarus)

The humanities, such as history, philosophy and literature but also rhetoric, music, the performing and visual arts, religious studies and theology, can help psychology to break free from the chains of scientism—from the desire for, and fear of, what researchers (mis)take to be scientific control. A more open-minded interweaving of fields will allow psychology to more richly understand, appreciate and wonder at the human condition.

This can and should entail systematic elaborations also of psychology's philosophy and theory of science. More and more psychologists are exploring epistemological and methodological issues as well as ontological questions, each with their specific focus on specific research questions and from their specific perspectives. At some point, the different elements of scientific inquiry used in a line of research should be elaborated and coherently aligned with one another and with the specific presumptions, beliefs and values on which they are based. This means that the specific epistemological approach used in a line of research should correspond to the specific ontological presumptions made and both should inform the corresponding methodology to guide the development of suitable methods (Al-Ababneh, 2020; Ali, 2023; Mertens, 2023). An example of such a coherently elaborated philosophy of science is the structural-systemic paradigm. This paradigm also opens a more fundamental perspective on psychology's crises, which goes much beyond the currently discussed surface-level symptoms of problems in replicability, validity and generalisability.

### The crisis still overlooked: Psychology's ontology, epistemology and methodology must be grounded in a structural-systemic paradigm

In his line of research on the ontological, epistemological and methodological foundations of psychology, Aaro Toomela highlighted that psychology's crisis is much more profound than currently considered. In fact, it is a c*risis in science*—defined as a situation where there is no generally accepted system of science (Vygotsky, 1982, p. 373, Vygotsky, 1997). Indeed, psychology is divided into mainstream psychology, which is pursued by the majority, and non-mainstream psychology, which challenges ontological, epistemological and methodological principles that are generally accepted by the mainstream (Toomela, 2014a, 2019).

Any science prospers best through collective efforts—through working as a global team. Scientific progress through collaboration is hindered, however, when it requires the discovery of novel questions that entail entirely novel perspectives on the object of research (Toomela, 2007b). This process is stretched over time. First, novel questions must be discovered and justified by individual scholars. When the questions are important, they must form groups of like-minded scholars who take the questions seriously and start developing new research approaches. Thereafter, it may still take considerable time before the importance of these novel questions and the novel approaches for answering them will be recognised by mainstream scholars.

Where is psychology now? There already is a set of novel questions about and novel perspectives on the general scientific worldview of mainstream psychology. These novel questions, as well as convincing approaches for tackling them, are increasingly discussed by various groups of non-mainstream scholars. But they are still largely ignored by mainstream psychologists. These questions concern the most basic principles of science—its ontology, epistemology and methodology. But first, what is science?

#### What is science? And what is scientific understanding?

First, it is important to acknowledge that science is not necessary for achieving knowledge. Moreover, all knowledge about the ‘world' is acquired only from information obtained directly through the sensory organs (in humans and animals alike). Most of the ‘world', however, is not directly accessible with our human-specific senses. To understand the essence of science, it is necessary to distinguish between these two aspects of the material ‘world'. Science came into being when humanity began to study those parts of the ‘world' that are not accessible through our senses: science aims at understanding the ‘world' that is not sensorily accessible in order to explain the ‘world' that we can perceive with our senses (Toomela, 2022). Importantly, things and phenomena that appear to be identical in our senses can sometimes be different in some aspects that we cannot sensorily perceive. Vice versa, things or phenomena that differ in our sensory perception may sometimes have common characteristics that are imperceptible to us. The essence of scientific methods is to help us discover such aspects of the ‘world' that may causally underlie the directly perceivable and which may thus help us to explain the ‘world' as it appears to us. Research methods that do not allow us to describe the parts of the ‘world' that are inaccessible to our human senses therefore do not help us to advance our scientific knowledge.

#### Scientific understanding of the human psyche requires a unifying ontological theory

Almost a century ago already, Vygotsky provided convincing arguments that psychology cannot become a true science without a *general-unifying theory* (Vygotsky, 1982; also Toomela, 2007c, 2014b, 2017)—an ontological theory of what the psyche is. The psyche as a whole can be defined as “a specifically organised form of living matter. Its purposeful behaviour in anticipating environmental changes that are harmful [or beneficial] for itself as a whole is based on individual experience” (Toomela, 2020, p. 29). This whole can be distinguished into parts at different levels of analysis. At the most general level, the psyche can be distinguished into the psychical individual and that part of the environment to which it relates (called the psychical environment; Toomela, 2020, also Koffka, 1935). In the psychical individual, further interrelated parts can be distinguished. Luria (1973) showed that the true material parts of the psyche are the different brain regions each with their unique function. *Vygotsky's theory of higher psychical functions* explains how the human psyche emerges when cultural signs become part of the structure of an individual's psychical system, which underlies its psychical processes (Toomela, 2016b; Vygotsky, 1994). Hence, within this general ontological theory, the psyche is defined as a structural system—as a whole. Such a theory is crucial to understand the essence of the human nature.

*Structural-systemic ontologies*—that is, presuppositions that the material ‘world' is composed of hierarchies of interrelated parts that form qualitatively distinguishable whole structures at certain levels of analysis—are used in other sciences as well. Chemistry, for example, conceptualises molecules with different qualities, atoms as parts of molecules as well as the molecules' structure and their composition of atoms. Some molecules, called isomers, are composed of identical sets of atoms, but these are arranged in different relations from which qualitatively different molecules emerge as structured wholes. When we ontologically assume that the ‘world' is systemically organised in interrelated structures in which parts are forming qualitatively different wholes, then an epistemology must be defined that corresponds to that ontology. Accordingly, the aim of science is to construct structural-systemic knowledge about that ‘world'.

#### Psychology requires a structural-systemic epistemology

Mainstream (quantitative) psychology pursues knowledge about generalised patterns in large data sets and (mostly) linear cause-effect relationships. But with these approaches, there is no way to discover the parts and processes of the psyche as well as the specific kinds of relationships between them and from which the particular properties of the psyche as a whole emerge (Toomela, 2020). What is required is a more powerful epistemology that Toomela called *structural-systemic* (Toomela, 2003, 2009a, 2012, 2014d, 2015, 2016a, 2019). This epistemology was pursued by several scholars in the history of psychology (e.g., Luria, 1973; Vygotsky, 1994; Werner, 1948; Wundt, 1897). Many further theories with various concepts of “system” and “structure” were developed in different sciences (see Ramage and Shipp, 2020). Hence, there is not just one but many structuralist or systems epistemologies. Therefore, it is necessary to define what specific theory is followed in a given line of research.

In Toomela's *structural-systemic epistemology*, science is aimed at constructing knowledge about the part–whole structures of the things or phenomena studied. In this approach, scientific understanding provides answers to three main questions: What is the studied whole? What are the parts of the whole? And in which relationships are these parts? The origin of this epistemology can be traced back to Aristotle who suggested that knowledge is about causal structures of the ‘world'. He distinguished four *complementary kinds of causes*, nowadays called *material* (what are the parts), *formal* (what is the whole), *efficient* (what makes a change happen) and *final* (why does a change happen).

Today's mainstream psychology, by contrast, relies on a simplified Cartesian-Humean understanding of causality where only efficient causality is believed to be knowable. The Aristotelian perspective, however, shows that, to understand causality, all causes must be known. Specifically, the parts of a whole—its *material cause*—underlie what the whole is. Therefore, the whole cannot be understood without knowing the material cause because changes in the parts inevitably lead to the changes of the whole that is composed of and emerges from the parts. The whole, in turn, is the *formal cause*, which determines what external events can affect a system in principle. The processes that can change a whole, in turn, are the *efficient causes*. But they can cause changes only if that whole can potentially be changed by the given efficient cause. That is, what is being affected determines what can affect it and how it can be affected in principle. Consequently, efficient causality cannot be understood unless material and formal causes are understood at the same time as well. Final cause is as important as the other causes. It determines what can be the result of the change of the whole (for a thorough analysis of different theories of causality, see Toomela, 2019).

#### Methods do not yet make methodology

Structural-systemic approaches also require that psychology develops a theory and philosophy of its scientific methods—a general *methodology* (for outlines, see Toomela, 2022). Mainstream psychology generally lacks an elaborated methodology. The common recipe-style books compiling ready-to-use methods, as used in quantitative psychology, do not yet make a methodology. Methodology, as the science of methods, explains how selected methods allow us to answer specific research questions. Each new question may require novel, methodologically grounded methods. But many quantitative methods used in psychology (e.g., statistical tests) are not grounded in an elaborated methodology. They provide only probability statements but no theoretical justification about how these methods could allow us to address specific research questions and to explore specific study phenomena (Toomela, 2011, 2014b, 2022; Toomela and Valisiner, 2010; Uher, 2025; Valsiner, 2017). Importantly, such methods do not enable us to develop a structural-systemic understanding of psychical phenomena. Why?

Quantitative psychology largely studies only *observable* behavioural performances (e.g., test results, responses to questionnaires) while aiming to explore the *non-observable* psychical processes enabling them (e.g., intellectual abilities). However, *observably identical* behaviours may emerge from interactions of *different underlying* psychical processes (Richters, 2021; Sato et al., 2009; Toomela, 2007a, 2008b, 2009b; Uher, 2022b). But when observations are encoded into variables, such that *observably identical* behaviours are taken to arise from *psychically identical* processes, then the most important information is already lost because there is no way to discover what different processes may underlie observably identical behaviours (Toomela, 2008b; also Danziger and Dzinas, 1997; Maraun and Halpin, 2008; Uher, 2021a). For example, individuals can generate correct answers to simple arithmetical tasks by mentally calculating, counting their fingers or just recalling memorised answers. But which of these processes they have actually used remains unknown when only their responses are encoded. Psychological research that ignores this crucial point is, in fact, a version of behaviourism and thus, unable to explore psychical phenomena (Toomela, 2000, 2008a,b,c, 2011, 2014c, 2019).

The structural-systemic conceptualisation of the psyche as a complex system also highlights that, as structural wholes, psychical phenomena cannot be explored by reducing them to parts and studying these in isolation. Such *reductionist approaches* are commonly pursued in quantitative psychology, however, where wholes, described in constructs (e.g., ‘intelligence'), are (conceptually) dissected into parts (e.g., verbal, numerical, spatial or reasoning abilities). Results obtained on these (conceptually) separated parts (e.g., different tasks in ‘intelligence tests') are then simply combined (e.g., averaged), assuming the index score could be a ‘measure' of the whole. Functional performances in higher cognitive abilities, however, are impossible without the involvement of various further processes (e.g., perception, reading comprehension ability, long-term memory). These must be present as well for complex cognitive processes to emerge at all. An individual's low performance in specific tasks therefore does not mean that the specific cognitive processes at which these tasks are targeted were not involved. Rather, it indicates only the individual's reduced or failed ability to use these processes in the given task situation (e.g., social pressure, noise). That is, complex cognitive processes can emerge only in the context of countless other concurrent processes and phenomena both internal (and thus, likewise hidden) and external to the individual (e.g., psychical, physiological, situational). This makes it impossible to determine the specific contribution that selected cognitive processes may make to observable task performances (Toomela, 2008b; Uher, 2022a, 2025).

These fundamental relations are elaborated also in another non-reductive ontology that focusses holistically on individual persons in the social, cultural and societal contexts of their lives. This *person-based ontology* (Martin, 2022) conceptualises human individuals as persons who are, at once, bio-physical and socio-cultural beings. Its origin can be traced back to Aristotle who conceptualised the human being as a bio-physical entity that develops within societies as a social and political being, thereby acquiring intellectual abilities (e.g., reasoning) and character (e.g., virtues). These *non-dualistic ontologies* differ profoundly from *Descartes' dualistic ontology* in which persons' material bodies are separated from their immaterial ‘minds', which raises the fundamental problem of how these might interact, such as to enable action (body–mind problem). Descartes' dualistic ontology dominated Anglo-American philosophy and psychology, which also pursued reductionist approaches, in which persons are reduced to their bio-physical, behavioural and psychical parts, while their complex life contexts are reduced to quasi-laboratory settings and psychometric testing conditions (Martin, 2022). Conceptualising persons as ontological units, by contrast, allows for considering the inherent contextuality of psychical phenomena as well as for pinpointing the implications that this has for quantitative investigations.

### The contextual constitution of psychological phenomena does not yield to methods of quantitative measurement and laboratory experimentation

In his line of research on the psychology of personhood, Jack Martin has highlighted the idea that psychological phenomena are constituted by human interactivity within the life contexts of human beings (Martin, 2013, 2024; Martin and Bickhard, 2013; Sugarman and Martin, 2020). What interests us most in our everyday lives is neither accessible through nor reducible to bio-physical phenomena, which are amenable to precise quantitative measurement. Phenomena, such as identity, self-other understanding, perspective-taking, imagination, purpose, creativity or existential concern, are socio-culturally, historically and biographically constituted (Kirschner and Martin, 2010). The contextual constitution of psychological phenomena cannot be illuminated by methods of quantitative measurement and laboratory experimentation that have proven so successful in natural, bio-physical science.

#### The socio-cultural life contexts of people

Our historically established socio-cultural communities are replete with practices, customs, traditions and ways of interacting, communicating and living. Our embeddedness and participation—from birth to death—within these contexts constitutes us as persons with self-other understanding, practical know-how, personal and collective identity, biographical storylines as well as moral and rational agency (Martin and Sugarman, 1999; Martin et al., 2003, 2010). These contexts, and our interactivity within them, make up our *personhood* in ways that do not lend themselves to experimental variation in laboratory study or to standardised, quantitative measurement. Our personal and collective being and living initiate us into the possibilities and constraints afforded by our socio-cultural contexts (Danziger, 1990; Martin and Sugarman, 1999; Valsiner, 1998). Yet, in the course of our lives, we are able to develop ways of acting and interacting that alter these contexts. We humans are caught up in a circle of existence within which generations of us inherit, transmit and modify our life contexts during our own lifetimes.

#### Problems of quantitative measurement and laboratory experimentation in psychology

Physical measurements in daily life and in science rely on *standard units of measurement*. Psychological measures, by contrast, rely primarily on *ratings* and *counts*. We have no objective, standard units with which to measure thoughts, ideas, opinions, emotions, actions, intentions, meanings or experiences—let alone to capture the more macro-level phenomena of human life, such as moral and existential concern that arise within the circles of existence that we inherit, adapt and pass on. Ratings of degrees of confidence, strengths of beliefs or levels of self-determination rely on the subjective judgements of researchers and research participants (Martin and McLellan, 2013; Uher, 2018a, 2022b, 2023a). Counting kinds of thought, frequencies of emotional occurrences or particular imaginings is unlike counting numbers of birds, heartbeats or users of public transit. Measuring physical states or processes is not akin to interpreting psychological states or processes (Lamiell, 2019b; Martin and Sugarman, 2009; Martin et al., 2015; Smedslund, 2021).

Unlike the trigonometry and calculus that can be applied to physics, psychology's statistical procedures do not enable precise point predictions and replications. Laboratory contexts in physics are specially constructed spaces for the careful observation and measurement of isolated phenomena under controlled conditions. Laboratory contexts in psychology, by contrast, mostly reduce and distort the everyday phenomena that they purport to study and ‘measure'. The phenomena studied in psychological laboratories are literally and figuratively “out of context”. In consequence, there is a large gap between the empirical findings of experimental psychology and the lives that we lead as historically situated, socio-cultural and biographical beings (Danziger, 1985a, 1990; Gergen, 2001; Martin, 2022, 2024; Valsiner, 2014a).

#### Psychology as a socio-cultural practice and its impact on society

Psychological science is itself a multifaceted set of historically established, socio-cultural practices that affect us in somewhat predictable but sometimes also highly unpredictable ways (Martin, 2024; Valsiner, 2012). More than any other social science, psychology claims to foster factual and progressive understanding of our existence, actions and experiences. Such claims, however, require a better footing than that provided by much of the current experimental and professional psychology. Specifically, they require a reimagining that goes well beyond what some regard as a crisis of replicability in psychological research findings.

Since at least the mid-1980s, a growing number of social scientists and other scholars have become less interested in psychology and psychotherapy as purportedly applied sciences. They argue that, by trying to align psychology's scientific aspirations and status to those of physics and by focussing just on the efficacy of its professional practices, we risk missing out on the larger and arguably more important impact that psychological and psychotherapeutic ideas and practices can have on contemporary cultures, societies and individuals (Martin and McLellan, 2013; Madsen, 2014). Scholarly inquiry that examines connections between the lives, works and sociocultural impact of psychologists can provide valuable information about how psychologists and psychology affect people and their life contexts and experiences (Martin, 2017; also Fleck, 1935/1979).

#### Alternative methods of psychological inquiry lead to new knowledge

Methods of life study, interpretation and writing, such as historical ontology (Hacking, 2002), biography and psychobiography (Kirschenbaum, 2007), ethnography (Rogoff, 2011), narrative inquiry (Hammack and Josselson, 2021), positioning theory (Harré and Van Langenhove, 1999) and life positioning analysis (Martin, 2013, 2024) aim to reveal dynamic reciprocities and relationalities that exist among people and their life ‘worlds'. Such research can suggest possibilities for balancing conflicting demands for change and stability that attend the ongoing, mutual co-constitution of ourselves and our societies within contemporary life. In view of the ongoing social conflicts, complex real-world problems and the many crises in our societies, democracies and global relationships, such approaches have become more important than ever. A psychology of persons and of their lives must attend directly to their life concerns as these are experienced and lived—rather than as simulated and probed in comparatively decontextualised experimental settings with equally decontextualised pseudo-‘measures'. Only by focusing on the actual lives and life conditions of real people can we, as psychologists, recognise and face directly the possibilities that we create for both humanity's flourishing and its peril with the aim of enriching the former and guarding against the latter (Martin, 2022, 2024).

This person-based ontology aligns with many of the ontological and epistemological commitments of critical realism. Different variants of realism and other philosophical theories have been developed in the sciences, many of which are also used in quantitative psychology. We discuss some of these now in our next Topic 2 with regard to the philosophy-of-science fundamentals underlying theories, methods and practices of psychological ‘measurement'.

## Topic 2: Fundamentals of psychological ‘measurement' and quantitative psychology—Crucial differences to genuine measurement

Physical measurement procedures are clearly not applicable in psychology given the peculiarities of its objects of research, such as their contextuality, developmental and socio-cultural constitution and inherent structural-systemic complexity (see Topic 1). Quantitative psychologists therefore developed their own definitions, concepts, theories and methods of ‘measurement' (therefore here put in inverted commas) largely independently from those of measurement established in physical science and metrology (the science of physical measurement and its application; Berglund, 2012; Mari et al., 2021; McGrane, 2015; Uher, 2020a). Still, quantitative psychologists often draw analogies to physical measurement and interpret their findings as ‘measurement' results that provide quantitative information about the phenomena studied in individuals. This entails conceptual errors because many psychologists are unaware of crucial differences in the underlying philosophies of science—and therefore also of contradictions that their conflation entails. Here we do not aim to provide a comprehensive comparison (see Uher, 2020a, 2021a,b, 2025). But we discuss key problems and important differences that are still largely overlooked. We exemplify these by specific theories and practices of psychological ‘measurement'.

### Psychology's operationalist definition of ‘measurement' and quantitative data generation with rating ‘scales'

In her transdisciplinary line of research, Jana Uher explored theories, concepts and approaches of measurement and quantification across different empirical sciences. *Transdisciplinarity* gained recognition as a new way of thinking about and engaging in scientific inquiry since the 1970s. Unlike cross-, multi- and inter-disciplinarity, it is aimed at exploring complex systems and complex (“wicked”) real-world problems that require the expertise of many scientific disciplines. Collaboration and integration across the sciences, however, are often hindered by discipline-specific jargon, theories, methods and practices. Transdisciplinarity^2^ is therefore aimed at exposing disciplinary boundaries and the fundamental, often unstated beliefs on which scientific systems are built (presuppositions). Making these explicit is necessary to understand the non-obvious differences in discipline-specific processes of scientific inquiry—especially in their underlying ontologies, epistemologies and methodologies—as well as in their resulting bodies of knowledge. This also allows for discovering hidden connections between different disciplines as well as for generating unitary intellectual frameworks that rely on but also integrate and transcend different disciplinary paradigms (Bernstein, 2015; Gibbs and Beavis, 2020; Montuori, 2008; Nicolescu, 2008; Piaget, 1972; Uher, 2018a,b,c, 2021c, 2024, 2025).

Using transdisciplinary approaches, Uher analysed epistemological and methodological fundamentals of theories, methods and practices of measurement and quantification established in psychology, social sciences, behavioural biology, physics and metrology. Her analyses pinpointed commonalities and differences, especially between psychological ‘measurement' (e.g., psychometrics) and physical measurement that are still hardly considered in pertinent debates (e.g., Uher, 2018a, 2019, 2020a, 2022a,b, 2025).

#### What actually is quantity?

The most basic concept for quantitative sciences is that of *quantity*. Surprisingly, however, most scholars seem to rely on their intuitive understanding of quantity rather than a scientific definition. This entails confusion as to what measurement actually is, especially when mere categorisation is misleadingly termed ‘nominal measurement' in psychology (Stevens, 1946) but also in engineering and metrology (Finkelstein, 2003; White, 2011). Some contend that measurement is solely defined through its process structure rather than also through a feature of its results (Mari et al., 2013). However, an elaborated process structure coordinating observations of the objects of research with our concepts, theories and models about them is basic to any form of elaborated scientific inquiry (Uher, 2025).

Ontological philosophy provides clear definitions. *Qualities* are properties that differ in kind (Latin *qualis* for “of what sort”). Length, weight, temporal duration and sound intensity are qualitatively different. *Quantities* (from Latin *quantus* for “how much, how many”), in turn, are divisible properties of entities of the same kind—thus, of the *same quality* (Hartmann, 1964). When qualitatively homogeneous entities change in quantity, such as by adding or dividing them, their meaning as entities of that specific quality remains unchanged. Placing several boxes side-by-side (concatenation) changes the quantity of their joint width but does not alter its quality as being that of length. That is, entities of equal (homogeneous) quality can be compared with one another in their divisible—quantitative—properties in terms of their order, distance, ratio and further relations as specified in the *axioms of quantity* (e.g., equality, ordering, additivity; Hölder, 1901,^3^; Barrett, 2003; Michell, 1990; Uher, 2022a).

This highlights that *measurement has advantages over mere categorisation* by *additionally* enabling the descriptive differentiation between divisible instances of the same kind (quality)—between quantities (Hartmann, 1964; Michell, 2012; Uher, 2021c,d, 2022a). In this way, measurement enables more sophisticated analyses of categorised objects and their relations. But it requires appropriate qualitative categorisation of study phenomena, which is far more challenging than the identification of divisible properties in them. Yet both may also go hand in hand as the history of metrology shows (e.g., development of thermometers; Chang, 2004). Hence, ultimately, *all quantitative research has a qualitative grounding* (Campbell, 1974; Kaplan, 1964). The common dichotomisation of ‘quantitative' vs. ‘qualitative' methods, data and approaches reflects a fundamental misconception, implying quantities could be determined independently of the quality studied, yet overlooking that any quantity is always *of something*—a specific quality (Uher, 2018a, 2020a, 2022b, 2023a).

#### Steven's redefinition of ‘measurement' and concepts of ‘scale' types

Obviously, psychical phenomena lack properties that are amenable to concatenation, thus failing to satisfy the *additivity* criterion of quantity (Ferguson et al., 1940). To establish quantitative inquiry in psychology regardless, Stevens (1946) proposed that psychologists should focus not on properties featuring demonstratively additive structures but instead on the structure of the operational procedures that are used for empirical inquiry (Borsboom and Scholten, 2008). For this purpose, he turned to *operationalism* from physics (Bridgman, 1927) and adapted it in his own specific ways (Feest, 2005) by claiming.

“operationism consists simply in referring any concept for its definition to the concrete operations by which knowledge of the thing in question is had” (Stevens, 1935, p. 323).

In line with this, Stevens (1946, p. 667) *defined ‘measurement'* as “the assignment of numerals to objects according to a rule”. This operationalist redefinition formed the basis for psychology's theories and practices of ‘measurement' and separated them from those of measurement used in physics and metrology (Mari et al., 2021; McGrane, 2015; Uher, 2020a, 2021a, 2025).

Many psychologists seem to be aware neither of how fundamental the thus-introduced differences are nor of the epistemological errors on which these are built and that these entailed. For example, Stevens' redefinition promoted the idea that psychology requires a “*soft”, “weak” or “wide” definition of measurement* (Eronen, 2024; Finkelstein, 2003; Mari et al., 2015). Certainly, psychology does not need the high levels of measurement accuracy and precision that are necessary for sciences like physics, chemistry and medicine where errors can lead to the collapse of buildings, chemical explosions or drug overdoses (Uher, 2023a). But simply redefining a scientific activity that is as fundamental to empirical science as measurement is *epistemologically mistaken* because this undermines its comparability across the sciences. Specifically, redefining measurement for non-physical sciences fails to provide guiding principles that specify how genuine analogues can be conceptualised and empirically implemented while appropriately considering the peculiarities of the different sciences' study phenomena. Epistemic comparability is crucial for research on complex real-world problems because integrating findings across different sciences presupposes transparency in their quantitative data generation to enable epistemically valid inferences on the phenomena studied. Labelling disparate procedures uniformly as ‘measurement' also obscures essential and necessary differences in theories and practices between the different sciences as well as inevitable limitations (Uher, 2022a, 2025).

Indeed, following Stevens' redefinition, many psychologists came to understand ‘measurement' as simply any consistent operational procedure of numerical assignment (McGrane, 2015). Many psychologists also know only Stevens' (1946) concepts of ‘*measurement scales'* (nominal, ordinal, interval, ratio)—which likewise depend on operational rules of numerical assignment (Borsboom and Scholten, 2008)—ignoring that these are neither exhaustive nor universally accepted (Thomas, 2019; Uher, 2022a; Velleman and Wilkinson, 1993). Stevens' operationalist approaches offered simple solutions for enabling empirical research and theory development in quantitative psychology. Still today, operationalism is considered an essential feature of rigorous psychological research, where constructs are defined through operational procedures, such as ratings on sets of item statements describing the phenomena of interest (AERA et al., 2014).

Stevens' works also informed one of the first theories of ‘measurement' established in the social sciences as well as psychology's main method for generating quantitative data.

#### Representational Theory of Measurement (RTM)

Representational Theory of Measurement (RTM) formalises axiomatic conditions by which relational structures observable in an object of research can be mapped onto relational structures in a symbolic system (e.g., model with variables and numerical values). It provides mathematical theories for this mapping (*representation theorem*), including permissible operations for transforming the symbolic relational structures without breaking their mapping relations onto the empirical relational system studied (*uniqueness theorem*; Krantz et al., 1971; Luce et al., 1990; Narens, 2002; Suppes et al., 1989; Vessonen, 2017). The theory's focus on *isomorphisms*—thus, on reversible one–to–one relations between observables and numerical data—presupposes that the objects of research feature properties with quantitative relations that are directly observable (e.g., ‘greater than' or ‘less than'). Such relations can be mapped straightforwardly onto a symbolic system that preserves these relations (e.g., ordinal variables; Suppes and Zinnes, 1963).

In psychology's complex study phenomena, however, quantitative properties obviously cannot be identified—the very fact that first led Stevens to focus instead on operational procedures. Psychologists therefore relied on Stevens' concepts of ‘measurement scales', which define types of data variables by their formal properties (e.g., ordering relations, equal distances), thus specifying also the formal transformations (e.g., arithmetic operations) that can be performed in the symbolic relational system. Following the isomorphic relations between the empirical (real) and the symbolic (formal) system stipulated by representational theory—as well as the ‘measurement' jargon used—these merely formal concepts were also ascribed the meaning of 'instruments', analogous to physical measuring devices. Physical measuring instruments (e.g., weighing scales) enable traceable empirical interactions with the *specific quantity to be measured* (the *measurand*; e.g., an apple's specific weight). Instrument, measurand and their empirical interaction are all physical and pertain to the real system under study, whereas the information about them is symbolically encoded in the formal study system (e.g., model with variables and values).

In psychology, however, these crucial epistemic distinctions are obscured because psychological ‘instruments' are language-based—and thus, formal as well (see Topic 3; for details, Uher, 2025). For example, the term *psychometric ‘scales'* is used to denote the items and answer ‘scales' (e.g., five answer categories) presented to respondents (e.g., digitally) as ‘instruments' that are thought to enable interactions with the study phenomena (e.g., respondents' beliefs). In this notion, they pertain to the real study system. But the term also denotes the statistically modelled (latent) structures underlying the response values obtained on many (manifest) item variables (modelling, e.g., probabilistic response patterns). In this second notion, psychometric ‘scales' form part of the formal study system—respondents neither know about nor interact with it (for details, Uher, 2025). Referring to ‘scales' indiscriminately as parts of both the empirical *and* the symbolic relational systems obscures the crucial epistemic distinction between them (Uher, 2018a, 2022b). This also disables the epistemic necessity to specify the relations between them.

The relations between real (empirical) and formal (symbolic) study systems concern one of the most fundamental problems in empirical science. Their specification requires *representation decisions* about what to represent, and what not, and about how to represent this in a formal system (e.g., a model; Harvard and Winsberg, 2022; Uher, 2025). This is discussed as the *problem of scientific representation* in philosophy of science (Frigg and Nguyen, 2021; van Fraassen, 2008), as *encoding and decoding relations* in bio-physics and theoretical biology (Rosen, 1985, 1991), as the *problem of coordination* or *correspondence* in physics (Hempel, 1952; Margenau, 1950), and as *coordination and calibration* in metrology (Chang, 2004; Luchetti, 2020; Tal, 2020). Many psychologists, however, seem largely oblivious of these fundamental issues. Some even consider representation as irrelevant for psychological ‘measurement' (e.g., Borsboom and Mellenbergh, 2004; Michell, 1999)—a consequence and reflection of Stevens' operationalism. Indeed, neither Stevens nor representational theory provide any concepts or procedures for how and why some empirical observations should be mapped to a symbolic relational system (Mari et al., 2017; Schwager, 1991). Rather, they stipulate purely representationalist and operationist procedures focussed solely on the assignment of numerical values with mathematically useful relations.

#### Quantitative data generation with rating ‘scales'

These procedures also underlie psychology's primary method of quantitative data generation—rating ‘scales'—in which numerals (e.g., ‘1', ‘2', ‘3', ‘4', ‘5') are rigidly assigned to the answer categories provided to raters (e.g., five stages indicating levels of agreement). Misled by the premises of an efficient implementation of ‘measurement' in psychology, many overlook even striking errors in their own numerical assignments. Indeed, what justifies the assumption that ‘agree' (assigned ‘4') reflects more than ‘disagree' (assigned ‘2')? Is agreeing with something not rather an entirely different idea than disagreeing with it? How can we assume that ‘neither disagree, nor agree' (assigned ‘3')—thus, having no opinion or finding the item not applicable—constitutes more than ‘disagree' (assigned ‘2')? And why should we assume that the distance between ‘neither disagree, nor agree' and ‘agree' equals that between ‘agree' and ‘strongly agree' (both assigned a distance value of ‘1')? Given the logico-semantic meanings of these verbal answer categories, it is unsurprising that raters interpret them not as reflecting order or even interval relations but only as categorically—thus, qualitatively (nominally)—different. Such logical errors also occur with frequency ‘scales'. Given that occurrence rates generally differ between phenomena (e.g., chatting vs. arguing), rating ‘scales' force raters to indicate a broad range of quantities *flexibly* in the same bounded answer ‘scale'. Raters can do so only by assigning *different* quantitative meanings to the *same* answer value—a necessity that violates core ideas of measurement (Uher, 2018a, 2022a, 2023a, 2025).

Nevertheless, rating data are commonly interpreted as results of ‘measurement' that provide quantitative information about the phenomena of interest (e.g., individuals' beliefs). This contrasts with their purely operationalist generation in which numerical values are assigned to the fixed ‘scale' categories in identical ways for *all* items of a questionnaire, *regardless of the specific study phenomena* to which these may refer. That is, without explanation, raters' judgements of verbal statements, such as their levels of agreement, are re-interpreted as reflecting quantities of the phenomena described. Many psychologists seem to be unaware that this interpretation involves a shift in the underlying philosophy of science because psychological theories and practices build on different presuppositions than the measurement framework established in physics and metrology (Uher, 2020a, 2021a, 2025).

#### Confusion of two incompatible philosophies of science masked by psychological ‘measurement' jargon

Stevens' operationalism and representational theory of measurement are strongly connected to *positivism*, coined in particular by Comte in the 19th century for social science. This family of philosophical theories builds on the presupposition that scientific knowledge should be derived solely from empirical evidence of observable phenomena. Inspired by the successes of the natural sciences, positivists seek to provide accurate and unambiguous knowledge of the ‘world', thought to be objectively given and independent from us, using natural science methods—observation, experimentation, logic and mathematics. Scientists' tasks are to study the facts (thus, focussing on the concrete), to identify regularities in them (therefore focussing on replicability) and to formalise these in (descriptive) laws, whereby explanations often involve no more than subsuming special cases (particulars) under general laws (see Topic 4). Positivists reject abstract theorisation and metaphysical beliefs, which are dismissed as speculative, unobservable and untestable. *Metaphysics*^4^, dating back to Aristotle, is the philosophical inquiry into abstract principles and the first causes of things, covering topics such as ontology (being), space, time, determinism and free will (van Inwagen et al., 2023). The positivists' view that eliminating metaphysics would be desirable, however, is a metaphysical presupposition itself (Bickhard, 2001). Hence, positivism is focussed on description, control and prediction (replicability)—yet at the expense of advancing an ontology of the objects of research and their nature, which limits its ability to develop explanations of them (Al-Ababneh, 2020; Ali, 2023; Howell, 2013).

Physical measurement, by contrast, builds on theories of realism (Mari et al., 2021; Schrödinger, 1964; von Neumann, 1955; Uher, 2025). *Realism* generally is the philosophical perspective that there is a ‘reality' that exists regardless and independently of our perceptions, understanding and beliefs of it. This requires ontological theories about the objects of research and epistemological theories about the ways in which knowledge about these objects can be gained. This general perspective underlies many different forms of realism, each involving different epistemologies (e.g., scientific realism, critical realism) and used in different variants, often reflecting their authors' idiosyncratic qualifications. We do not aim to provide an overview here but select only some that are relevant for our analyses.

Theories of *scientific realism*, for example, involve the presuppositions that both observable and not directly observable parts of ‘reality' exist (e.g., electrons) and that we can explore these with our best scientific theories and models—thus, using both empirical observation and theoretical reasoning. The main epistemic belief is that science aims at providing an accurate, truthful account of ‘reality' so that, with scientific progress, accepted theories are believed to approximate that ‘reality' ever more closely. Specifically, theories are regarded as truthful to the extent that their concepts correspond to the real study system, which underlies the successful use of these concepts for advancing theoretical explanations of these real systems (Chakravartty, 2017; Miller, 2024; Al-Ababneh, 2020).

This pinpoints key differences to positivism where theories are aimed only at describing and predicting observable phenomena, as evident in many quantitative psychologists' focus on replicability, predictive validity and other common quality criteria of mainstream psychology. Therefore, psychometricians who (implicitly) rely on positivist presuppositions often are simply “not persuaded” by the necessity to establish theoretical and empirical relations between the real and the formal study system. They also often refer to realist theories as “axiomatic measurement theory”, implying a metaphysical notion (e.g., Markus, 2021). Yet without systematically conceptualising and empirically connecting the real and the formal study system, results cannot be interpreted as reflecting quantitative information about the phenomena studied in individuals (Rosen, 1985). This lack of epistemic validity contradicts the psychometricians claim to be able to “measure the mind” (e.g., Borsboom, 2005) as well as calls to consider ontological theories in psychological ‘measurement' (Borsboom, 2006). This (implicit) reliance on two incompatible philosophies of science—one for the theories and empirical practices, and another for the result interpretations and declared aims—causes logical contradictions (Uher, 2020a, 2021a,b, 2022a, 2025).

The correspondence between theoretical concepts and empirical observations is central to the *problem of universals*—identified already by Plato, Aristotle and scholars of the medieval university. It concerns the fundamental epistemological question of how we can develop universal categories and trusted knowledge of nature if we can always observe only a finite number of concrete particulars (Klima, 2022). Over millennia, scholars developed many approaches to explore this problem. Our next contribution acknowledges the constructed nature of theoretical concepts and their pragmatic utility while simultaneously endeavouring to establish a systematic mapping to the empirical study system. These presumptions are used to explore theoretical concepts and models of psychological ‘measurement' and to pinpoint the contradictions that are still not well considered.

### Measurement in psychology: A promise that failed to materialise

Psychology's efforts to establish a robust system for measurement have faced profound conceptual, theoretical and methodological challenges. Since the early days of scientific psychology, there has been a tendency to develop ‘measurement' models that are mimicking those used in classical physics (Heene, 2011; Cornejo and Valsiner, 2021). This approach was intended to call for a “natural science infinitely more complete than the psychologies we now possess” (James, 1895, p.124)—thus, for the naturalisation of psychological science. It has become evident, however, that this enterprise has failed. In their measurement-theoretical research, Jörg-Henrik Heine and Moritz Heene highlighted that the most basic approach to measurement involves the simple principle of *counting* units. This requires that a one-to-one relationship is established between the phenomenological object domain and the mathematical metric space of positive real numbers (Heine and Heene, 2025)—the most basic approach to measurement (von Helmholtz, 1887; Hölder, 1901). However, this has never been successfully applied in psychology's entire history.

Why did the promise of metric measurement in psychology remain unfulfilled? Heine and Heene's (2025) critique of the one-sided focus of psychometric models on the numerical relational system highlighted various conceptual, theoretical and methodological issues. These issues cast a merciless light on the deep gap between mathematical models for Θ and the empirical relational system Ψ.

#### Conceptual issues: Misconstrued operationalism and jingle–jangle fallacies

Conceptual issues arise from the inherent complexity of psychological constructs and the empirical problems that this entails (Maraun, 1998). Unlike the natural sciences, where technical concepts are clearly defined and applied by necessary rules, psychological constructs are rooted in everyday language. *Operationalism* (Bridgman, 1927, 1938)—as used in psychology to bridge the gap between Θ and Ψ (Feest, 2005)—has instead deepened it. Originally intended as an “operational analysis” (Bridgman, 1938) to explicate “the meaning-contours of concepts *already in place”* (Koch, 1992, p. 261, emphasis in the original), psychology misconstrued operationalism as a framework for *defining* constructs by naming its (purportedly) quantifiable entities (Koch, 1992; Chang, 1995; Hibberd, 2019). Therefore, psychological ‘measurement' often relies on *nomic measurement* (Chang, 1995, p. 153), whereby unobservable constructs are linked to observable proxies through a-priori definitions and settings. For this reason, it is also called *measurement by fiat*—‘measurement' by decree (Torgerson, 1958, p. 22; Cicourel, 1964, p. 3; Uher, 2020a). This, however, can lead to circular reasoning (van Fraassen, 2008; Chang, 2004, 1995; Luchetti, 2024; Uher, 2021a, 2025). This operationalist practice also resulted in a plethora of *different* ‘definitions' of constructs sharing the *same* term that frequently show only empirically weak correlations with each other (Elson et al., 2023; Pace and Brannick, 2010; Skinner, 1996). It also led, vice versa, to the proliferation of *different* terms for the *same* construct—thus, contributing further to *jingle-jangle fallacies* (Hanfstingl et al., 2024; Kelley, 1927; Thorndike, 1903).

#### Theoretical issues: Fragmented theories and misguided assumptions about measurement and replicability

Psychology is currently debating Questionable Research Practices (QRPs) as potential causes of its replication crisis. But psychology still lacks robust discussions about the Questionable Research Fundamentals (QRFs) of its ‘measurement' concepts, such as the near-exclusive reliance on continuous variable models to explain abstract population-level effects through aggregate statistics (Figure 1). This (still largely) unquestioned practice reflects the widespread misuse of ergodic assumptions, where intra-individual and inter-individual variations are treated as equivalent (see Topic 4; Molenaar, 2008; Speelman et al., 2024). Such an assumption fails to account for the idiographic and developmental nature of psychological processes, where individual differences are crucial (Salvatore and Valsiner, 2010). When unaddressed, this oversight can contribute substantially to psychology's replication crisis.

Psychological research should instead emphasise empirically observable patterns and structures to uncover the underlying idiosyncratic mechanisms and causes of its study phenomena, aligned with an “observation-oriented science” approach (Grice et al., 2012). Some psychological theories, however, have been criticised for inspiring empirical research on hypotheses that are trivial or logically self-evident, thus offering little value to scientific understanding. For example, Bandura's (1977) self-efficacy theory was identified by Smedslund (1978) as a starting point for pseudo-empirical follow-up research. Smedslund demonstrated that the core propositions of the theory could be reformulated into 36 a-priori, non-contingent theorems—thus, statements that are logically provable without requiring empirical validation (see also Smedslund, 1988, 1991, 2016). The motivation for some of these pseudo-empirical research projects may lie in a simplistic logic of justification, which is often seen in the context of educational policy decisions.

Another fundamental issue in psychology is that researchers frequently compare empirical outcomes with one another instead of testing them against a theory to be validated or refuted (Muthukrishna and Henrich, 2019). The Reproducibility Project (Open Science Collaboration, 2015) illustrates this dynamic. As the former NASA scientist Paul Lutus (personal communication with Moritz Heene, 3rd March 2016) put it:

“the Reproducibility Project can be carried out with predictable consequences, then many people will discuss the outcome in great detail without anyone noticing that *the root problem in psychology is that investigators are comparing experimental outcomes with each other, rather than with a theory to be either supported or falsified*. Modern psychology is an intellectual construct in which everything lies at the periphery, but there's nothing at the centre to bind the periphery together. In psychology, and if it were possible, that centre would be a robust theory against which every experiment would be compared, and either a problem with the experiment would be revealed or the theory would be modified or discarded, replaced by a better one, as regularly happens in physics” (italics added).

Unlike physics, where theories (e.g., Newtonian mechanics) provide a foundation for measurement, psychology's reliance on fragmented constructs and study phenomena hampers the integration of its large empirical databases into a cohesive scientific framework (Michell, 2000).

#### Methodological issues: Misapplying natural science paradigms to psychology

Methodologically, an over-reliance on natural science paradigms (naturalisation; Sherry, 2011) has resulted in the use of inappropriate analogies for ‘scales' (Stevens, 1946, 1958). This theoretical gap between the mathematical models and the empirical psychological ‘reality' is further highlighted by the limitations of psychometric models, such as Rasch models (Rasch, 1960). Heine and Heene (2025) criticised the widespread “putting-the-cart-before-the-horse” belief that relying on psychometric models merely as models for numerical relational systems could guarantee genuine interval-level measurements for psychological constructs. Early attempts were made to connect numerical and empirical relational systems (Fechner, 1858, 1860a,b). However, these efforts have been overshadowed by misinterpretations and misapplications of psychometric models—as if their mere application inherently yields interval scales for Ψ. In fact, their mere application generates real numbers for Θ while disregarding the relationship between the numerical and the empirical relational system, thereby potentially misrepresenting the true nature of psychological attributes (von Kries, 1882; Trendler, 2009; Uher, 2021a, 2025). Along those lines, Heine and Heene (2025) highlighted that the Rasch paradox is genuine: the ‘interval scales' created by this item response model are consistent with nothing more than ordinal attributes of psychological variables (also Barrett, 2003; Michell, 2014; Trendler, 2022b). The same applies to conjoint measurement (Trendler, 2019a).

#### Psychology's prospects for quantifying its study phenomena and future directions for novel developments in its methodology

The persistent challenges in measuring psychological phenomena originate from and are perpetuated by conceptual ambiguities, theoretical fragmentation and methodological misconstruals of (especially psychometric) models. Without addressing these fundamental issues, the promise of a robust and scientific measurement framework in psychology is unlikely to materialise. On the other hand, as Schönemann (1994, p. 150) suggested, we may need to accept

“the prospect that psychology will never make much progress towards becoming a quantitative science” *in the sense of measurement in a metric space* also known as the real number line. Instead, psychological methodology must recognize that “…models can also be used that, from the outset, … imply only an ordinal scale level for both Θ and Ψ, such as the ordinal probability models” (Heine and Heene, 2025, p. 22).

The logical contradictions that arise from the positivist theories and empirical practices established in psychological ‘measurement' and the realist interpretations of results and declared aims become obvious in further ways.

### Latent variable models, unit-free ‘measurement' and generative artificial intelligence (genAI) cannot enable measurement: Psychology must consider the peculiarities of human mind

“Science requires measurement”—this belief has become quantitative psychology's unquestioned imperative (Michell, 2003). It builds on Thorndike's credo that everything exists in some amount and can thus be measured (Michell, 2020) as well as on Lord Kelvin's dictum that only what is expressed in numbers constitutes scientific knowledge (Barrett, 2005). In his research papers, blogs and postings, Paul Barrett expressed critical views on psychological ‘measurement' that he had developed in his various roles not just in academic psychology but also in forensic psychiatry and the assessment industry. For 26 years, Paul Barrett maintained the IDANET^5^ mailing list where he regularly informed a growing community of scholars about new publications of both mainstream and non-mainstream research and stimulated thought-provoking discussions on psychology's theories and practices of ‘measurement'.

#### The quantitative imperative and the myth of unit-free ‘measurement' in psychology

Barrett (2005, 2008) advocated for rethinking the entire basis on which psychology generates its ‘measurement' concepts. He highlighted that empirical experimental manipulations of attributes are required before attribute magnitudes can be represented by a real number system, let alone the instantiation of a unit of measurement. Without any empirical evidence suggesting that psychological attributes vary quantitatively (e.g., ordinal and additive structures), we should not make that assumption (Barrett, 2003, 2018; Michell and Ernst, 1996, 1997; Michell, 1997, 1999, 2000; Trendler, 2009, 2013). The most reasonable assumption is that we can assess partial orders or classes with some degree of ‘fuzziness' between boundaries. Yet without any clear methodology for determining precisely how an attribute varies and what is causal for those variations, we are relying upon mere ‘common-sense' judgements of magnitude (Barrett, 2018). Barrett also showed that neither unit-free ‘measurement' nor arbitrary units can possibly sustain a quantity—whether trying to express it as a derived or a base unit^6^ quantity (Barrett, 2011, 2018; Newell and Tiesinga, 2019). This was also elaborated upon by Trendler (2019b, 2022a) for psychological ‘measurement' generally as well as specifically for conjoint measurement and Rasch modelling (Trendler, 2019a, 2022b), supported more recently by Heine and Heene (2025).

So, what remains of decades of research on latent variable models (LVM), hierarchical multilevel modelling (HML) and structured equation modelling (SEM)? Revelle's (2024) article “The seductive beauty of latent variable models: Or why I don't believe in the Easter Bunny” already answered this question in its title. The fundamental problems with psychology's unsupported assumptions of the human mind's measurability are not solved with ever more sophisticated statistical and visualisation techniques. ‘Network psychometrics', for example, merely reifies as ‘explanatory' what is essentially a simple network analysis and data visualisation application but hardly an advance in our understanding and explanation of the human mind. Barrett (2024) demonstrated (e.g., using computer simulations) that no psychometrics or test theory does more than provide general statements of ‘effect' or ‘measurement'.

#### Most branches of mathematics are concerned with non-quantitative structures and provide meaningful concepts for formalisation in all sciences

Contrary to most psychologists' beliefs, studying psychological attributes and phenomena does not require quantitative ‘measures' and not all structures studied with mathematics are quantitative. Mathematics is the science of abstract structure (Resnick, 1997). Most of its branches are therefore non-quantitative, such as pure mathematics, category theory, geometry or set theory, which provide important concepts for formalisation in empirical sciences (Barrett, 2003; Linkov, 2024; Parsons, 1990; Rudolph, 2013). Psychology requires non-quantitative ‘measures'—classes, orders, structured observations and models (Barrett, 2003). These possess a *pragmatic* value or are associated with “good enough” reasoning rather than any sophisticated statistical modelling or deployment of a statistical ‘measurement' model.

It is convenient to use numbers to represent ‘magnitudes' on occasion and to rely upon the arithmetic properties afforded by such use (e.g., in educational assessment). However, it must always be made clear that this numeration is solely for computational convenience rather than enabling any degree of accuracy of a ‘measurement' of psychological attributes—for which Barrett (2003) proposed the term *applied numerics* instead of psycho-‘metrics'.

#### Generative AI and large language models (LLMs) cannot solve psychology's problems: Human minds are complex, open, self-organising systems

Barrett (2024) was an outspoken critic of the increasingly popular attempts in individual differences psychology to use Machine Learning (ML) algorithms for forming machine-generated ‘measures' of personality attributes. He highlighted that, unlike physical scientists, we are not calibrating an alternative measure of length using a previously calibrated measuring instrument (e.g., a ruler or steel tape). Length can be formalised in a quantitatively structured base unit variable—but a personality attribute cannot. Consequently, attempting to predict personality scores with sufficient accuracy and generalisability such that they could be replaced by machine-generated scores using other kinds of observational or ‘digital-footprint' data was never going to work—from the first principles of ‘measurement' let alone the conceptual and known semantic haze of verbal ‘scale' content (see Topic 3).

Human minds are not closed systems that can be manipulated and measured, as one might pursue with mechanistic variables in closed physical systems. Human minds are complex, open and self-organising cognitive systems (Barrett, 2005; Kelso, 1995; Trendler, 2009). Higher-order complex systems feature interconnected parts, non-linear dynamics, emergence, adaptation, sensitivity to initial conditions, feedback loops, equifinality and further peculiarities many of which are not found in inanimate systems (see Topic 1; Barrett, 2024; Uher, 2021c, 2025). The outputs of such systems cannot be accurately predicted, although they can be generalised and classified in terms of broad descriptive phenomenal statements. Many psychologists and technical people working on ‘predictive' models seem to ignore, or be unaware of, the fundamental properties and qualities of the specific study systems whose outputs they are trying to model—that is, those of human beings.

#### Alternative approaches that do justice to the individual: Observation-oriented modelling (OOM)

For causal analyses that do not rely upon assumption-laden statistical parameterisation and metaphorical discussions about ‘unobservable variables', James Grice developed *Observation Oriented Modelling* (OOM; Grice, 2011; Grice et al., 2017a). As with actuarial analytics, the outcome is expressed as “how many cases actually showed the expected or hypothesised outcome? Was this by chance alone? And who were they?”. Paul Barrett also showcased his own actuarial approach to these questions (Grice et al., 2017b; see Topic 4).

Quantitative psychologists cannot hide from their responsibility when, in courts (e.g., US Supreme Court), latent variable or average IQ scores, expressed to two-decimal place precision, are used (even if just partly) to make decisions relating to an offender's death penalty. In many countries, case-law has developed on the basis of popular beliefs about the epistemic authority of psychological ‘measurement' although there is no empirical evidence that the IQ varies as a quantity or indeed as an equal interval attribute. It is just a matter of time until psychometric scores will be challenged in courts, as has previously occurred with forensic psychologists' and psychiatrists' diagnostic practices (Barrett, 2018; Faust, 2012).

The fundamental issues of psychological ‘measurement' and the direct implications that they can have for individuals and society are increasingly discussed also in the public, such as with regard to high stakes testing, admission metrics and policies in educational and occupational assessment. Tackling these issues requires philosophical approaches that enable careful and epistemically justified interpretations of empirical findings.

#### Realist philosophies of science for studying psychical and socio-cultural phenomena

The peculiarities of psychology's study phenomena (e.g., contextuality, socio-cultural constitution, higher-order complexity; see Topic 1) led to the development of further forms of realism. These involve epistemologies that are more appropriate for exploring individual (subjective) and socio-cultural (inter-subjective) interpretations, explanations and appraisals of observable and non-observable phenomena—that is, the meanings that these have for individuals and communities, psychology's central objects of research (Wundt, 1897; Uher, 2025). The existence of these meanings, as individual and socio-cultural phenomena, is conceptualised in realist ontologies. But non-realist epistemologies are used to consider that any scientific inquiry of such phenomena is always situated in a socio-cultural context that influences and shapes the process of inquiry. Moreover, all scientists are human beings themselves with their own personal and socio-cultural perspectives, contexts and frames of reference, which they bring (unwittingly) to their research. Therefore, psychologists cannot be independent of their study phenomena, which entails risks of unwittingly introducing pronounced ego-centric and ethno-centric biases into their research (Adam and Hanna, 2012; Danziger, 1990; Gergen, 1973; Faucheux, 1976; Uher, 2015a, 2020b, 2022b; Weber, 1949).

*Critical realism*, for example, builds on the presuppositions that the social ‘world', just like the material ‘world', features complex structures and that these exist independently of our knowledge of them. In social systems, observable phenomena can be explored for their underlying processes and causes (e.g., human agency). Critical realism emphasises the ‘reality' of the study phenomena and their knowability but also that our knowledge about this ‘reality' is created on the basis of our practical engagement with and collective interpretation and appraisal of that ‘reality'. This allows for reflecting on the relation between the researcher and the researched and for acknowledging that knowledge is theory-laden, socio-culturally embedded and historically contingent (see Topic 1). Hence, critical realism combines a realist ontology with a relativist epistemology, in which diverse perspectives (and even contradictions) are accepted, tolerated and valued (Bhaskar and Danermark, 2006).

*Constructivist realism* is another philosophical perspective that builds on the presuppositions that real-world phenomena (e.g., individuals' intellectual abilities) exist and that their narrated interpretation is intersubjectively constructed and negotiated in the context of their use. It highlights that formal models are human constructions (of analysts) that are used to represent important patterns of complex real-world phenomena in ways that suit the inferences intended. Models necessarily involve abstraction, simplification and idealisation and are studied, in applied work, regarding their aptness for a given purpose rather than simply their truthfulness. Therefore, model-based reasoning involves not just a dyadic relation between a model and real study system but a four-way relation among a model, a situation, a user and a purpose. That is, constructivist realism combines a realist ontology with a constructivist epistemology. It is used in our next contribution to explore meaning-making as a fundamental aspect of psychological ‘measurement' in educational assessment, where it allows for considering multiple socio-cultural meanings of test results, models and applied practices (Kane, 1992; Messick, 1989; Mislevy, 2009, 2018).

### The contextuality of human experience and learning requires a socio-cognitive perspective on psychological inferences in educational assessment

Between-persons Latent Variable Models (LVMs^7^), such as those based on item response theory (IRT), trace back to trait psychology and were advanced, amongst others, through Spearman (1904). Despite their practical value in educational assessment (Lord, 2012), however, a widening gap exists between the LVM conceptualisation and the advances made in cognitive and social psychology to understand learning and acting—including performing in educational assessments. Robert Mislevy argued that a socio-cognitive perspective on LVMs can retain their pragmatic value, while avoiding conceptual errors inherent to current conceptions of LVMs (Mislevy, 2018, 2019, 2024).

#### Latent variable models (LVMs): Key concepts and inherent problems

The kernel of LVMs is the function *f*(*x*_*ij*_|θ_*i*_, β_*j*_). It formalises the probability density of a variable *x*_*ij*_ for evaluated learner performances, given the latent ability variables θ_*i*_ of person *i* and in task (item) *j* characterised by parameters β_*j*_ (e.g., difficulty). The common trait perspective invites taking θs as the persons' measures on a general psychological property Ψ, interpreted through a construct that is assumed to somehow cause the learners' performances *X*s. Conceptual errors often occur because assessment developers and users tend to conflate several distinct elements: the construct itself, the latent variable θ used to operationalise a person's ability, the underlying psychological properties Ψ that the latent variable is intended to represent, and the observed assessment outcomes X. Importantly, LVMs are silent as to the psychological nature of θ and the socio-cognitive processes by which performances arise. Moreover, LVMs often fail to establish the measurement requirements that are necessary to epistemically demonstrate that the psychological property Ψ intended to be studied does indeed exist.

#### The socio-cognitive perspective on educational assessment

The socio-cognitive perspective synthesises research from psychology, linguistics, educational science and complex systems as to the nature of individuals' capabilities and how they develop these through interactions in their social milieu (Gee, 2021; Sperber, 1996). It conceptualises how individuals navigate through situations that are shaped by *linguistic, cultural and substantive regularities of knowledge and action*, which vary over times and contexts. Specifically, individual learners develop cognitive resources to recognise these regular patterns and to act through them. Although individuals are unique, interaction is enabled when individuals' experiences with respect to relevant linguistic, cultural and substantive patterns show similarities, leading to similar cognitive resources.

In any given assessment, individuals blend the particulars of the test situation with the cognitive resources that they have developed from previous experiences in their history of interactions in a cultural milieu. Educators' tasks are to identify linguistic, cultural and substantive patterns that are important for students' learning in order to develop suitable resources (curriculum), to provide the necessary learning experiences (instruction) and to obtain information about students' progress (assessment). By providing conceptual coherence, a socio-cognitive perspective helps to integrate instruction, assessment and real-world practices by explicating and leveraging linguistic, cultural and substantive patterns (Gee, 2008; Harris et al., 2016).

#### Managing evidence, inference and argumentation in LVM-based assessments from a socio-cognitive perspective

This socio-cognitive perspective for assessment necessitates re-conceptions of educational ‘measurement' and LVMs. While psychometric methods and concepts remain useful for differentiating between individuals' performances—from a socio-cognitive perspective—the focus shifts from ‘measuring general psychological properties' to managing evidence, inference and argumentation for making such differentiations. Educational assessment still centres on a *construct* (Messick, 1995) but without being conflated with latent variables, general properties and measures. Here a construct is a *natural language concept*—what individuals can think or say, such as about what they do in situations. These constructs are conceived from a historical, social and cultural standpoint and are framed by assessment designers and users in light of the students, the contexts and the purposes at issue. Task performances are interpreted in terms of choices, approaches and appropriateness as seen from that social standpoint.

The local, unique and multiply-determined socio-cognitive processes that produce learners' performances contrast starkly with the LVM formulation. If not as measurement, how are we to think of the model forms, the probabilities and the variables of an LVM in application? To the degree that a given LVM form and the variables adequately fit the observed *X* values for collections of persons and tasks, a socio-cognitive interpretation is, as a data model, analogous to a mean–field approximation, which replaces many interactions with their average. That is, the fitted model provides probabilities for each observation in the *person*–*task ensemble* via the LVM form and estimated variables. The θs indicate data trends within the LVM form that are associated with persons and the βs indicate data trends associated with tasks. The probabilities given by *f* are interpreted as the *modelers' descriptive probabilities for approximating observations in that person*–*task ensemble*, rather than as probabilities generated by hypothetical extant properties θ of persons and β of tasks. These interpretations of model fit and variables depend on the socio-cultural milieu and personal histories of the individuals in the given ensemble (Byrne, 2002; Gong et al., 2023).

Hence, the contextuality of learning requires a re-conception of LVM symbol systems and their applications by regarding them as *descriptions of patterns in behaviour that emerge from multi-layered socio-cognitive processes*, which are embedded in complex linguistic and cultural contexts. This socio-cognitive perspective provides different narrative structures for organising and reasoning in educational assessment, even from the same learner performances, as they instantiate different arguments. This ontologically and epistemologically more elaborated understanding of LVMs, rather than their common (explicit or implicit) interpretation as reflecting personal properties, will lead to more appropriately—because contextually—grounded inferences in current practices in educational assessment (Mislevy, 2018).

The two previous contributions highlighted that careful, contextualised interpretations of psychometric results, such as using constructivist realist approaches, can enable meaningful applications of psychometric tests for pragmatic purposes in applied settings (e.g., legal, occupational). Psychological ‘measurement', however, is widely used also in academic psychology to study individuals' behaviours, beliefs, abilities and other phenomena and to develop theories about them. Indeed, quantitative data generated with psychometric ‘scales' form the basis of much of the empirical evidence used to test scientific hypotheses and theories in psychology. This requires critical analysis of the ways in which psychometric ‘scales' and models are designed and which determine their appropriateness for empirical inquiry.

Specifically, let us set aside the ontological debate on whether psychical phenomena can have quantitative properties. Assuming they do, what properties must our approaches and methods have to be able to provide the epistemic evidence necessary to support this assumption? In other words, are the current theories and practices of psychological ‘measurement' able to determine quantitative properties of psychical phenomena, if such exist, to warrant their interpretation as procedures of measurement?

### Statistics is not measurement: Psychologists confuse disparate epistemic activities thereby neglecting their actual study phenomena

Psychology's main approach to ‘measurement' involves statistical, especially psychometric analyses, often likened to indirect measurement in physics given the non-observability of others' (e.g., participants') psychical phenomena. But statistics neither *is* measurement nor is statistics necessary for measurement. Physical measurement, even of non-observable properties (e.g., gravity on Earth), was successful long before statistics was developed (Abran et al., 2012; Chang, 2004).

In various transdisciplinary analyses, Jana Uher demonstrated that statistics and measurement involve disparate scientific activities for disparate epistemic purposes. Statistics deals with structural relations in data regardless of what these data represent. Measurement, by contrast, establishes traceable empirical relations between the specific quantities to be measured (the *measurands*) in the study phenomena (empirical or real study system) and the data and results (e.g., true scores) representing information about them (symbolic or formal study system). Hence, statistics concerns purely *syntactic* relations in a data set, whereas measurement also establishes the data's *empirical semantic* meaning regarding the real study phenomena to which these data refer and for which they (symbolically) stand (e.g., Uher, 2021a, 2022a,b, 2025).

#### Psychometrics involves pragmatic result-dependent ‘instrument' design and data modelling, which preclude realist inferences on the actual study phenomena

Psychometric ‘instruments' (e.g., intelligence tests) are designed to discriminate well and consistently between cases (or groups) and in ways regarded important (e.g., social relevance). To achieve this, psychometricians align the structures of psychometric ‘instruments', and those of the data that can be generated with them, to statistical criteria and operations (e.g., normal distributions, internal consistency, item discrimination). The assignment of numerical scores, as well, is aligned to the results' utility and pragmatic value. In intelligence tests, for example, IQ scores are assigned such as to inform about a person's deviation from the age-group specific average, which is set arbitrarily to 100 (and one standard deviation in the normal distribution is set arbitrarily to 15 in both directions). That is, these numerical assignments are aligned to practical purposes rather than to quantitative properties of the actual study phenomena. Indeed, given pronounced cohorts effects (e.g., age groups, Flynn effect; Flynn, 2012), persons with the same test performances may be assigned different IQ scores to enable comparisons with their specific cohort. That is, psychometric theories and empirical practices are designed to generate results with pragmatic utility—they build on a *pragmatist framework* (Uher, 2021a, 2022b, 2023a, 2025).

*Pragmatism* is a philosophical perspective in which knowing the ‘world' is understood as inseparable from human agency and practice within it. This often entails a focus on epistemology and methodology at the expense of ontology. This heterogeneous family of theories and beliefs involves a broad, historically shifting and in parts contrary range of interpretations, which is irrelevant here (Legg and Hookway, 2024). Yet some key features of pragmatism clearly apply to psychometrics. For example, the value of pragmatic research is judged by the effectiveness of its results for a specific problem (e.g., discriminating between individuals) rather than by the results' correspondence to some state of ‘reality'. This contrasts with the various forms of realism, which emphasise the nature of ‘reality' and specify our possibilities and limitations of generating knowledge about it (Mertens, 2023).

Psychometricians' pragmatic focus on the utility and practical consequences of empirical inquiry is evident in the targeted design of psychometric theories and practices to produce quantitative results that are useful for specific purposes (e.g., discriminating between cases). These *result-dependent pragmatic* approaches (Uher, 2021b) contrast with the widespread interpretation of psychometric results as reflecting structures in the actual study phenomena. ‘Personality' or IQ scores, for example, are commonly interpreted as constituting results of ‘measurement' and their quantitative information is attributed to the individuals under study (e.g., their ‘psychophysical mechanisms' or intellectual abilities).

Such inferences, however, can be made *only* when systematic relations are established between the real study phenomena (empirical system) and the measurement results obtained about them in the formal (symbolic) system (Rosen, 1985, 1991; Uher, 2025). This presupposes the *realist framework* of measurement, which, however, is neither theoretically elaborated nor empirically implemented. Instead, psychometrics is centred on modelling data structures in the symbolic study system, whereas the relations between the real and the symbolic study system are being neglected (Uher, 2021a; see also Heine and Heene, 2025). Hence, there is a *gap* between psychometric results and the specific entities that are to be quantified in the actual study phenomena. Bridging this gap requires measurement.

#### Measurement requires data generation processes that are traceable to empirical interactions with the study phenomena and to known quantity references

So, what is measurement? In her transdisciplinary analyses, Jana Uher highlighted that, despite fundamental differences in theories and practices, psychometricians' declared aims and result interpretations reflect basic ideas of measurement that are shared by metrologists, physicists and psychologists alike. These shared ideas can be formulated as two *basic criteria*, which distinguish, across the empirical sciences, measurement from other quantification practices that may be pragmatically useful but lack epistemic authority (e.g., evaluation). These epistemic criteria are (1) the justified attribution of results to the specific entities to be measured (*measurands*; e.g., an individual's duration of speaking in a situation) and (2) the public interpretability of the results' quantitative meaning regarding those measurands (e.g., *how* long that is). These criteria are not meant to classify approaches as ‘superior' or ‘inferior'. Rather, a *criterion-based approach to define measurement* is essential for scrutinising the epistemic fundamentals of a field's pertinent theories and practices, such as to highlight the epistemological inconsistencies inherent in psychometrics, and to pinpoint commonalities and inevitable differences between sciences (Uher, 2021c,d, 2023a).

To meet these epistemic criteria, empirical processes must build on two *corresponding methodological principles*, which underlie metrologists' frameworks of measurement and which are—on their abstract level of consideration—applicable across sciences. Accordingly, measurement requires documented, unbroken connection chains that establish proportional (quantitative) relations of the results with both the measurand's unknown quantity (e.g., in an individual; *principle of data generation traceability*) and a known quantity reference (e.g., international standard units; *principle of numerical traceability*; Uher, 2018a, 2020a, 2021b, 2022a,b, 2023a). These two types of traceability are established in iterative processes of theorising and empirical experimentation in which a real (empirical) and a formal (symbolic) study system, as well as their relations, are coherently related with one another. This *coordination* is crucial for justifying the assumption that a specific procedure does indeed allow us to measure a specific property in the absence of independent methods for measuring it as well as for justifying that specific quantity values are assigned to specific measurands. *Calibration* is used to refine the coordinated structure of a measurement process by specifying the ranges of uncertainties and errors for all its parameters to improve the accuracy of results (Chang, 2004; Luchetti, 2020; Tal, 2020).

**Figure 2:**
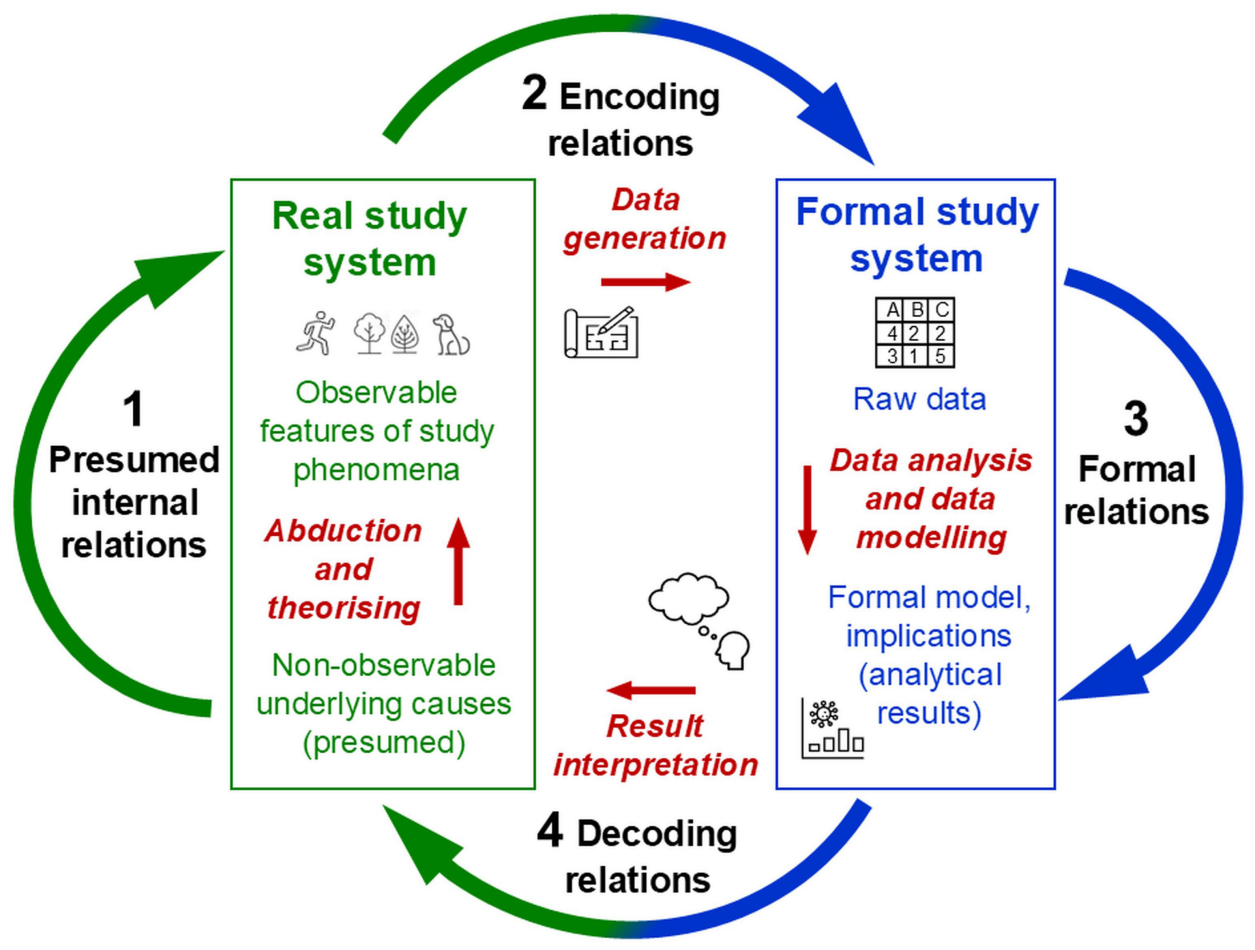
Rosen's general process structure of measurement involves a coherent system of four interrelated modelling relations. It conceptualises the real (empirical) study system and the formal (symbolic) system used for studying it as mathematical objects as well as the processes (mappings, relations) each within and back and forth between them, depicted as arrows. © From Uher (2025), Figure 1; adapted from Rosen (1985).

Rosen's (1985, 1991) general model of measurement conceptualises this process as a *system*^8^ *of four interrelated modelling relations*, comprising the (1) objects of research, (2) data generation (encoding), (3) formal manipulation (e.g., statistical analysis) and (4) result interpretation regarding the objects studied (decoding; Figure 2). This involves modelling the presumed relations within the real study system, comprising the non-observable object of research (measurand), the object used as instrument (including a known reference quantity) and the observable indication produced from their (non-observable) empirical interaction. Their presumed causal relations (arrow 1) are then explored empirically through unbroken and traceable relations to, within and back from the formal system that is used to study that real system (arrows 2, 3 and 4). In iterative feedback loops, the four modelling relations in Rosen's system (arrows 1 to 4) are passed through over and over again, thereby *re-coordinating* and *re-calibrating* them with one another until they are theoretically and empirically coherent, indicating successful modelling of the real study system (for details, see Uher, 2025). Coordinated and calibrated processes enable epistemically justified attributions of the results to the quantities to be measured in the study phenomena (criterion 1) as well as the public interpretability of the results' quantitative meaning regarding those measurands (criterion 2).

Rosen's general process scheme shows that, by focusing on statistical modelling (arrow 3, Figure 2), psychometricians neglect the three other modelling relations (arrows 1, 2 and 4) without which a formal model cannot be coordinated and calibrated with the real study system. Their interrelations are neither conceptualised nor empirically established through traceable connections but simply decreed in psychometricians' result interpretations, declared aims and operationalist procedures of numerical assignments (Uher, 2025).

#### Pragmatic quantifications with predictive power but without explanation

Quantitative psychologists' ‘measurement' jargon alludes to the *epistemic authority* of genuine measurement yet without fulfilling the necessary criteria. This misleads the public, practitioners and scientists because, in both everyday life and science, the term measurement implies that some part of ‘reality' (e.g., a bottle's volume) is being quantified in justified and verifiable ways. Therefore, we trust measurement results (e.g., volume indications on wine bottles; criterion 1) and can interpret (with the relevant knowledge) the specific quantitative meaning that they have for the object measured (e.g., how much ‘75cl' is; criterion 2).

Approaches of psychological ‘measurement' (e.g., psychometrics), by contrast, allow for generating *pragmatic quantifications* that are useful for distinguishing individuals by their observable responses or performances and for making decisions and predictions on the basis of the differences and relations observed. But these approaches do not constitute measurement because they fail to establish coherent relations to the study phenomena both theoretically and empirically. By adapting the ‘instruments' and results instead to statistically useful data structures, these result-dependent approaches cannot explore the observed responses or performances for their underlying causes, such as what specific intellectual abilities individuals may use to show a specific performance in a given task.

The lack of epistemic validity also compromises psychology's efforts to tackle its crises (e.g., replicability). Current initiatives (e.g., robust statistics, replication) solely concern practices focussed on data analysis and interpretation. But psychology's crises cannot be solved without transparency in its data generation (Uher, 2023a). Without advancing *ontological concepts and theories* about the study phenomena (e.g., individuals' thought processes, constructs, behaviours; Uher, 2013, 2015a,d, 2016a,b, 2021c, 2023b) and without elaborating *epistemological and methodological concepts* of how relevant features of these phenomena can be made amenable to quantitative investigation, and if at all (Uher, 2015b,c, 2018a, 2019, 2021d), the root causes of replicable quantitative findings cannot be identified (see Topic 1).

Psychology must tackle the *gap* that often exists between its quantitative findings and statistical models, on the one side, and its actual study phenomena and the specific quantities to be measured in them (measurands), on the other. Therefore, *genuine analogues* of measurement must be advanced for which Rosen's process scheme of measurement and the transdisciplinary concepts of data generation traceability, numerical traceability and the two epistemic criteria of measurement are useful. Clinical research (e.g., on quality of life, chronic disease, therapeutic efficacy) has already pioneered successful implementation of such approaches and advanced their epistemic fundamentals (for details, see Uher, 2025).

This epistemic gap is often overlooked, however, because many psychologists mistake the *inbuilt semantics* of their language-based methods—thus, descriptions of their study phenomena (e.g., in rating scales, item variables, statistical models)—for the phenomena described (Uher, 2025). This shifts our focus to psychology's means of scientific inquiry and their distinction from the study phenomena, as we discuss now in Topic 3.

## Topic 3: Peculiarities of psychology's study phenomena and its means of scientific inquiry: Constructs and language-based methods

Psychology's study phenomena feature peculiarities, such as emergence of novel properties that feed back to and change the very processes from which they emerge in multi-level feedback-loops, leading to continuous change and development and thus, to higher-order complexity (see Topic 1). Such peculiarities are not known from the non-living ‘world' studied in physics and metrology. Moreover, psychology explores not just objects and relations of specific phenomena in themselves (e.g., behaviours) but also, and in particular, their *individual (subjective) and socio-cultural (inter-subjective)* perception, interpretation, apprehension and appraisal (Wundt, 1897; Uher, 2021c, 2025). These complex study phenomena are described in *constructs*.

“A *construct* is a conceptual system that *refers* to a set of entities—the *construct referents*—that are regarded as meaningfully related in some ways or for some purpose although they actually *never occur all at once* and that are therefore considered only on more abstract levels as a joint entity (italics as in original; Uher, 2022b, p. 14).

All humans develop and intuitively use constructs in everyday life (Kelly, 1955, 1963). Everyday psychology is replete with constructs, which are encoded in everyday language (Vygotsky, 1962). That is, constructs form an important part of our human thinking. Constructs are also important conceptual means of scientific inquiry in psychology (e.g., ‘intelligence', ‘leadership', ‘benevolence') and the social sciences (e.g., ‘power', ‘democracy'). Each construct refers to a theoretical universe of referents that are jointly considered for a purpose (e.g., evaluation, explanation) and from a specific viewpoint (e.g., normativity, specific theory) but that can never be observed all at once—constructs are *multi-referential* conceptual systems. For empirical studies, a manageable subset of referents is chosen to serve as *indicators* (Uher, 2022b, 2023b). To conceptually handle constructs, given their level of abstraction, language plays a crucial role in their description and empirical investigation. The distinction between constructs and their referents (e.g., empirical indicators) as well as the intricacies of human language, however, involve complexities that present unparalleled challenges to quantitative inquiry.

### Psychologists' cardinal error: Confusing ontological with epistemological concepts

In her transdisciplinary research, Jana Uher highlighted that, ontologically, all phenomena can be described in their being. To elaborate how knowledge about a given study phenomenon can be gained, thus epistemologically, scientists must decide, in every study, which specific phenomena they aim to explore and which ones they use as epistemic means for exploring these study phenomena. The necessity of this epistemic distinction, first recognised in quantum physics (Heisenberg, 1927), is not well considered in psychology (Uher, 2025). Moreover, this distinction is particularly intricate in psychology given the *anthropogenicity of science*—the fact that all science is made by and for humans using the abilities of the human mind (e.g., conceptualising, generalising, abstracting; Uher, 2022b, 2023a,b). Empirical science is experience-based by definition (from Greek *empeiria* for experience). For scientists exploring mind and experience, this complicates the logical distinction between the specific psych*ical* (e.g., mental) phenomena that they aim to study as their objects of research (e.g., participants' beliefs, abilities, folk constructs) and those psychical (and further) phenomena that they use as epistemic means to investigate the study phenomena (e.g., psychologists' own inferences, theories, methods, Big Five constructs). These epistemic means of inquiry are properly termed psych*ological*^9^, derived from Greek *-logia* for body and theory of knowledge (Lewin, 1936; Uher, 2021b, 2023a).

Failure to make the crucial epistemic distinction between the study phenomena and the study means (in a study) entails the confusion of ontological with epistemological concepts—therefore, it is termed *psychologists' cardinal error* (Uher, 2022b). This logical error makes the distinction of disparate scientific activities (e.g., theoretical vs. operational construct definition) technically impossible, thereby distorting scientific concepts and procedures (Uher, 2013, 2015a,b,c, 2023b). This error can occur in various parts of the empirical research process.

#### Conflations of the study phenomena with the study means masked and perpetuated by psychological jargon

Psychologists' cardinal error occurs when psychologists use key terms ambiguously (e.g., ‘constructs', ‘variables', ‘attributes'), thereby *conflating the study phenomena with study means*. Constructs, for example, are often mistaken for the study phenomena to which they refer (*construct–referent conflation*; Lovasz and Slaney, 2013; Maraun and Halpin, 2008; Maraun and Gabriel, 2013; Slaney, 2017; Uher, 2013, 2021a,b). This leads many to confuse the abstract concept of ‘intelligence' with the various intellectual abilities to which it refers and that never occur all at once but that are just jointly considered for some purpose. This logical error is promoted by the operationalist idea that a study phenomenon's theoretical meaning could be established through the empirical operations that are used to investigate, manipulate or elicit it. Specifying operational procedures may help to pilot conceptual research. But ultimately, operational specifications must be replaced by proper theoretical definitions of the study phenomenon (Green, 2001; Feest, 2005). If these distinctions are not made, further logical errors occur. For example, when reasoning ability is operationally ‘defined' as test performance, this ability cannot also be used to explain this performance. A phenomenon cannot be defined by its effects. Such assumptions *conflate cause with effect*, thereby *turning the effect into its cause* (Hibberd, 2019; Uher, 2022b).

These logical errors also occur when—misled by the availability of single word terms (e.g., ‘personality')—researchers treat constructs as real entities, thereby turning abstract ideas into things (entification, reification, hypostatic abstraction; Peirce, 1958, CP 4.227). This occurs, for example, when ‘personality' constructs are interpreted as entities residing in individuals (e.g., ‘psychophysical mechanisms') that causally underlie their behaviours, feelings and thinking—thereby *turning the description of study phenomena into their explanation* (Uher, 2013). Further logical errors arise from intricacies of human languages that are not well considered in psychology.

#### The intricacies of human languages

Language is humanity's greatest invention (Deutscher, 2006). With words, we can refer to objects of consideration even in their absence (meaning), although what we say or write (signifiers) typically bears no inherent relations (e.g., resemblance) to the objects referred (referents). This representational function of language is built into its *semantics*—the rules that specify the meanings that words, phrases and sentences conventionally convey in terms of what they refer to and stand for in the real ‘world' (their referents). The complex rules of languages (e.g., semantics, syntax, pragmatics)—developed in socio-linguistic communities and internalised during language socialisation—mediate and shape intra-individual and inter-individual processes (e.g., thinking, interacting). Therefore, language and psyche are inseparable from one another, while still constituting different kinds of phenomena (Peirce, 1958; Uher, 2015a,b, 2016a, 2018a; Valsiner, 2000, 2007; Vygotsky, 1962). Because of this entanglement, we do not perceive our words just as tokens of the objects to which they refer but as these objects themselves. Therefore, in our minds, we easily mistake the word for the thing, the map for the territory, the menu for the food—the ‘world' as it is with the ‘world' as it is thought about and described (Uher, 2025).

Our human tendency to mistake verbal descriptions for the phenomena described leads to further instances of psychologists' cardinal error. These occur when researchers—distracted by the ease of using language and unaware of its inherently representational nature—focus only on the *inbuilt semantics* of language, thus on the meanings that words and statements generally have (Uher, 2025). This often obscures the epistemic necessity to distinguish the study phenomena (e.g., individuals' feelings) from their verbal description in the language-based methods used for studying these phenomena (e.g., item ‘scales', variable names), leading to the confusion of ontological and epistemological concepts. This cardinal error often underlies evaluations of face validity and content validity of psychometric ‘instruments'. It also underlies the widespread *nominalism* in quantitative psychology—the belief that any method that is *nominally (by name)* associated with a study phenomenon could be epistemically valid for empirically studying it (e.g., ‘anxiety scale', ‘openness scale'). This contributes to the proliferation of overlapping ‘scales' (e.g., various ‘anxiety scales') and of the likewise overlapping constructs that their items are meant to operationally define (Sechrest et al., 1996; Toomela, 2010; Uher, 2021b, 2022b).

The *inbuilt semantics* of language also often leads psychologists to misinterpret raters' judgements of verbal statements as measurements of the phenomena described in those statements. The epistemic necessity to establish traceable coordinated and calibrated relations between the symbolic and the empirical study system gets out of focus (Uher, 2025). This entails the risk of replicating just verbal descriptions instead of exploring the real phenomena for which these are meant to stand. Therefore, quantitative psychology is at risk of doing *pseudo-empirical* research, which mostly re-discovers what is necessarily true given the logico-semantic relations built into its language-based methods (Arnulf et al., 2024; Shweder, 1977; Shweder and D'Andrade, 1980; Smedslund et al., 2022; Smedslund, 1991, 2016). Indeed, many overlook that human languages have socio-culturally constructed structures and meanings, which do not derive from the ontic ‘reality' that they describe and which therefore vary considerably between languages (Deutscher, 2010; Boroditsky, 2018; Uher, 2025).

This also entails challenges also for philosophy of science. For example, some realist perspectives explicitly involve the presupposition that ‘reality' is “mind-independent” and “language-independent”. These terms, however, if taken literally, may create the illusion that minds and languages could be generally independent of and thus, extraneous to ‘reality' rather than forming part of it as well. This is particularly misleading for psychologists who aim to explore the ‘reality' of mind and whose primary means of empirical inquiry is language, which, moreover, is internalised in human minds. Instead, it is crucial to specify, which parts of ‘reality' are meant to be studied and which parts of ‘reality' are used as epistemic means for exploring these study phenomena—thus, to distinguish ontological from epistemological concepts (e.g., psych*ical* from psych*ological*; Uher, 2023a).

To scrutinise the *epistemic role of language* in empirical inquiry, it is important to ontologically study its elements, structures and relations. Linguists, information scientists, artificial intelligence researchers and other scholars established *ontologies of language* that describe its syntax and inbuilt semantics (e.g., using digital networks), such as those underlying natural language processing (NLP) systems and large language models (LLMs). Our next contribution demonstrates how language ontologies can elucidate some key problems in quantitative psychology and highlights fundamental issues still hardly considered.

### The semantic representations of psychological phenomena reappear in statistical data as self-reinforcing ontologies

All scientific psychological phenomena have in common that they also exist as linguistically defined topics of research. Most psychological constructs also appear as topics in everyday conversation and public discourse. The relationship between psychologically theorised and linguistically defined ‘constructs', on the one hand, and their purported ontological ‘reality', on the other, remains elusive. It has regained importance, however, through the development of digital language processing techniques, as Jan Ketil Arnulf and colleagues documented in their line of research around the Semantic Theory of Survey Response (Arnulf et al., 2014, 2018).

#### Constructs as representations in language models

While early 20th century psychology displayed a sound scepticism towards ‘mentalistic' concepts as legitimate objects for scientific scrutiny, the behaviourist reaction equally created overly strict criteria for legitimate research topics. In the 1950s, the American Psychological Association (APA) accepted in its methods standards the adoption of ‘latent constructs' to the extent that these could be legitimised by statistical modelling techniques (Slaney, 2017). Since then, the domain of psychology has expanded with a growing range of non-observable phenomena that mainly exist through their statistical properties in empirically collected data (Larsen et al., 2013; Lamiell, 2013, 2019a; Smedslund, 2021).

However, theoretical doubts about the ontological status of such constructs and their purported relationships have repeatedly been raised. Most importantly, it has been shown that their empirical relationships, in many cases, may be not empirical but pre-given through their logical or semantic relationships—and thus, pseudo-empirical and tautological (Semin, 1989; Smedslund, 1991, 2012, 2016).

These concerns have rarely been addressed so far. Instead, ever-increasing statistical sophistication and primarily language-based methods (e.g., rating ‘scales') have been used to establish ever more ‘latent constructs' in psychology. This has continued without ascertaining the nature of the phenomena and processes involved in generating the data that serve as input to the statistical models. With the emergence of natural language processing algorithms and software, this concern has now been turned into an empirical investigation. It is possible to use verbal ‘measurement scales', variables and construct definitions as well as other methodological features as input to text algorithmic analysis (Arnulf et al., 2021). These technologies were originally built on Latent Semantic Analysis (LSA) but have later become much more precise through the adoption of more advanced language models, such as BERT (Bidirectional Encoder Representations from Transformers).

The key point of this approach is that psychology has overlooked how language itself is describable as having mathematical features. The mathematical features of meaning in language are precisely what enable the powerful large language models (LLMs) that are now ubiquitously available (Devlin et al., 2018; Landauer and Dumais, 1997)—often referred to as “Generative Artificial Intelligence” (genAI; Chang et al., 2024). The semantic approach to the measurement problem in psychology is that the sampled statistics will easily reflect *what we say about* a phenomenon—rather than the phenomenon itself—unless special attention is taken to avoid it (see Topic 2).

The empirical proof of this claim is built on the fact that digital text analysis allows the replication of statistical psychometric models using only textual data as inputs and without any involvement of research participants using these verbal ‘scales' to make quantitative assessments—thus, without any empirical investigation. It is possible to show that much of the systematic information captured by psychometric modelling stems from the semantic patterns of construct definitions and verbal ‘measurement scales' as well as from their mutual relationships (Arnulf and Larsen, 2021; Arnulf et al., 2024).

#### Statistical features of constructs do not make them true or false

Semantically derived findings have two problematic implications for science: first, they are predictable a-priori (Wierzbicka, 1996; Smedslund, 1978, 2016) and therefore do not expand our knowledge. Second, their empirical status remains untested because it is possible to make both true and false statements in language. One such implication occurs in cross-cultural studies on leadership where it was found that propositions about leadership correlated in the same way across the ‘world', even if local behaviours by people in workplaces might be very different (Arnulf and Larsen, 2020).

From a measurement perspective, it can be shown that the quantitative information (data) commonly used to legitimise the ontological status of many ‘latent' psychological constructs does not stem from some unobservable psychological study phenomena. Instead, the quantitative relationships are features of the linguistic structures that we use to represent these study phenomena in operationalisations and variables (Arnulf et al., 2018). When this happens, psychometric models reflect the ways in which researchers and participants describe human experience, emotions, thinking and other psychological phenomena. Ascribing these statistical properties to independently existing phenomena extraneous to language is an error of category, mistaking the representations for the represented—the menu for the food (Arnulf et al., 2024).

#### The human struggle to discern empirical from semantic problems

**Figure 3:**
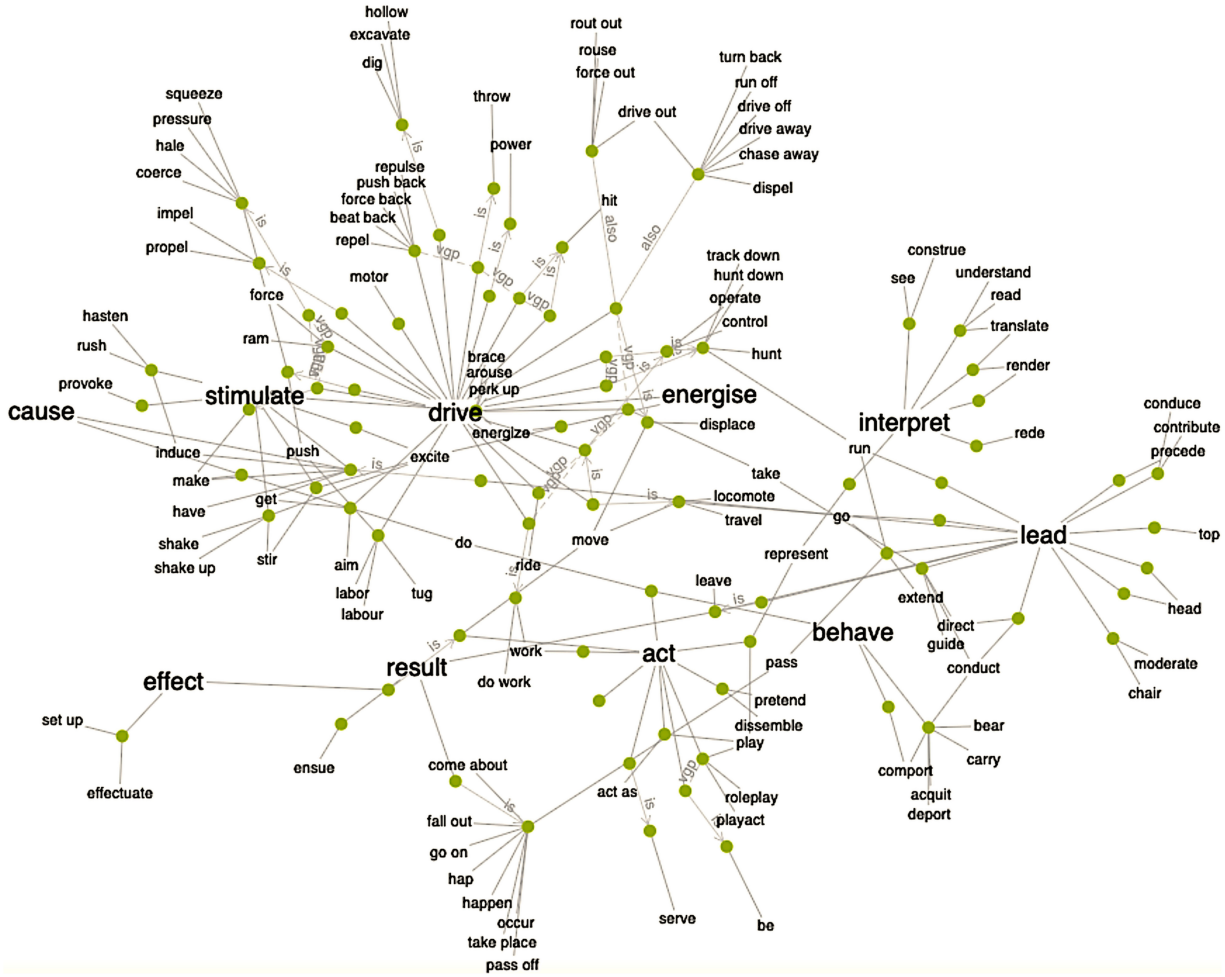
Semantic networks Natural language terms mutually define each other within chains of semantic relationships. When used in language-based methods, such as in descriptions of the study phenomena in rating items, semantic relationships were shown to reappear as ‘explained variance' in statistical research models.

What makes this error practically possible may be the social construction of human ‘reality', turning many constructs into realities by simply treating them as real entities (reification, entification). This obstructs our view of many such constructs as historically developed, belonging to socio-cultural, professional or other communities of practice. However, it can be shown that this semantic nature of the subject matter effectively locks psychological research in mutually defining semantic networks, which can be visualised in graphical networks (for an example, see Figure 3). The conventions of factor analysis restrict the explained variance of its results to an average of 42%, above which explanations appear as auto-correlations and as uninteresting if they become much lower (Smedslund et al., 2022). Since the 1950s, the combination of construct validation conventions and semantic networks has turned psychological research into a self-perpetuating machine that keeps explaining semantic phenomena by rephrasing them as other constructs or other operationalisations instead of tapping into their underlying realities—a mistake of categories.

Within this natural language processing (NLP) paradigm, now enabled through powerful algorithms and software systems, one of the most pressing psychological research questions is to explore why humans in general—and researchers in particular—lose sight of the semantically given frameworks of our socio-linguistically constructed ‘world' so easily. As psychologists, we must better understand why we struggle to differentiate empirical from semantic research problems. This opens up novel perspectives on psychology's crises in replicability, validity and generalisability as well as on the role that psychologists themselves may play in their perpetuation.

All words have meaning. The meaning of every word is a construct (Vygotsky, 1962). Exploring the role of constructs in psychological research requires an elaborated *ontology of constructs* (Kelly, 1955, 1963; Uher, 2023b). Constructs are also studied outside of psychology, such as in information science as in our next contribution, which analyses constructs using Mario Bunge's philosophy. Bunge advocated for scientific realism, positing the existence of a “mind-independent^10^” ‘reality' that can be known and described, at least up to a point (Bunge, 1977, 1993). Through experience, reason, imagination and criticism, we can obtain some truthful knowledge about this ‘reality', which, although variously problematic (e.g., abstract, incomplete, fallible), can also be improved (Bunge, 1993; Cordero, 2012; Mahner, 2021). Bunge elaborated a *materialist ontology*, founded on the presupposition that the real ‘world' (what exists) is composed only of material things. Things can change (construed as events) and possess properties that characterise them. Interactions between things form systems that have novel emergent properties. The real ‘world' is therefore a ‘world' of systems. Bunge conceptualised the ‘mind' not as a thing but as mental properties of complex brains, which emerge from processes of neuronal systems. This *emergentist materialism* thus rejects a dualist body–mind ontology (Bunge, 1981; Mahner, 2021). Our following contribution applies Bunge's ontology to elaborate on constructs and their relations to their indicators as well as to the ‘instruments' that are used for empirical explorations.

### An ontological analysis of construct–indicator and indicator–instrument relationships: Novel theoretical perspectives on current controversies

Constructs and their indicators are central to theory building and theory testing in many disciplines. *Theories* articulate relationships among constructs. *Indicators* are used to measure construct values. Yet the nature of constructs and the relationships among them as well as the nature of indicators and the relationships between constructs and indicators remain contested. The controversies that have occurred are unlikely to abate until the ontological assumptions that underpin constructs and indicators are surfaced and scrutinised (Bagozzi, 2011; Borsboom, 2005; MacKenzie et al., 2011). In this light, Ron Weber used Bunge's (1977, 1979) materialist ontology to analyse the essential nature of constructs and indicators (Weber, 2012, 2021). He chose Bunge's ontology because it is comprehensive, formalised and widely used (Matthews, 2019).

#### Ontological fundamentals: Objects, things, constructs and properties

The fundamental unit in Bunge's ontology is an *object* defined as “whatever can exist, be thought about, talked about, or acted upon. The most basic, abstract, and general of all philosophical concepts, hence undefinable. … Objects can be individuals or collections, concrete (material) or abstract (ideal), natural or artificial” (Bunge, 2003, p. 199). He divides objects into two ontological categories: things and constructs. *Things* are objects in the ‘world' that exist independently of their perception and conception by sentient beings (which are things themselves as well). *Constructs* are objects that exist in sentient beings' brains. As sentient beings, we cannot perceive the ‘world' directly; we perceive it only though our constructs. Hence, whenever we talk about things, we actually talk about our *models* of things—the constructs that we use to comprehend the ‘world'.

The traits that characterise a thing or construct are its *properties* (Bunge, 1977). Two types of properties exist in relation to things. *Properties in general* are common to a class of things. For instance, scholars might study a general property called “benevolence” and the extent to which it is possessed by a class of humans called “managers” (Serva et al., 2005). *Properties in particular* are the specific levels (values) that specific things in a class possess of a given general property. For example, the specific level of benevolence (e.g., “high”) possessed by a specific manager called “Jane” is the particular property of a specific thing from the class called “managers”. Weber (2012) argued, however, that, during theory building and testing, scholars often unwittingly tend to use the term “construct” in a more specific way than Bunge and use it to mean a *property in general* of a thing.

#### The ontological nature of indicators and their relationship to constructs

During theory testing, some focal constructs (properties in general) can be measured directly (e.g., a person's height with a ruler). Often, however, focal constructs are unobservable and must be measured indirectly. Indirect measurements of constructs occur via indicators, which are sometimes observable proxies for the unobservable focal construct (e.g., weight as an indicator of a person's stress level; Bunge, 2010). In psychology and the social sciences, however, indicators are often unobservable in themselves as well. Therefore, they must also be measured indirectly (e.g., managers' typical ways of acting over some time). Nonetheless, scholars might deem that using a set of indicators that can be measured only indirectly (and combining them in some way to determine the focal construct's value) provides the best measure of that focal construct.

Using Bunge's ontology, Weber (2021) argued that scholars predominantly conceive indicators, often unwittingly, as general properties of some class of things. For instance, scholars might study the focal construct “benevolence” as a general property of a class called managers, and they might choose another set of general properties as indicators of that construct to obtain an indirect measurement of it. Indicators of the focal construct “benevolence” might be managerial actions, such as looking out for important issues, ascribing importance to needs and desires and going out of the way to help (Serva et al., 2005). The specific level of “benevolence” for Jane as a specific manager (particular property) will be determined on the basis of her specific levels measured for each of these three indicators.

#### The ontological nature of instruments and their relationships to construct indicators in measurement

To measure the values of indicators for specific things, such as for specific persons (i.e., particular properties of a particular person), scholars use *instruments*. Under Bunge's ontology, instruments are also things with properties. For instance, a questionnaire^11^ (a thing) for studying the focal construct “benevolence” (property in general) of managers (things) might have several manager-descriptive indicators comprising item statements with Likert rating ‘scales'. The item statements themselves (without any specific Likert ‘scale' rating) are properties in general of the questionnaire instrument (thing). Observers (e.g., a manager's subordinates) make judgements about the levels of these indicators (properties in particular) on the basis of their perceptions of their manager's actions. Three such indicators might be “looks out for important issues”, “ascribes importance to needs and desires” and “went out of the way to help”. Subordinates use these indicators with the Likert ‘scales' to rate their perceptions of their manager's actions. The indicators with specific Likert ‘scale' ratings (e.g., “3”, “6”) are the questionnaire's properties in particular.

Ideally, the values that an indicator (or set of indicators combined) assumes for specific things should be *isomorphic* with the values that the focal construct assumes for these things (Borsboom, 2008). In this regard, ideally, an *auxiliary theory* should have been developed to explain why specific indicator values obtained via a measurement instrument are isomorphic with the focal construct's values (Bunge, 1974, 1975, 2010; Edwards, 2011).

#### Property scopes, property pre-orders and measurement instruments

Scholars strive to design and use high-quality instruments to measure the particular properties of specific things (e.g., specific behavioural actions of specific persons)—the measurands. Therefore, many method researchers focus on developing instruments that produce ‘valid' and ‘reliable' measures of focal constructs (Straub et al., 2004). Weber (2021) argued, however, that this literature is fraught with ambiguities and inconsistencies. Moreover, some approaches to measurement are highly contested—for example, whether formative instead of reflective indicators^12^ should ever be used to measure the value of constructs (Bollen and Diamantopoulos, 2017; Guyon, 2018; Hardin and Marcoulides, 2011).

**Figure 4:**
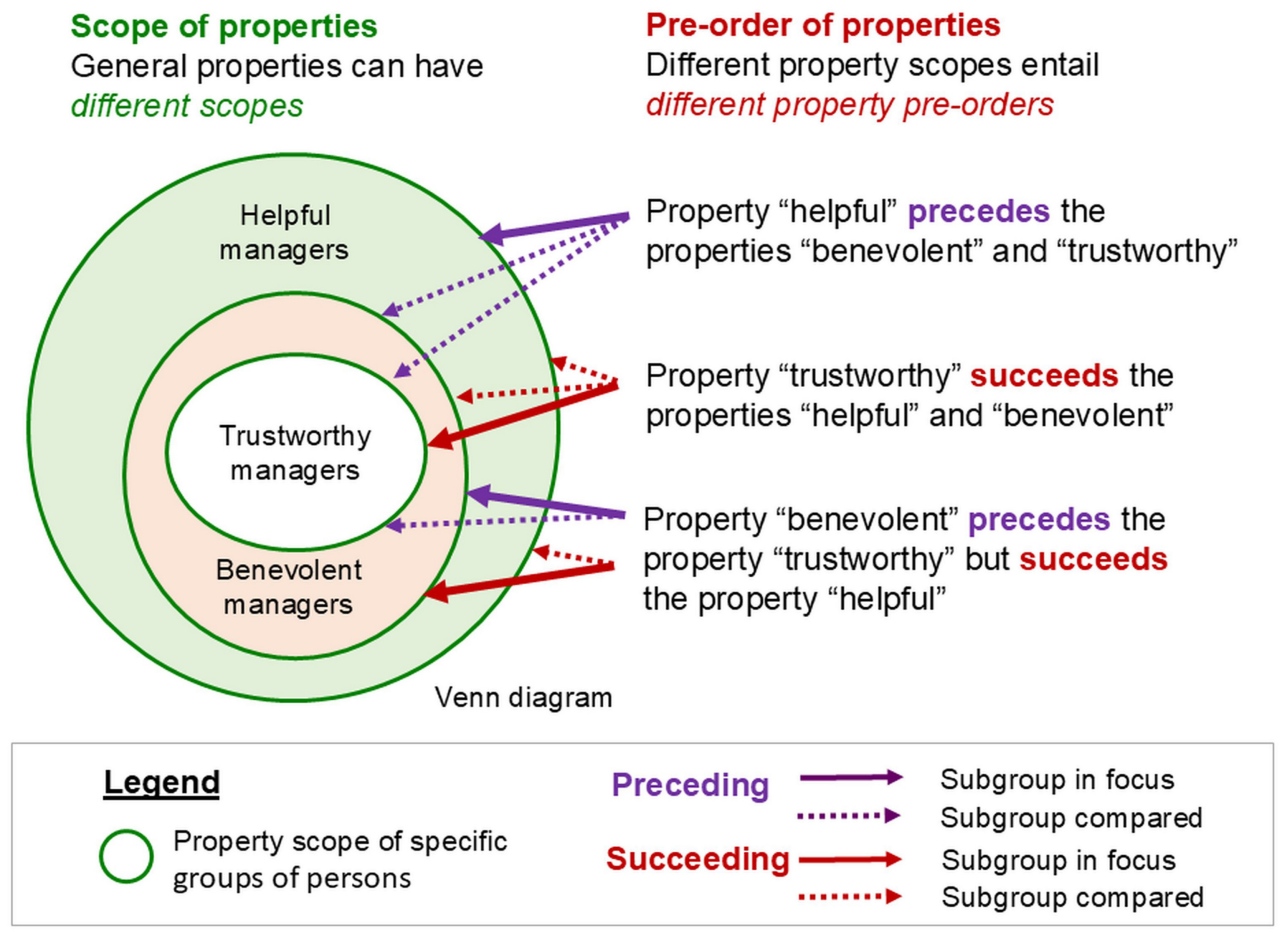
Property scopes and property pre-orders associated with subclasses of things (managers). The different scopes of properties enact different pre-orders on these properties. The property “trustworthy” applies to a subclass of all managers who are “benevolent” of all those managers who are “helpful”. That is, the property “benevolent” precedes the property “trustworthy” and succeeds the property “helpful”. Adapted from Weber (2021).

Weber (2021) proposed a new way to conceive and choose indicators on the basis of Bunge's ontology and Bunge's notion of the *scope of a property*, which is the set of all real-world things that possess that property. For instance, the scope of the property “benevolence” is the set of all individuals who possess it (at some level). If the scope of a property is a single thing, however, the property is possessed only by that thing (it is unique to that thing). Because different properties have different scopes, they apply to different subclasses of things. In a given class of things, these scopes therefore enact a *pre-order* (reflexive and transitive) on the given properties (Bunge, 1977). For example, in a putative theory about “manager trustworthiness”, the scope of the property “benevolence” might be hypothesised to be a *subset* of the scope of the property “helpful” (Serva et al., 2005). That is, *some* but not all managers who go out of their way to help others are also “benevolent” (necessary condition), whereas *all* managers who are “benevolent” also go out of their way to help (sufficient condition). In Bungean terms, the property “helpful” *precedes* the property “benevolence” and the property “benevolence” *succeeds* the property “helpful”. Property scopes and the property pre-orders that they entail can be visualised in Venn diagrams (Figure 4).

Importantly, Weber (2021) showed how the notion of property scope motivates new ways to assess the quality of a set of indicators, such as their *scope validity*. Specifically, if the set of indicators used to measure a focal construct *precede* that construct, ideally the *intersection* of the scopes of these indicators will equal that focal construct's scope. Alternatively, if the set of indicators used to measure a focal construct *succeed* that construct, ideally, the *union* of the indicators' scopes will equal that construct's scope. Weber highlighted that the importance of scope validity is primary to the importance of traditional instrument validity and reliability measures. That is, if an instrument does not have scope validity in the first place, its use can lead to “false positive” and “false negative” outcomes, although the instrument might have high levels of convergent and discriminant validity.

#### Choosing indicators on the basis of property scopes and property pre-orders

When designing or choosing an instrument, scholars must evaluate carefully whether the indicators precede or succeed the focal construct in the pre-order of the properties included by that focal construct. They must then try to determine these indicators' likely scope. If scholars conclude that the intersection of the scopes of preceding properties and the union of the scopes of succeeding properties do not equal the scope of the focal construct, the designed or chosen instrument might not yield valid measures of that focal construct (Weber, 2021).

These ontological concepts from information science can provide novel perspectives also for one of quantitative psychology's most pervasive problems—the approaches for generalising findings across individuals that we discuss now in our next Topic 4.

## Topic 4: Psychology's approaches for generalising findings across unique individuals: Common errors and epistemically justified alternatives

The question of how we can develop general knowledge and universal categories given that we can always observe only particulars—the problem of universals (see Topic 2)—is of specific relevance for psychology as a science studying unique individuals. Quantitative psychologists, especially those building (implicitly) on positivist approaches (see Topic 2), commonly use statistical sample-level findings to generalise across individuals. Epidemiologists and health scientists, by contrast, are long wary of different types of fallacies that inferences from groups (on different levels of aggregation) to single cases, and vice versa, may entail (Diez Roux, 2002). Quantitative psychologists, however, seem still oblivious of the problematic fundamentals on which the use of sample-level statistics for studying individual-level phenomena are based. Therefore, let us first scrutinise the underlying methodological, epistemological and ontological presumptions.

### The overlooked non-ergodicity of psychology's study phenomena: Why sample-level statistics cannot enable individual-level explorations

The advent of the assessment industry (e.g., in the American military in WWI; Gould, 1996), group-based experiments (Danziger, 1985a), rating methods (Thurstone, 1928; Likert, 1932) and statistical advances (Michell, 2023; Spearman, 1904) shifted psychologists' original focus on analysing psychical processes in individuals—psychology's *theoretical unit of analysis*—to analysing distribution patterns in populations, which became psychologists' primary *empirical unit of analysis*. Now, results were presented as aggregate data obtained from many individuals (e.g., group averages) yet without analysing individual patterns (Danziger, 1985b; Lamiell, 2019b). Still, psychologists continued to interpret their findings with regard to single individuals, which remained their focus of interest and *theoretical unit of analysis*. Personality psychologists, for example, commonly equate between-individual differences with individuality (‘personality') and use sample-level statistics (e.g., factor analysis) to ‘study' intra-individual functioning and development (e.g., using the Five Factor Model of ‘personality'; Lamiell, 2013; Uher, 2018c, 2022b).

Inferences from sample-level findings to individual-level phenomena presuppose *ergodicity*—a property of stochastic processes and dynamic systems, which involves that their elements' synchronic and diachronic variations are statistically isomorphic. Ergodicity fits all invariant phenomena, which do not change and develop and in which simultaneity and successivity are therefore statistically equal (e.g., in some inanimate systems). Human individuals, however, are not all the same. Individuals, and the phenomena studied in them (e.g., behaviour, experience, language), vary, change and develop—thus, they change momentarily and over periods of time both intra-individually and inter-individually. Almost a century ago, the mathematicians Birkhoff (1931), John von Neumann and others advanced *ergodic theory*, a branch of mathematics originating in statistical physics (Gray, 1988). Using classical mathematical-statistical (ergodic) theorems, they proved that sample-level findings (e.g., group comparisons or correlations) can be generalised to single cases (e.g., individuals) *only if* (1) each case obeys the same statistical model (*homogeneity assumption*), and (2) the statistical properties (e.g., factor loadings) are the same at all points in time (*stationarity assumption;* Molenaar and Campbell, 2009). Why did ergodic theory elude quantitative psychologists, despite their keen interest in implementing mathematical-statistical approaches analogous to the physical sciences (Uher, 2022b)?

Presumptions of ergodicity are logically necessary for sample-to-individual inferences as well as pragmatically and methodically convenient. But they are invalidated already by ordinary everyday experience—not to mention an established body of empirical and theoretical research in psychology (e.g., Molenaar, 2004, 2008; Molenaar and Campbell, 2009; Richters, 2021; Salvatore and Valsiner, 2010; Speelman and McGann, 2020; Valsiner, 2014b; van Geert, 2011). The assumption of psychical homogeneity also contradicts fundamental design principles underlying all complex living systems in which different (non-isomorphic) structural elements are capable of performing or contributing to the same function, and vice versa, the same structures to different functions. That is, complex living systems feature both many-to-one structure–function relations (*degeneracy*, e.g., polygenic ‘traits') and one-to-many structure–function relations (*pluripotency*, e.g., pleiotropic ‘genes'; Mason, 2010, 2015). These unifying explanatory principles underlie the psychological concepts of *equifinality and multifinality*—individuals' capacities to leverage different psychical processes and structures to accomplish the same behavioural outcome, and vice versa (see Topic 1; Richters, 2021; Sato et al., 2009; Toomela, 2008b; Uher, 2022b, 2025).

When psychologists ignore their study phenomena's non-ergodicity in their statistical analysis, this entails fallible inferences as our next contribution shows. It highlights their implications for the interpretation of psychological findings and their replicability and presents an analytical method that allows for mitigating them.

### The ergodic fallacy: How psychology's erroneous ergodic assumptions can explain its inferential and reproducibility issues

Typical practice in psychological research is to aggregate data from many individuals to enable statistical analysis and to draw conclusions. In particular, the averages of scores of performances, or other psychological variables, are used to make inferences about the group of individuals studied—and even about the entire population from which it was sampled. These inferences are typically made in the form of generic statements about how “people” generally behave. These inferences are then used to make predictions about what single individuals might do in certain circumstances. Craig Speelman and Marek McGann articulated many problems with this chain of inferences, building on longstanding work across psychology's history.

#### Implicit assumptions of ergodicity entail fallible inferences from empirical findings, obscured by generically worded conclusions

Speelman and McGann (2013) highlighted several assumptions underlying the use of averages, which are often implicit and almost always problematic. Most vital is the idea that averaging removes noise in a data set to provide a ‘clearer' picture of some ‘true' value. Variance around the mean is supposed to originate from unimportant or possibly random factors that can be ‘averaged out' by focussing on the central tendency. This builds on the implicit assumption that the individuals in the group are all homogeneous with respect to the phenomena studied—thus, *ergodic*. In an ergodic system, all entities within the system are essentially interchangeable, such that knowledge of the entities' average scores can be used to predict the scores of any of these entities. But given that—for psychology's study phenomena—ergodicity cannot be assumed, the common practice of aggregating data over individuals is equivalent to trying to find the mean of apples, pears and bananas. The performance of each individual of a group rarely, if ever, matches the groups' average performance—indeed, psychological variables are often optimised for representing normal distribution patterns in a group.

The *ergodic fallacy*—the practice of erroneously assuming that sample-level findings could inform about individual-level phenomena (Fisher et al., 2018; Molenaar and Campbell, 2009; Richters, 2021; Rose, 2016; Speelman and McGann, 2020)—can lead to erroneous interpretations of statistical test results. For instance, group differences in performance scores are commonly taken to indicate that “people” in one condition performed better than those in another—as if the difference between the two group means reflects a difference present in all, or at least most, of the individuals in the groups studied.

These problems are obscured by the ambiguous wording often used in conclusions. Speelman et al. (2024) analysed a year of articles (*N* = 326) from three highly cited Q1 journals in the fields of cognitive, educational and clinical psychology. Over 88% of the papers reported generic conclusions about “people” or “participants” when interpreting findings derived from group-level analysis (e.g., null-hypothesis significance tests). Prevalence of this error was highest in papers from cognitive psychology (93.3%), which typically assess claims about ‘cognitive mechanisms' theorised as universal, compared to educational psychology (89.3%) and clinical psychology (77.9%), which are more concerned with individually relevant interventions. Still, prevalence of the ergodic fallacy was high in all fields.

#### How the ergodic fallacy may influence psychology's reproducibility problems: Pervasiveness analysis as a suitable alternative to aggregationist statistics

The ergodic fallacy provides a straightforward explanation for reproducibility problems in psychology (Speelman and McGann, 2020). Without assessing whether an effect is pervasive, or even widely prevalent, in a given sample, it is difficult to know what to expect from replication. If a set of scores represents, for example, the idiosyncratic combination of individuals' idiosyncratic behaviours, then any attempt to reproduce an effect with another sample of individuals will involve a different set of scores that, however, likewise represent idiosyncratic combinations of idiosyncratic behaviours (Tang and Braver, 2020). Given this, it is unsurprising that many effects are difficult to replicate in psychological research (Iso-Ahola, 2024; Mayrhofer et al., 2024).

As a simple alternative to aggregationist statistical analysis methods, Speelman and McGann (2020) described *pervasiveness analysis*. This technique involves counting the number of individuals who exhibited a particular behaviour. Reaching a benchmark of 80% in a sample is considered sufficient evidence to support generic statements, such as “most individuals showed this behaviour under these circumstances”. Moore et al. (2023) demonstrated the utility of this technique, by re-analysing the data of successful replications of nine famous psychology experiments, performed with null-hypothesis significance tests (Zwaan et al., 2018). Seven of these experiments met the pervasiveness criterion; that is, in each experiment, the target effect applied to over 80% of the participants. In the two other experiments, the classic effect applied to only 70% and 64% of the participants, respectively, although these experiments had passed the replicability criteria based on common significance tests.

Speelman and McGann's (2020) method for conducting a pervasiveness analysis is appropriate only for within-subjects designs. But pervasiveness analyses can also be applied to between-subject designs, correlational designs and forms of risk assessment. For these types of analyses, each set of findings is described in terms of “the number of persons who matched or failed to match expectation” (Grice et al., 2020, p.451) where the expectation is based on a theoretical prediction under test, such as more people given a drug will be classified as “cured” compared to people given a placebo. McManus et al. (2023, p. 2) extended this approach “to estimate the prevalence of person-level effects in the population” by comparing observed prevalence rates with null hypotheses of no effect. Interestingly, McManus and colleagues' re-analysis of existing data sets using this technique showed that previously reported statistically significant findings were often not associated with high pervasiveness values (also called prevalence values or Percent Correct Classification PCC indices). When surveying psychology researchers' knowledge of these problems, they also found that most researchers were largely ignorant of the potential dissociation between statistically significant effects and the pervasiveness of those effects in their samples.

Hence, pervasiveness analyses provide useful further insight into what is meant by an “effect” in a study and how many individuals of the sample actually met the desired criteria. They also showed how even successful replication studies can camouflage interesting and potentially important variation in (apparently) robust statistical outcomes. Importantly, though, pervasiveness analysis is unlikely to return a result of 100%—because of the non-ergodicity of human behaviour.

Pervasiveness analysis is an example of the epistemically justified analytical strategy that is necessary for generalising findings across unique individuals. Our next contribution elaborates on its methodological foundations and discusses suitable methodical approaches.

### Strategies for generalising findings across unique individuals in psychology: Misconceived nomotheticism and epistemically valid nomothetic approaches

As a science exploring individuals, psychology seems to contradict the old scientific dictum *scientia non est individuorum*^13^—the idea that scientific disciplines cannot be devoted to studying single cases given that science seeks regularities and lawfulness through abstraction and generalisation from particulars and unique events. Jana Uher explored the epistemological and methodological fundamentals that can be derived from this dictum in her line of research on individuals within and across not just different human cultures but also different species (e.g., Uher, 2011, 2013, 2015a,c,d, 2018b,c, 2022b).

#### Three strategies for generalising findings: Idiographic approaches, sample-based and case-by-case based nomothetic approaches

Windelband (1904/1998) categorised the sciences by their strategies of knowledge generation. Sciences of laws (e.g., physics, chemistry) study invariant relations of non-living matter (e.g., physical laws, chemical principles) using *nomothetic approaches* (from Greek *nomos*, the law). Sciences of events (e.g., history, sociology, political science), by contrast, study the ever-changing processes of human societies as they unfold through irreversible time using *idiographic approaches* (from Greek *idios*, the peculiar). Windelband's distinction reflects different strategies of knowledge generation that are aligned to the peculiarities of different objects of research. All sciences, however, apply both strategies—just to varied degrees because all research starts with a first case (Lamiell, 1998; Salvatore and Valsiner, 2010). Many sciences apply both strategies to equal extent. Evolutionary science, for example, studies unique events in the evolution of life (e.g., the dinosaurs' extinction) to derive general principles applicable to all species (e.g., adaptation, natural selection). Psychology, as well, studies unique individuals and aims to derive general principles that are applicable to many individuals. Thus, idiographic and nomothetic approaches are not mutually exclusive opposites, as often believed. Both are epistemically necessary and justified.

**Figure 5:**
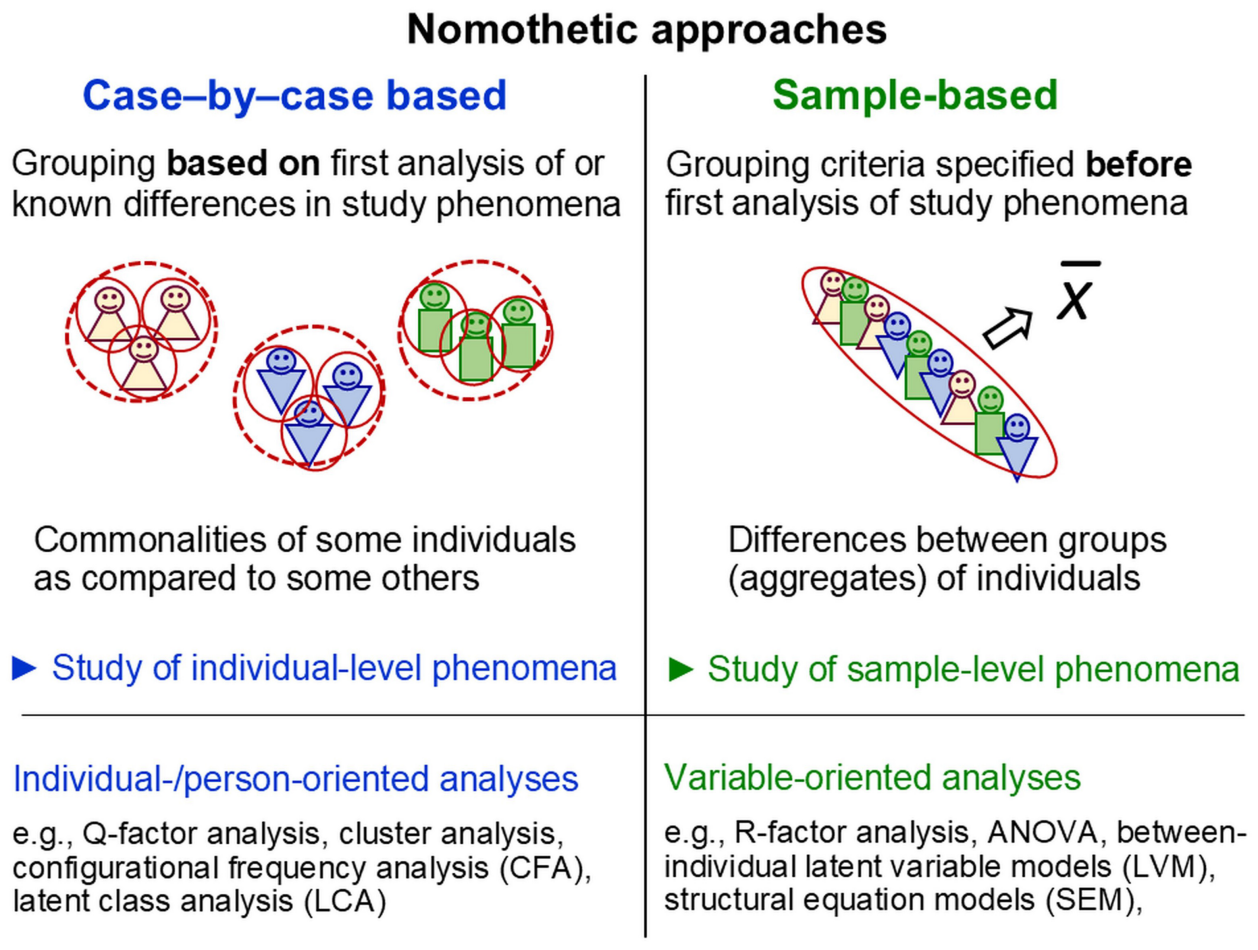
Two different strategies of nomothetic knowledge generation.

The physical sciences apply *sample-based nomothetic approaches* because (some of) their inanimate ergodic study systems feature synchronic and diachronic variations that are statistically isomorphic. Averages of many cases can therefore inform about every single case (e.g., electrons). To identify (‘lawful'—nomothetic) regularities and universal principles in psychology, quantitative psychologists (e.g., Francis Galton) adopted this approach analogously (Lamiell, 2003). The majority uses sample-level analyses and generalises their findings to the single individuals thus-summarised (Figure 5). That is, individuals are studied only as abstract examples of prototypical—yet inexistent—individuals (Allport, 1937; Danziger, 1985b, 1990; Robinson, 2011). Sample-based nomothetic approaches have turned psychology into a science that is largely studying groups and populations rather than individuals—thus, into *psycho-demography* (Lamiell, 2018; Smedslund, 2021).

This also seriously limits psychologists' possibilities for causal analyses. Indeed, to group individuals, researchers must specify criteria (encoded as ‘independent variables', e.g., gender, ethnicity) as possible causes of the phenomena analysed for between-group differences (e.g., intellectual abilities). These grouping criteria must be specified *a-priori—*thus, often before their relevance for a given research question is ascertained. For example, reviews of psychological meta-analyses showed that 78% of the effect sizes of reported gender differences were trivial or small (Cohen's *d* < 0.2; Hyde, 2005; Zell et al., 2015). Still, in the narrated interpretation, gender differences are often exaggerated, sometimes ‘supported' by statistical significance levels, although these are known to depend on sample size. Analysing differences between researcher-defined groups often fails to generate findings that are informative about individuals' functioning and development and possible causally relevant differences between them (Danziger, 1990; Lamiell, 2003; Richters, 2021; Smedslund, 2016; Uher, 2015c, 2022b; van Geert, 2011). This is because sample-level nomothetic approaches disconnect theory development from descriptions of real individuals and cannot reveal what is, indeed, common to all individuals in a group.

To appropriately consider the peculiarities of psychology's study phenomena (e.g., non-ergodicity, higher-order complexity), alternative nomothetic approaches are required—and possible. In *case-by-case based nomothetic approaches*, which can be traced back to Wilhelm Wundt already (Lamiell, 2003), individuals are grouped by the commonalities and differences that they are shown to exhibit in the study phenomena (Figure 5). Considering many-to-one (degeneracy, equifinality) and one-to-many (pluripotency, multifinality) structure–function relations, the individuals within each of the thus-created groups are then explored for further commonalities and differences. For example, rather than analysing gender or ethnicity differences as a default, groups of individuals may be formed who are scoring low, medium vs. high in ‘intelligence tests' to analyse what individuals within each group may have in common and what distinguishes them from those in the other groups, such as to identify possible factors promoting or hindering test performances. This nomothetic approach, because it is case-by-case based, allows researchers to identify generalities that are, indeed, common to all cases in a given group—a prerequisite for developing generalised knowledge and theories about intra-individual processes and functioning (Lamiell, 2003; Salvatore and Valsiner, 2010; Robinson, 2011; Uher, 2022b).

#### Individual-/person-oriented rather than variable-oriented analyses

Empirical implementations of the two different nomothetic strategies are based on Stern's (1911) methodological framework for exploring individuals and individual differences (Lamiell, 2003; Uher, 2011). It provides the necessary foundations for different, already well-established analytical methods to generalise findings across unique individuals.

Sample-based nomothetic approaches are empirically implemented through *variable-oriented analyses*, which explore the data matrix of *X*_*i*_ individuals by *Y*_*j*_ variables from the viewpoint of the *j* variables to study their value distributions across all *i* individuals. These methods analyse sample-level patterns in populations but not single individuals, such as using correlation or R factor analysis, ANOVA, between-individual latent variable models (LVMs) or structural equation models (SEM). Case-by-case based nomothetic approaches, by contrast, are empirically implemented through *individual-/person-oriented analyses*, which explore the data matrix from an orthogonal view and study the *i* individuals for their value distributions across all *j* variables. That is, these methods analyse *individual configurations* of values across different variables, which can be illustrated as a *profile* (e.g., ‘intelligence' profile). This profile reflects a property of the individual, but not of the population. Individual-/person-oriented analyses can also be used to identify groups of individuals sharing similar configurations—thus, (profile) *types*—such as using Q factor analysis, configurational frequency analysis (CFA), latent class analysis (LCA) or cluster analysis (Bergman and Andersson, 2010; Bergman and Lundh, 2015; Bergman and Trost, 2006; Bergman et al., 2017; Lundh, 2023, 2024; Uher, 2011; von Eye and Bogat, 2006).

Individual-/person-oriented analyses allow researchers to scrutinise the implications of data aggregation as well as the limitations and possibilities of making inferences from groups (on different levels of aggregation) to single individuals, and vice versa (von Eye and Bergman, 2003). These methodological approaches underlie Grice's (2011) Observation-Oriented Modelling (OOM), Barrett's actuary approaches (Grice et al., 2017b) and Speelman and McGann's (2020) pervasiveness analysis. Weber's (2021) concepts of property scope and property order, in turn, are essential to conceptualise the non-ergodicity of psychology's study phenomena on ontological levels. These approaches and concepts are indispensable for exploring what is, in fact, common to all individuals of a group as an important prerequisite for tackling psychology's crisis in generalisability, replicability and validity.

## Conclusions and future directions: Psychology can no longer ignore its Questionable Research Fundamentals (QRFs)

In this article, we demonstrated that the currently discussed Questionable Research Practices (QRPs) are just surface-level symptoms that obscure the root causes of psychology's crises—its Questionable Research Fundamentals (QRFs) of many of its established (and therefore no longer questioned) theories, concepts, approaches, methods and practices (Figure 1). Our compilation of critical perspectives on psychology's crises and current issues pinpoints four major areas of future development to advance psychology's research fundamentals.

**(1) The systematic elaboration of psychology's general philosophy of science, especially of ontologies, epistemologies and methodologies**

We discussed different philosophy-of-science perspectives underlying the approaches that we critically analysed as well as those that we presented, highlighting their specific presuppositions as well as crucial differences between them. Our aim was to show (a selection of) the diversity of philosophies and theories of science that are being used in quantitative psychology. But our analyses also revealed Questionable Research Fundamentals (QRFs) in the form of contradictions and incompatibilities inherent in some widely-used approaches (e.g., in psychometrics), which preclude epistemically justified inferences on the phenomena studied. These serious issues often go unnoticed, however, because many psychologists follow established theories, methods and practices without scrutinising their philosophy-of-science fundamentals. To develop epistemically justified approaches, it is crucial to make the philosophical presuppositions on which a given line of research is built explicit, and thus accessible to analysis and elaboration. This is a prerequisite to establish coherent paradigms in which the specific ontology, epistemology and methodology used in a given line of research—no matter which specific ones may be preferred—are systematically aligned to one another.

**(2) The advancement of the philosophy-of-science fundamentals of specific theories, approaches and methods that are appropriate for enabling quantitative research considering the peculiarities of psychology's study phenomena**

We demonstrated Questionable Research Fundamentals (QRFs) also underlying common theories and approaches of psychological ‘measurement' and pinpointed the challenges that must be mastered for establishing genuine analogues of measurement in psychology. To achieve this, quantitative psychologists must conceptualise how the peculiarities of its study phenomena (e.g., higher-order complexity, non-ergodicity) can be systematically connected to numerical (formal) models and known quantity standards. This also involves scrutinising the purported necessity and meaningfulness of quantitative investigations as well as the actual possibilities for implementing quantitative approaches and inevitable limitations.

**(3) The conceptual implementation of the epistemically necessary distinction between the phenomena under study and the means of their investigation**

Psychologists must heed the epistemic necessity to logically distinguish between the study phenomena (e.g., participants' beliefs, thoughts) and the means used for their exploration (e.g., methods, models) in a study in order to avoid conflating and thus confusing ontological with epistemological concepts (psychologists' cardinal error). This requires some basic knowledge about language and an increased awareness of its intricacies (e.g., inbuilt semantics). Such linguistic knowledge is necessary to explore and understand the challenges that these entail for psychological investigations, especially when using language-based methods (e.g., rating ‘scales', item variables).

**(4) The establishment of epistemically justified strategies for generalising findings across unique individuals**

We demonstrated that psychology's default use of sample-based nomothetic approaches to study individual-level phenomena, implemented through statistical variable-oriented analyses, builds on mathematical-statistical errors. It also ignores essential ontic peculiarities of its study phenomena, such as within-individual and between-individual variability, irreversible individual development and higher-order complexity (e.g., one-to-many and many-to-one relations, contextuality). These problems entail erroneous inferences from group-level findings to individual-level phenomena (e.g., ergodic fallacy), and vice versa, and also hinder causal analyses. To generalise across unique individuals, psychologists should capitalise on case-by-case based nomothetic approaches, implemented through individual-/person-oriented analyses for which the methodological fundamentals as well as suitable methods are already well established. These approaches are necessary to explore what some individuals do, in fact, have in common and what distinguishes them from others, which is prerequisite for unravelling (possibly) underlying structures and processes.

For each area of development, we presented various lines of research that, although established for years if not decades already, have still hardly been considered in mainstream psychology. With the increasing awareness of fundamental problems in psychological research and practice (e.g., psychology's crises), it is vital that more psychologists step out of their current comfort zone and start to actively and systematically advance the research fundamentals of psychological science. These novel directions can and should be built on the many fruitful developments that have already been made in psychology's history and diverse scientific communities. But these have been sidelined by the efficient mass production of purportedly ‘quantitative' data through rating ‘scales'. Their ease of use and efficiency enabled a blind *empiricism*—a focus on experience, largely disconnected from an elaborated body of theoretical knowledge—that fuelled the development of ever more sophisticated (and therefore impressive) statistical analyses—whereas psychology's actual study phenomena got out of focus.

### Just minimising Questionable Research Practices (QRPs) and using language-based algorithms will not remedy but only intensify psychology's crises

Mainstream psychologists launched large-scale initiatives (e.g., open science and replicability projects) to remedy questionable applications of established practices—thus, scientific misconduct. These approaches, however, encourage ever more empirical research—thus, mere empiricism—without elaborating the necessary theoretical and philosophical fundamentals. The novel technological possibilities provided by language-based algorithms (e.g., NLP algorithms, LLMs) allow for generating data sets even more rapidly than this has already been possible with the anonymous online surveys used in the last decades (Anderson et al., 2019)—and which are increasingly completed by online bots (Storozuk et al., 2020). The fascinating AI technologies have already generated an increasing volume of psychological research from artificially generated data to new ways of summarising findings. But this, in itself, will not address the serious issues underlying psychology's philosophies, theories and its language-based constructs and methods. Yet these novel technologies can be meaningfully applied to investigate how the inbuilt semantics of natural human languages mediate and shape individuals' thinking—including the theoretical thinking of scientists—and how individuals are relating their language to the real-world phenomena described.

### Psychology must tackle the Questionable Research Fundamentals (QRFs) of its established theories and practices and advance its philosophies of science

Tackling psychology's crises in replicability, generalisability, validity and confidence and the issues that cause and maintain them requires a rethinking of its established theories, methods and practices. Rather than trying to reinvent the wheel, mainstream psychology can and should capitalise on the advances already made over the last decades from different perspectives and fields of expertise. Therefore, we need more open and controversial yet constructive and collegial debates about our most basic presuppositions as well as honest and critical analyses of the possibilities and meaningfulness of quantification in psychology—*prioritising scientific integrity over expediency*. With our compilation of diverse perspectives on quantitative psychology's problems, we aim to set an example, to give new impetus to the current debates and to highlight important directions of future development that, as we believe, are necessary to rethink and advance psychology as a science.

## Footnotes

^1^Ontology and epistemology as well as their relation are variously defined. Still, the two should not be confounded. Assuming that both are interdependent, ontology can state about epistemology that concepts, theories, presumptions and beliefs are (scholars') psychical (e.g., mental) phenomena by their ontological nature. Hence, knowledge of a being is a state of being itself. Epistemology, in turn, can say about ontology that knowledge of the structure of beings is a kind of knowledge itself (Poli, 2001; Uher, 2023b).

^2^There are two schools of transdisciplinarity. The present analyses build on *theoretical transdisciplinarity*. *Applied (practical) transdisciplinarity*, by contrast, is aimed less at developing theoretical frameworks and new forms of knowledge but more at understanding real-world problems and developing tangible solutions. It involves scholars from different disciplines but also political, social and economic actors as well as ordinary citizens with the aim of producing socially robust knowledge rather than merely reliable scientific knowledge (Uher, 2024).

^3^For an English translation (see Michell and Ernst, 1996, 1997).

^4^The term ‘metaphysics' has a history of various meanings. Originally, it indicated only the order of Aristotle's works, in which it happened to be listed after those written on physics (Ancient Greek *meta* meant ‘after'). It is also often misinterpreted as denoting ‘what goes beyond physics or reality', linking it to speculation.

^5^Individual Differences and Assessment Network (IDANET); https://www.jiscmail.ac.uk/cgi-bin/webadmin?A0=idanet.

^6^In metrology, base units are conventionally defined entities (e.g., metre, second) that are used as references for quantitative physical properties that cannot be expressed in terms of other quantitative physical properties (e.g., length, time). Derived units, by contrast, are conventional references derived from combinations of some base units (e.g., volume from length, velocity from length and time; Uher, 2020a).

^7^For brevity, here “LVM” refers only to between-person LVMs.

^8^Rosen (1985; 1999) himself and others refer to this process model solely as *modelling relation*. To highlight that it involves the coherent modelling of four interrelated modelling relations (arrows 1 to 4) and to pinpoint key distinctions to the statistical modelling of data, which concerns solely arrow 3 in Rosen's general model, Uher (2025) refers to his process model as a *system of interrelated modelling relations*.

^9^Attentive readers will have noticed that the psychical—psychological (psyche—psychology) distinction is not made consistently in this paper, which reflects the differences in our linguistic and conceptual habits as authors (also given different language backgrounds).

^10^See our previous discussions on the notion of a “mind-independent reality”, which, if taken literally, can be misread as (e.g., the researcher's) mind being generally independent of, thus, extraneous to ‘reality' rather than forming part of it as well (p. 23).

^11^Questionnaires and Likert ‘scales' are used here to illustrate key concepts because psychologists are familiar with them. The preceding sections have already highlighted these methods' serious limitations as ‘measuring instruments', given their measurement theoretical, conceptual and methodological deficiencies as well as the intricacies that natural language entails for language-based methods (see Topics 2 and 3).

^12^*Formative indicators* are hypothesised to causally affect the latent construct that they underpin, whereas *reflective indicators* are hypothesised to be affected by the latent construct that underpins them. That is, the direction of causality between constructs and indicators differs between formative and reflective indicators (Bollen and Diamantopoulos, 2017).

^13^Latin, meaning “science is not about individual cases”.

## In memoriam

We dedicate this article to our dear colleagues and co-authors Paul Barrett and Robert J. Mislevy who died before they could see this article in print. Paul's tireless efforts to promote and argue for scientific integrity and a “think-for-yourself” attitude as well as Bob's integrative efforts for advancing the philosophy, theory and practice of educational assessment will be sorely missed.

## References

[B1] Abran A. Desharnais J.-M. Cuadrado-Gallego J. J. (2012). Measurement and quantification are not the same: ISO 15939 and ISO 9126. J. Softw. Evol. Process 24, 585–601. 10.1002/smr.496

[B2] Adam M. Hanna P. (2012). Your past is not their present: time, the other, and ethnocentrism in cross-cultural personality psychology. Theory Psychol. 22, 436–451. 10.1177/0959354311412107

[B3] AERA APA, and NCME. (2014). Standards for Educational and Psychological Testing. Washington, DC: American Educational Research Association.

[B4] Aeschliman M. D. (1998). The Restitution of Man: C. S. Lewis and the Case Against Scientism. Grand Rapids, MI: William B. Eerdmans Publishing Company.

[B5] Al-Ababneh M. (2020). Linking ontology, epistemology and research methodology. Sci. Philos. 8, 75–91. 10.23756/sp.v8i1.500

[B6] Ali M. (2023). “Research philosophies in social science and information systems research,” in Information Systems Research (Cham: Palgrave Macmillan, Cham), 256.

[B7] Allport G. W. (1937). Personality: A Psychological Interpretation. New York, NY: Holt, Rinehart & Winston.

[B8] Anderson C. A. Allen J. J. Plante C. Quigley-McBride A. Lovett A. Rokkum J. N. (2019). The MTurkification of social and personality psychology. Pers. Soc. Psychol. Bull. 45, 842–850. 10.1177/0146167218798821 30317918

[B9] Andrade C. (2021). HARKing, cherry-picking, p-hacking, fishing expeditions, and data dredging and mining as questionable research practices. J. Clin. Psychiatry 82:20f13804. 10.4088/JCP.20f13804 33999541

[B10] Arnulf J. K. Larsen K. R. (2020). Culture blind leadership research: how semantically determined survey data may fail to detect cultural differences. Front. Psychol. 11:176. 10.3389/fpsyg.2020.00176 32132948 PMC7040226

[B11] Arnulf J. K. Larsen K. R. (2021). “Semantic and ontological structures of psychological attributes,” in Measuring and Modeling Persons and Situations, eds. Wood D. Read S. J. Harms P. D. Slaughter A. (London: Academic Press), 69–102. 10.1016/B978-0-12-819200-9.00013-2

[B12] Arnulf J. K. Larsen K. R. Martinsen O. L. Bong C. H. (2014). Predicting survey responses: how and why semantics shape survey statistics on organizational behaviour. PLoS One 9:e106361. 10.1371/journal.pone.0106361 25184672 PMC4153608

[B13] Arnulf J. K. Larsen K. R. Martinsen O. L. Egeland T. (2018). The failing measurement of attitudes: How semantic determinants of individual survey responses come to replace measures of attitude strength. Behav. Res. Methods 50, 2345–2365. 10.3758/s13428-017-0999-y 29330764

[B14] Arnulf J. K. Larsen K. R. Martinsen Ø. L. Nimon K. F. (2021). Editorial: semantic algorithms in the assessment of attitudes and personality. Front. Psychol. 12:720559. 10.3389/fpsyg.2021.720559 34367039 PMC8342853

[B15] Arnulf J. K. Olsson U. H. Nimon K. (2024). Measuring the menu, not the food: “psychometric” data may instead measure “lingometrics” (and miss its greatest potential). Front. Psychol. 15:1308098. 10.3389/fpsyg.2024.1308098 38577112 PMC10991757

[B16] Bagozzi R. P. (2011). Measurement and meaning in information systems and organizational research: methodological and philosophical foundations. MIS Q. 35, 261–292. 10.2307/23044044

[B17] Bandura A. (1977). Self-efficacy: Toward a unifying theory of behavioral change. Psychol. Rev. 84:191. 10.1037/0033-295X.84.2.191 847061

[B18] Barrett P. T. (2003). Beyond psychometrics: measurement, non-quantitative structure, and applied numerics. J. Manag. Psychol. 18, 421–439. 10.1108/02683940310484026

[B19] Barrett P. T. (2005). What if there were no psychometrics?: constructs, complexity, and measurement. J. Pers. Assess. 85, 134–140. 10.1207/s15327752jpa8502_05 16171413

[B20] Barrett P. T. (2008). The consequence of sustaining a pathology: scientific stagnation—a commentary on the target article “Is psychometrics a pathological science?” by Joel Michell. Meas. Interdiscip. Res. Perspect. 6, 78–83. 10.1080/15366360802035521

[B21] Barrett P. T. (2011). Invoking arbitrary units is not a solution to the problem of quantification in the social sciences. Meas. Interdiscip. Res. Perspect. 9, 28–31. 10.1080/15366367.2011.558783

[B22] Barrett P. T. (2018). The EFPA test-review model: when good intentions meet a methodological thought disorder. Behav. Sci. 8, 1 5, 1–22. 10.3390/bs8010005 29403661 PMC5791023

[B23] Barrett P. T. (2024). The Cognadev AI-Series #1-4. Available online at: https://www.cognadev.com/blog_cat11.html (Accessed October 30, 2024).

[B24] Berglund B. (2012). “Measurement in psychology,” in Measurement with Persons: Theory, Methods, and Implementation Areas, eds. Berglund B. Rossi G. B. Townsend J. T. Pendrill L. (New York: Taylor Francis), 27–50.

[B25] Bergman L. R. Andersson H. (2010). The person and the variable in developmental psychology. J. Psychol. 218, 155–165. 10.1027/0044-3409/a000025

[B26] Bergman L. R. Lundh L.-G. (2015). The person-oriented approach: roots and roads to the future. J. Person-Oriented Res. 1, 1–109. 10.17505/jpor.2015.01

[B27] Bergman L. R. Trost K. (2006). The person-oriented versus the variable-oriented approach: are they complementary, opposites, or exploring different worlds? Merrill-Palmer Q. 52, 601–632. 10.1353/mpq.2006.002334409987

[B28] Bergman L. R. Vargha A. Kövi Z. (2017). Revitalizing the typological approach: some methods for finding types. J.Person Orient. Res. 3, 49–62. 10.17505/jpor.2017.04 33569123 PMC7869617

[B29] Bernstein J. H. (2015). Transdisciplinarity: a review of its origins, development, and current issues. J. Res. Pract. 11:412. Available online at: https://jrp.icaap.org/index.php/jrp/article/view/510.html (Accessed September 23, 2019).36851146

[B30] Bevir M. Blakely J. (2018). Interpretive Social Science: An Anti-Naturalist Approach. Oxford: Oxford University Press.

[B31] Bhaskar R. Danermark B. (2006). Metatheory, interdisciplinarity and disability research: a critical realist perspective. Scand. J. Disabil. Res. 8, 278–297. 10.1080/15017410600914329

[B32] Bickhard M. H. (2001). The tragedy of operationalism. Theory Psychol. 11, 35–44. 10.1177/0959354301111002

[B33] Bird A. (2022). “Thomas Kuhn”, in The Stanford Encyclopedia of Philosophy, ed. Edward N. Zalta. Available online at: https://plato.stanford.edu/archives/spr2022/entries/thomas-kuhn (Accessed May 23, 2025).

[B34] Birkhoff G. D. (1931). Proof of the ergodic theorem. Proc. Natl. Acad. Sci. U.S.A. 17, 656–660. 10.1073/pnas.17.2.656 16577406 PMC1076138

[B35] Bollen K. A. Diamantopoulos A. (2017). In defense of causal-formative indicators: a minority report. Psychol. Methods 22, 581–596. 10.1037/met0000056 26390170 PMC6670294

[B36] Boroditsky L. (2018). 7,000 universes: how the languages we speak shape the ways we think. Toronto, ON: Doubleday Canada.

[B37] Borsboom D. (2005). Measuring the Mind: Conceptual Issues in Contemporary Psychometrics. Cambridge: Cambridge University Press.

[B38] Borsboom D. (2006). The attack of the psychometricians. Psychometrika 71, 425–440. 10.1007/s11336-006-1447-6 19946599 PMC2779444

[B39] Borsboom D. (2008). Latent variable theory. Meas. Interdiscip. Res. Perspect. 6, 25–53. 10.1080/15366360802035497

[B40] Borsboom D. Mellenbergh G. J. (2004). Why psychometrics is not pathological. Theory Psychol. 14, 105–120. 10.1177/0959354304040200

[B41] Borsboom D. Scholten A. Z. (2008). The Rasch model and conjoint measurement theory from the perspective of psychometrics. Theory Psychol. 18, 111–117. 10.1177/0959354307086925

[B42] Bridgman P. W. (1927). The Logic of Modern Physics. New York: Macmillan.

[B43] Bridgman P. W. (1938). Operational analysis. Philos. Sci. 5, 114–131. 10.1086/286496

[B44] Bruner J. (1990). Acts of Meaning. Cambridge: Harvard University Press.

[B45] Bunge M. (1977). Treatise on Basic Philosophy: Ontology I: The Furniture of the World, Vol. 3. Dordrecht, Boston: D. Reidel Publishing.

[B46] Bunge M. (1981). “A materialist theory of mind,” in Scientific Materialism. Episteme, Vol. 9 (Dordrecht: Springer), 67–89.

[B47] Bunge M. (1993). Realism and antirealism in social science. Theory Decis. 35, 207–235. 10.1007/BF0107519910732877

[B48] Bunge M. A. (1974). Treatise on Basic Philosophy Volume 1: Semantics I – Sense and Reference. Dordrecht, The Netherlands: D. Reidel Publishing Company.

[B49] Bunge M. A. (1975). What is a quality of life indicator? Soc. Indic. Res. 2, 65–79. 10.1007/BF00300471

[B50] Bunge M. A. (1979). Treatise on Basic Philosophy Volume 4: Ontology II - A World of Systems. Dordrecht, The Netherlands: Kluwer Academic Publishers.

[B51] Bunge M. A. (2003). Philosophical Dictionary (Enlarged ed.). Amherst, New York: Promenteus Books.

[B52] Bunge M. A. (2010). Reading measuring instruments. Spont. Generations 4, 85–93. 10.4245/sponge.v4i1.11725

[B53] Byrne D. (2002). Interpreting Quantitative Data. London: Sage Publications.

[B54] Campbell D. T. (1974). “Qualitative knowing in action research. Kurt Lewin award address,” in Society for the Psychological Study of Social Issues, Presented at the Meeting of the American Psychological Association (New Orleans, LA: APA).

[B55] Chakravartty A. (2017). “Scientific realism”, in The Stanford Encyclopedia of Philosophy (Summer 2017 Edition), ed. Edward N. Zalta. Available online at: https://plato.stanford.edu/archives/sum2017/entries/scientific-realism (Accessed May 13, 2025).

[B56] Chang H. (1995). Circularity and reliability in measurement. Perspect. Sci. 3, 153–172. 10.1162/posc_a_00479

[B57] Chang H. (2004). Inventing Temperature: Measurement and Scientific Progress. Oxford: Oxford University Press.

[B58] Chang Y. Wang X. Wang J. Wu Y. Yang L. Zhu K.. (2024). A survey on evaluation of large language models. ACM Trans. Intell. Syst. Technol. 15:39. 10.1145/3641289

[B59] Cicourel A. V. (1964). Method and Measurement in Sociology. New York, NY: Free Press of Glencoe.

[B60] Collingwood R. G. (1940). An Essay on Metaphysics. Oxford: Oxford University Press.

[B61] Cordero A. (2012). Mario Bunge's scientific realism. Sci. Educ. 21, 1419–1435. 10.1007/s11191-012-9456-6

[B62] Cornejo C. Valsiner J. (2021). “Mathematical thinking, social practices, and the locus of science in psychology,” in A Pragmatic Perspective of Measurement (S. vii–xi), eds. Torres Irribarra D. (Cham: Springer International Publishing).

[B63] Danziger K. (1985a). The origins of the psychological experiment as a social institution. Am. Psychol. 40, 133–140. 10.1037//0003-066X.40.2.133

[B64] Danziger K. (1985b). The methodological imperative in psychology. Philos. Soc. Sci. 15, 1–13. 10.1177/004839318501500101

[B65] Danziger K. (1990). Constructing the Subject: Historical Origins of Psychological Research. New York, NY: Cambridge University Press.

[B66] Danziger K. Dzinas K. (1997). How psychology got its variables. Can. Psychol. 38, 43–48. 10.1037/0708-5591.38.1.43

[B67] Daston L. Galison P. (2007). Objectivity. New York, NY: Zone Books.

[B68] de Lubac H. (1995). The Drama of Atheist Humanism. San Francisco, CA: Ignatius Press.

[B69] Deutscher G. (2006). The Unfolding of Language: The Evolution of Mankind's Greatest Invention. London, UK: Arrow.

[B70] Deutscher G. (2010). Through the language glass: Why the World Looks Different in Other Languages. New York, NY: Metropolitan Books/Henry Holt and Company.

[B71] Devlin J. Chang M.-W. Lee K. Toutanova K. (2018). Bert: pre-training of deep bidirectional transformers for language understanding. arXiv [Preprint]. *arXiv:*1810.04805. 10.48550/arXiv.1810.04805

[B72] Diez Roux A. V. (2002). A glossary for multilevel analysis. J. Epidemiol. Commun. Health 56, 588–594. 10.1136/jech.56.8.588 12118049 PMC1732212

[B73] Durkheim E. (1893/1984). The Division of Labor in Society (W. D. Halls, Trans.). New York, NY: Free Press.

[B74] Earp B. D. Trafimow D. (2015). Replication, falsification, and the crisis of confidence in social psychology. Front. Psychol. 6:621. 10.3389/fpsyg.2015.00621 26042061 PMC4436798

[B75] Edwards J. R. (2011). The fallacy of formative measurement. Organ. Res. Methods 14, 370–388. 10.1177/1094428110378369

[B76] Elson M. Hussey I. Alsalti T. Arslan R. C. (2023). Psychological measures aren't toothbrushes. Commun. Psychol. 1, 1–4. 10.1038/s44271-023-00026-9 39242966 PMC11332227

[B77] Eronen M. I. (2024). Causal complexity and psychological measurement. Philos. Psychol. 1–16. 10.1080/09515089.2023.2300693

[B78] Fahrenberg J. (2013). Zur Kategorienlehre der Psychologie: Komplementaritätsprinzip; Perspektiven und Perspektiven-Wechsel [On Category Systems in Psychology. Complementarity Principle. Perspectivism and Perspective-taking]. Lengerich, Germany: Pabst Science Publishers.

[B79] Fahrenberg J. (2015). Theoretische Psychologie – Eine Systematik der Kontroversen. [Theoretical psychology – A systematization of controversies]. Lengerich, Germany: Pabst Science Publishers.

[B80] Faucheux C. (1976). Cross-cultural research in experimental social psychology. Eur. J. Soc. Psychol. 6, 269–322. 10.1002/ejsp.2420060302

[B81] Faust D. (2012). Ziskin's Coping with Psychiatric and Psychological Testimony. New York: Oxford University Press.

[B82] Fechner G. T. (1858). Das Psychische Maß. Z. Philos. Kritik 32, 1–24.26135273

[B83] Fechner G. T. (1860a). Elemente der Psychophysik I (Vol. 1). Leipzig: Breitkopf und Härtel.

[B84] Fechner G. T. (1860b). Elemente der Psychophysik II (Vol. 2). Leipzig: Breitkopf und Härtel.

[B85] Feest U. (2005). Operationism in psychology: what the debate is about, what the debate should be about. J. Hist. Behav. Sci. 41, 131–149. 10.1002/jhbs.20079 15812819

[B86] Ferguson A. Myers C. S. Bartlett R. J. Banister H. Bartlett F. C. Brown W.. (1940). Quantitative estimates of sensory events: final report of the committee appointed to consider and report upon the possibility of quantitative estimates of sensory events. Adv. Sci. 1, 331–349.

[B87] Finkelstein L. (2003). Widely, strongly and weakly defined measurement. Measurement 34, 39–48. 10.1016/S0263-2241(03)00018-6

[B88] Fisher A. J. Medaglia J. D. Jeronimus B. F. (2018). Lack of group-to-individual generalizability is a threat to human subjects research. Proc. Natl. Acad. Sci. USA. 115, E6106–E6115. 10.1073/pnas.1711978115 29915059 PMC6142277

[B89] Fleck L. (1935/1979). Genesis and development of a scientific fact [Translation of: Entstehung und Entwicklung einer wissenschaftlichen Tatsache, original 1935]. Chicago: University of Chicago Press.

[B90] Flynn J. R. (2012). Are We Getting Smarter? Rising IQ in the Twenty-First Century. Cambridge: Cambridge University Press. 10.1017/CBO9781139235679

[B91] Freeman M. (2024). Toward the Psychological Humanities. A Modest Manifesto for the Future of Psychology. London: Routledge.

[B92] Frigg R. Nguyen J. (2021). “Scientific representation,” in The Stanford Encyclopedia of Philosophy, ed. Zalta E. (Cambridge: Cambridge University Press). Available online at: https://plato.stanford.edu/archives/win2021/entries/scientific-representation (Accessed March 31, 2025).

[B93] Gee J. P. (2008). “Learning theory, video games, and popular culture,” in The International Handbook of Children, Media, and Culture, eds. Drotner K. Livingstone S. (London: Sage Publications), 196–211.

[B94] Gee J. P. (2021). “Thinking, learning, and reading: the situated social mind,” in Situated Cognition: Social, Semiotic, and Psychological Perspectives, eds. Kirshner D. Whitson (New York: Routledge), 235–259.

[B95] Gergen K. J. (1973). Social psychology as history. J. Pers. Soc. Psychol. 26, 309–320. 10.1037/h0034436

[B96] Gergen K. J. (2001). Psychological science in a postmodern context. Am. Psychol. 56, 803–813. 10.1037/0003-066X.56.10.803 11675987

[B97] Gibbs P. Beavis A. (2020). Contemporary Thinking on Transdisciplinary Knowledge: What Those Who Know, Know. Cham, Switzerland: Springer.

[B98] Gnatt E. E. (2018). “Scientism and saturation: evolutionary psychology, human experience, and the phenomenology of Jean-Luc Marion,” in On Hijacking Science. Exploring the Nature of Consequences of Overreach in Pychology, eds. Gnatt E. E. Williams R. N. (New York, NY: Routledge), 52–67.

[B99] Gong T. Shuai L. Mislevy R. J. (2023). Sociocognitive processes and item response models: a didactic example. J. Educ. Meas. 61, 150–173. 10.1111/jedm.12376

[B100] Gould S. J. (1996). The Mismeasure of Man (extended and revised ed.). New York: W.W. Norton.

[B101] Gray R. M. (1988). “Ergodic theorems,” in Probability, Random Processes, and Ergodic Properties (New York, NY: Springer), 216–243.

[B102] Green C. D. (2001). Operationism again: what did Bridgman say? What did Bridgman need? Theory Psychol. 11, 45–51. 10.1177/0959354301111003

[B103] Grice J. (2011). Observation Oriented Modeling: Analysis of Cause in the Behavioral Sciences. New York: Academic Press.

[B104] Grice J. Barrett P. Cota L. Felix C. Taylor Z. Garner S.. (2017b). Four bad habits of modern psychologists. Behav. Sci. 7:53. 10.3390/bs7030053 28805739 PMC5618061

[B105] Grice J. Barrett P. Schlimgen Abramson, C. (2012). Toward a brighter future for psychology as an observa/on oriented science. Behav. Sci. 2, 1–22. 10.3390/bs2010001 25379212 PMC4217578

[B106] Grice J. W. Medellin E. Jones I. Horvath S. McDaniel H. O'lansen C.. (2020). Persons as effect sizes. Adv. Methods Pract. Psychol. Sci. 3, 443–455. 10.1177/2515245920922982

[B107] Grice J. W. Yepez M. Wilson N. L. Shoda Y. (2017a). Observation-oriented modeling: going beyond “is it all a matter of chance”? Educ. Psychol. Meas. 77, 855–867. 10.1177/0013164416667985 29795935 PMC5965635

[B108] Guyon H. (2018). The fallacy of the theoretical meaning of formative constructs. Front. Psychol. 9:179. 10.3389/fpsyg.2018.00179 29497396 PMC5819317

[B109] Hacking I. (1990). The Taming of Chance. New York: Cambridge University Press.

[B110] Hacking I. (2002). Historical Ontology. Cambridge, MA: Harvard University Press.

[B111] Hammack P. L. Josselson R. (2021). Essentials of Narrative Analysis. Washington, DC: American Psychological Association.

[B112] Hanfstingl B. Oberleiter S. Pietschnig J. Tran U. S. Voracek M. (2024). Detecting jingle and jangle fallacies by identifying consistencies and variabilities in study specifications – a call for research. Front. Psychol. 15:1404060. 10.3389/fpsyg.2024.1404060 39282677 PMC11393684

[B113] Hardin A. M. Marcoulides G. A. (2011). A commentary on the use of formative measurement. Educ. Psychol. Meas. 71, 753–764. 10.1177/0013164411414270

[B114] Harré R. Van Langenhove L. (1999). Positioning Theory: Moral Contexts of Intentional Action. Oxford: Blackwell.

[B115] Harris C. J. Krajcik J. S. Pellegrino J. W. McElhaney K. W. (2016). Constructing Assessment Tasks that Blend Disciplinary Core Ideas, Crosscutting Concepts, and Science Practices for Classroom Formative Applications. Menlo Park, CA: SRI International.

[B116] Hartmann N. (1964). Der Aufbau der realen welt. Grundriss der allgemeinen Kategorienlehre [the Structure of the Real World. Outline of the General Theory of Categories], 3rd Edn. Berlin: Walter de Gruyter.

[B117] Harvard S. Winsberg E. (2022). The epistemic risk in representation. Kennedy Inst. Ethics J. 31, 1–31. 10.1353/ken.2022.0001 35431275

[B118] Heene M. (2011). An old problem with a new solution, raising classical questions: a commentary on Humphry. Measurement 9, 51–54. 10.1080/15366367.2011.558790

[B119] Heine J. H. Heene M. (2025). Measurement and mind: unveiling the self-delusion of metrification in psychology. Meas. Interdiscip. Res. Perspect. 23, 213–241. 10.1080/15366367.2024.2329958

[B120] Heisenberg W. (1927). Über den anschaulichen Inhalt der quantentheoretischen Kinematik und Mechanik [On The Actual Content of Quantum Theoretical Kinematics and Mechanics]. Zeit. Phys. 43, 172–198. 10.1007/BF01397280

[B121] Hempel C. G. (1952). “Fundamentals of concept formation in empirical science,” in International Encyclopedia of Unified Science, Vol. 2, ed. Gustav H. C. (Chicago: University of Chicago Press). Available online at: https://archive.org/details/fundamentalsofco0000hemp (Accessed November 28, 2024).

[B122] Hibberd F. J. (2019). What is scientific definition? J. Mind Behav. 40, 29–52. Available online at: https://www.jstor.org/stable/26740746

[B123] Hölder O. (1901). Die Axiome der Quantität und die Lehre vom Mass. Berichte über die Verhandlungen der Königlich Sächsischen Gesellschaft der Wissenschaften zu Leipzig, Mathematisch-Physische Classe 53, 1–64.

[B124] Holzkamp K. (1983). Grundlegung der Psychologie. Frankfurt/M.: Campus.

[B125] Holzkamp K. (2013). “Missing the point: variable psychology's blindness to the problem's inherent coherences,” in Psychology from the standpoint of the subject: Selected writings of Klaus Holzkamp, eds. Schraube E. Osterkamp U. (London: Palgrave Macmillan), 60–74.

[B126] Howell K. E. (2013). An Introduction to the Philosophy of Methodology. London: SAGE Publications Ltd.

[B127] Hyde J. S. (2005). The gender similarities hypothesis. Am. Psychol. 60, 581–592. 10.1037/0003-066X.60.6.581 16173891

[B128] Ichheiser G. (1943). Why psychologists tend to overlook certain “obvious” facts. Philos. Sci. 10, 204–207. 10.1086/286811

[B129] Iso-Ahola S. E. (2024). Science of psychological phenomena and their testing. Am. Psychol. 10.1037/amp0001362. [Epub ahead of print]. 38709630

[B130] James W. (1895). The knowing of things together. Psychol. Rev. 2, 105–124. 10.1037/h0073221

[B131] John L. K. Loewenstein G. Prelec D. (2012). Measuring the prevalence of questionable research practices with incentives for truth telling. Psychol. Sci. 23, 524–532. 10.1177/0956797611430953 22508865

[B132] Jovanović G. (2022). “Epistemology of psychology,” in International Handbook of Psychology Learning and Teaching, Springer International Handbooks of Education, eds. Zumbach J. Bernstein D Narciss S. Marsico G. (Cham, Switzerland: Springer, Cham).

[B133] Kane M. T. (1992). An argument-based approach to validity. Psychol. Bull. 112, 527–535. 10.1037/0033-2909.112.3.527

[B134] Kaplan A. (1964). The Conduct of Inquiry: Methodology for Behavioral Science. Scranton, PA: Chandler Publishing Co.

[B135] Kelley T. L. (1927). Interpretation of Educational Measurements. Yonkers, NY: World.

[B136] Kelly G. (1955). The Psychology of Personal Constructs (Volume 1 and 2). London, UK: Routledge.

[B137] Kelly G. (1963). A Theory of Personality: The Psychology of Personal Constructs. London: W.W. Norton.

[B138] Kelso J. A. S. (1995). Dynamic Patterns: The Self-Organization of Brain and Behavior (Complex adaptive systems). Cambridge, Mass: The MIT Press.

[B139] Kirschenbaum H. (2007). The Life and Work of Carl Rogers. Alexandria, VA: PCCS Books.

[B140] Kirschner S. R. Martin J. (2010). The Sociocultural Turn in psychology: The Contextual Emergence of Mind and Self. New York: Columbia University Press.

[B141] Klima G,. (2022). “The medieval problem of universals”, in The Stanford Encyclopedia of Philosophy (Spring 2022 Edition), ed. Edward N. Zalta. Available online at: https://plato.stanford.edu/archives/spr2022/entries/universals-medieval (Accessed May 14, 2025).

[B142] Koch S. (1992). Psychology's Bridgman vs Bridgman's Bridgman: an essay in reconstruction. Theory Psychol. 2, 261–290. 10.1177/0959354392023002

[B143] Koffka K. (1935). Principles of Gestalt Psychology. London: Routledge and Kegan Paul.

[B144] Krantz D. Luce R. D. Tversky A. Suppes P. (1971). Foundations of Measurement Vol. I: Additive and Polynomial Representations. San Diego: Academic Press.

[B145] Kuhn T. (1962/1970). The Structure of Scientific Revolutions. Chicago: University of Chicago Press (1970, 2nd edition, with postscript).

[B146] Lamiell J. T. (1998). ‘Nomothetic' and ‘Idiographic': contrasting Windelband's understanding with contemporary usage. Theory Psychol. 8, 23–38. 10.1177/0959354398081002

[B147] Lamiell J. T. (2003). Beyond Individual and Group Differences: Human Individuality, Scientific Psychology, and William Stern's Critical Personalism. Thousand Oaks, CA: Sage Publications.

[B148] Lamiell J. T. (2013). Statisticism in personality psychologists' use of trait constructs: what is it? How was it contracted? Is there a cure? New Ideas Psychol. 31, 65–71. 10.1016/j.newideapsych.2011.02.009

[B149] Lamiell J. T. (2018). “On scientism in psychology: some observations of historical relevance,” in On Hijacking Science. Exploring the Nature of Consequences of Overreach in Psychology, eds. Gnatt E. E. Williams R. N. (New York, NY: Routledge), 27–41.

[B150] Lamiell J. T. (2019a). Psychology's Misuse of Statistics and Persistent Dismissal of its Critics. Cham: Springer International. 10.1007/978-3-030-12131-0

[B151] Lamiell J. T. (2019b). Re-centering psychology: from variables and statistics to persons and their stories. Theory Psychol. 29, 282–284. 10.1177/0959354318766714

[B152] Landauer T. K. Dumais S. T. (1997). A solution to Plato's problem: the latent semantic analysis theory of acquisition, induction, and representation of knowledge. Psychol. Rev. 104, 211–240. 10.1037/0033-295X.104.2.211

[B153] Larsen K. R. Voronovich Z. A. Cook P. F. Pedro L. W. (2013). Addicted to constructs: Science in reverse? Addiction 108, 1532–1533. 10.1111/add.12227 23718564

[B154] Legg C. Hookway C. (2024). “Pragmatism”, in The Stanford Encyclopedia of Philosophy, eds. Zalta E. N. Nodelman U.. Available online at: https://plato.stanford.edu/archives/win2024/entries/pragmatism (Accessed June 03,2025).

[B155] Lewin K. (1936). Principles of Topological Psychology. New York, NY: McGraw-Hill.

[B156] Likert R. (1932). A technique for the measurement of attitudes. Arch. Psychol. 22, 1–55.

[B157] Linkov V. (2024). Qualitative (pure) mathematics as an alternative to measurement. Front. Psychol. 15:1374308. 10.3389/fpsyg.2024.1374308 39417021 PMC11479882

[B158] Lord F. M. (2012). Applications of Item Response Theory to Practical Testing Problems. New York, NY: Routledge.

[B159] Lovasz N. Slaney K. L. (2013). What makes a hypothetical construct “hypothetical”? Tracing the origins and uses of the ‘hypothetical construct' concept in psychological science. New Ideas Psychol. 31, 22–31. 10.1016/j.newideapsych.2011.02.005

[B160] Luce R. D. Krantz D. Suppes P. Tversky A. (1990). Foundations of Measurement, Vol. 3: Representation, Axiomatization, and Invariance. San Diego, CA: Academic Press.

[B161] Luchetti M. (2020). From successful measurement to the birth of a law: disentangling coordination in Ohm's scientific practice. Stud. Hist. Philos. Sci. Part A 84, 119–131. 10.1016/j.shpsa.2020.09.005 33218458

[B162] Luchetti M. (2024). Epistemic circularity and measurement validity in quantitative psychology: insights from Fechner's psychophysics. Front. Psychol. 15:1354392. 10.3389/fpsyg.2024.1354392 38840738 PMC11151747

[B163] Lundh L. G. (2023). Person, population, mechanism. Three main branches of psychological science. J. Person Orient. Res. 9, 75–92. 10.17505/jpor.2023.25814 38107200 PMC10722369

[B164] Lundh L. G. (2024). Person, population, mechanism. A rejoinder to critics and an elaboration of the three-branch model. J. Person Orient. Res. 10, 68–84. 10.17505/jpor.2024.26295 38841559 PMC11149408

[B165] Luria A. R. (1973). The Working Brain. An Introduction to Neuropsychology. New York: Basic Books.

[B166] MacKenzie S. B. Podsakoff P. M. Podsakoff N. P. (2011). Construct measurement and validation procedures in MIS and behavioral research: Integrating new and existing techniques. MIS Q. 35, 293–334. 10.2307/23044045

[B167] Madsen O. J. (2014). The Therapeutic Turn: How Psychology Altered Western Culture. Hove, East Sussex: Routledge.

[B168] Mahner M. (2021). Mario Bunge (1919–2020): conjoining philosophy of science and scientific philosophy. J. Gen. Philos. Sci. 52, 3–23. 10.1007/s10838-021-09553-7

[B169] Maraun M. D. (1998). Measurement as a normative practice: implications of Wittgenstein's philosophy for measurement in psychology. Theory Psychol. 8, 435–461. 10.1177/0959354398084001

[B170] Maraun M. D. Gabriel S. M. (2013). Illegitimate concept equating in the partial fusion of construct validation theory and latent variable modeling. New Ideas Psychol. 31, 32–42. 10.1016/j.newideapsych.2011.02.006

[B171] Maraun M. D. Halpin P. F. (2008). Manifest and latent variates. Measurement 6, 113–117. 10.1080/15366360802035596

[B172] Margenau H. (1950). The Nature of Physical Reality. A Philosophy of Modern Physics. New York: McGraw-Hill.

[B173] Mari L. Carbone P. Giordani A. Petri D. (2017). A structural interpretation of measurement and some related epistemological issues. Stud. Hist. Philos. Sci. 65–66, 46–56. 10.1016/j.shpsa.2017.08.001 29195648

[B174] Mari L. Carbone P. Petri D. (2015). “Fundamentals of hard and soft measurement,” in Modern Measurements: Fundamentals and Applications, eds. Ferrero A. Petri D. Carbone P. Catelani M. (Hoboken, NJ: John Wiley and Sons), 203–262.

[B175] Mari L. Maul A. Irribarra D. T. Wilson M. (2013). Quantification is neither necessary nor sufficient for measurement. J. Phys. Conf. Ser. 459:012007. 10.1088/1742-6596/459/1/012007

[B176] Mari L. Wilson M. Maul A. (2021). “Measurement across the sciences,” in Developing a Shared Concept System For Measurement (Cham: Springer). 10.1007/978-3-030-65558-7

[B177] Markus K. A. (2021). Philosophical methodology and axiomatic measurement theory: a comment on Uher (2021). J. Theor. Philos. Psychol. 41, 85–90. 10.1037/teo0000178

[B178] Martin J. (2013). Life positioning analysis: an analytic framework for the study of lives and life narratives. J. Theor. Philos. Psychol. 33, 1–17. 10.1037/a0025916

[B179] Martin J. (2017). Carl Rogers' and B. F. Skinner's approaches to personal and societal improvement: a study in the psychological humanities. J. Theor. Philos. Psychol. 37, 214–229. 10.1037/teo0000072

[B180] Martin J. (2022). “A non-reductive “person-based ontology” for psychological inquiry,” in Routledge International Handbook of Theoretical and Philosophical Psychology, eds. B. D. Slife, S. C. Yanchar, and F. C. Richardson (New York, NY: Routledge), 391–411.

[B181] Martin J. (2024). Studies of Life Positioning: A New Sociocultural Approach to Psychobiography. New York, NY: Routledge.

[B182] Martin J. Bickhard M. H. (Eds.) (2013). The Psychology of Personhood: Philosophical, Historical, Socio-Developmental, and Narrative Approaches. New York: Columbia University Press.

[B183] Martin J. McLellan A. (2013). The Education of Selves: How Psychology Transformed Students. New York: Oxford University Press.

[B184] Martin J. Sugarman J. (1999). The Psychology of Human Possibility and Constraint. Albany, NY: State University of New York Press.

[B185] Martin J. Sugarman J. (2009). Does interpretation in psychology differ from interpretation in natural science? J. Theory Soc. Behav. 39, 19–37. 10.1111/j.1468-5914.2008.00394.x

[B186] Martin J. Sugarman J. Hickinbottom S. (2010). Persons: Understanding Psychological Selfhood and Agency. New York: Springer.

[B187] Martin J. Sugarman J. Slaney K. L. (Eds.) (2015). Wiley Handbook of Theoretical and Philosophical Psychology: Methods, Approaches and New Directions for Social Science. Wiley Blackwell.

[B188] Martin J. Sugarman J. Thompson J. (2003). Psychology and the Question of Agency. Albany: State University of New York Press.

[B189] Maslow A. H. (1966). The Psychology of Science. A reconnaissance. New York: Gateway.

[B190] Mason P. H. (2010). Degeneracy at multiple levels of complexity. Biol. Theory 5, 277–288. 10.1162/BIOT_a_00041

[B191] Mason P. H. (2015). Degeneracy: demystifying and destigmatizing a core concept in systems biology. Complexity 20, 12–21. 10.1002/cplx.21534

[B192] Matthews M. R. (Ed.). (2019). Mario Bunge: A Centenary Festschrift. Cham: Springer.

[B193] Mayrhofer R. Büchner I. C. Hevesi J. (2024). The quantitative paradigm and the nature of the human mind. The replication crisis as an epistemological crisis of quantitative psychology in view of the ontic nature of the psyche. Front. Psychol. 15:1390233. 10.3389/fpsyg.2024.1390233 39328812 PMC11424412

[B194] Mazur L. B. (2015). Defining power in social psychology. Orbis Idearum 2, 101–114. 10.26106/0D0Q-H908

[B195] Mazur L. B. (2017). “Gaps in human knowledge: highlighting the whole beyond our conceptual reach,” in The Psychology of Imagination: History, Theory and New Research Horizons, eds. B. Wagoner, I. Bresco, and S. H. Awad (Charlotte: Information Age Publishing), 239–252.

[B196] Mazur L. B. (2021). The epistemological imperialism of science. Reinvigorating early critiques of scientism. Front. Psychol. 11:609823. 10.3389/fpsyg.2020.609823 33488476 PMC7817851

[B197] Mazur L. B. (2022). “Experimentation within the social identity approach. History, highlights, and hurdles,” in Cambridge Handbook of Identity, eds. M. Bamberg, C. Demuth, and M. Watzlawik (Cambridge: Cambridge University Press), 435–459.

[B198] Mazur L. B. (2024a). “A dim recognition.” Religion as a font of psychological innovation. Integr. Psychol. Behav. Sci. 58, 845–854. 10.1007/s12124-024-09858-4 39039333 PMC11300530

[B199] Mazur L. B. (2024b). The desire for power within activist burnout. An illustration of the value of interpretive social science. Sociol. Compass 18:e13186. 10.1111/soc4.13186

[B200] Mazur L. B. (2024c). “The Victim” and the democratization of victimhood. J. Theor. Philos. Psychol. 10.1037/teo0000290. [Epub ahead of print].

[B201] Mazur L. B. Richter L. Manz P. Bartels H. (2022). The importance of cultural psychological perspectives in pain research: towards the palliation of Cartesian anxiety. Theory Psychol. 32, 183–201. 10.1177/09593543211059124

[B202] Mazur L. B. Sticksel I. (2021). An empirical study of psychology and logic. Abduction and belief as normalizing habits of positive expectation. New Ideas Psychol. 63:100874. 10.1016/j.newideapsych.2021.100874

[B203] Mazur L. B. Watzlawik M. (2016). Debates about the scientific status of psychology: looking at the bright side. Integr. Psychol. Behav. Sci. 50, 555–567. 10.1007/s12124-016-9352-8 27318822

[B204] McGrane J. (2015). Stevens' forgotten crossroads: the divergent measurement traditions in the physical and psychological sciences from the mid-twentieth century. Front. Psychol. 6:431. 10.3389/fpsyg.2015.00431

[B205] McManus R. M Young L. Sweetman J. (2023). Psychology is a property of persons, not averages or distributions: confronting the group-to-person generalizability problem in experimental psychology. Adv. Methods Pract. Psychol. Sci. 6:1186615. 10.1177/25152459231186615

[B206] Mertens D. M. (2023). Research and Evaluation in Education and Psychology: Integrating Diversity with Quantitative, Qualitative, and Mixed Methods, 6th Edn. Thousand Oaks, CA: SAGE.

[B207] Messick S. (1989). “Validity,” in Educational Measurement, 3rd Edn., ed. Linn R. L. (New York: American Council on Education/Macmillan), 13–103.

[B208] Messick S. (1995). Validity of psychological assessment: validation of inferences from persons' responses and performances as scientific inquiry into score meaning. Am. Psychol. 50, 741–749. 10.1037/0003-066X.50.9.741

[B209] Michell J. (1990). An Introduction to the Logic of Psychological Measurement. Hillsdale, NJ: Lawrence Erlbaum Associates14523251

[B210] Michell J. (1997). Quantitative science and the definition of measurement in psychology. Br. J. Psychol. 88, 355–383. 10.1111/j.2044-8295.1997.tb02641.x

[B211] Michell J. (1999). Measurement in Psychology: Critical History of a Methodological Concept. Cambridge: Cambridge University Press.

[B212] Michell J. (2000). Normal science, pathological science and psychometrics. Theory Psychol. 10, 639–667. 10.1177/0959354300105004

[B213] Michell J. (2003). The quantitative imperative: positivism, naive realism and the place of qualitative methods in psychology. Theory Psychol. 13, 5–31. 10.1177/0959354303013001758

[B214] Michell J. (2012). Alfred Binet and the concept of heterogeneous orders. Front. Psychol. 3:261. 10.3389/fpsyg.2012.00261 22912619 PMC3419461

[B215] Michell J. (2014). The Rasch paradox, conjoint measurement, and psychometrics: response to Humphry and Sijtsma. Theory Psychol. 24, 111–123. 10.1177/0959354313517524

[B216] Michell J. (2020). Thorndike's Credo: metaphysics in psychometrics. Theory Psychol. 30, 309–328. 10.1177/0959354320916251

[B217] Michell J. (2022). “The art of imposing measurement upon the mind”: Sir Francis Galton and the genesis of the psychometric paradigm. Theory Psychol. 32, 375–400. 10.1177/09593543211017671

[B218] Michell J. (2023). “Professor Spearman has drawn over-hasty conclusions”: unravelling psychometrics' “Copernican Revolution”. Theory Psychol. 33, 661–680. 10.1177/09593543231179446

[B219] Michell J. Ernst C. (1996). The axioms of quantity and the theory of measurement, Part I. An English translation of holder (1901), Part I. J. Math. Psychol. 40, 235–252. 10.1006/jmps.1996.0023 8979975

[B220] Michell J. Ernst C. (1997). The axioms of quantity and the theory of measurement, Part II. An English translation of holder (1901), Part II. J. Math. Psychol. 41, 345–356. 10.1006/jmps.1997.1178 9473397

[B221] Miller A. (2024). “Realism”, in The Stanford Encyclopedia of Philosophy, eds. E. N. Zalta and U. Nodelman. Available online at: https://plato.stanford.edu/archives/sum2024/entries/realism (Accessed May 13, 2025).

[B222] Mislevy R. J. (2009). Validity from the perspective of model-based reasoning. CRESST Report 752. University of California, National Center for Research on Evaluation, Standards, and Student Testing (CRESST), Los Angeles, CA. Available online at: https://eric.ed.gov/?id=ED507085 (Accessed June 13, 2025).

[B223] Mislevy R. J. (2018). Sociocognitive Foundations of Educational Measurement. New York/London: Routledge.

[B224] Mislevy R. J. (2019). Advances in the science of measurement and cognition. Ann. Am. Acad. Politics Soc. Sci. 683, 164–182. 10.1177/0002716219843816

[B225] Mislevy R. J. (2024). Sociocognitive and argumentation perspectives on psychometric modeling in educational assessment. Psychometrika 89, 64–83. 10.1007/s11336-024-09966-5 38565794 PMC11063006

[B226] Molenaar P. C. M. (2004). A manifesto on psychology as idiographic science: bringing the person back into scientific psychology, this time forever. Measurement 2, 201–218. 10.1207/s15366359mea0204_1

[B227] Molenaar P. C. M. (2008). On the implications of the classical ergodic theorems: analysis of developmental processes has to focus on intra-individual variation. Dev. Psychobiol. 50, 60–69. 10.1002/dev.20262 18085558

[B228] Molenaar P. C. M. Campbell C. G. (2009). The new person-specific paradigm in psychology. Curr. Dir. Psychol. Sci. 18, 112–117. 10.1111/j.1467-8721.2009.01619.x

[B229] Montuori A. (2008). “Foreword,” in Transdisciplinarity. Theory and Practice, ed. B. Nicolescu (Cresskill, NJ: Hampton Press), IX–XVII.

[B230] Moore S. Speelman C. P. McGann M. (2023). Pervasiveness of effects in sample-based experimental psychology: a re-examination of replication data from nine famous psychology experiments. New Ideas Psychol. 68:100978. 10.1016/j.newideapsych.2022.100978

[B231] Muthukrishna M. Henrich J. (2019). A problem in theory. Nat. Hum. Behav. 3, 221–229. 10.1038/s41562-018-0522-1 30953018

[B232] Narens L. (2002). A meaningful justification for the representational theory of measurement. J. Math. Psychol. 46, 746–768. 10.1006/jmps.2002.1428

[B233] Newell D. Tiesinga E. (2019). The International System of Units (SI), 2019 Edition, Special Publication (NIST SP). Gaithersburg, MD: National Institute of Standards and Technology.

[B234] Newton P. E. Baird J. A. (2016). The great validity debate. Assess. Educ. Princ. Policy Pract. 23, 173–177. 10.1080/0969594X.2016.1172871

[B235] Nicolescu B. (2008). Transdisciplinarity – Theory and Practice. Cresskill, NJ: Hampton Press.

[B236] Nosek B. A. Alter G. Banks G. C. Borsboom D. Bowman S. D. Breckler S. J.. (2015). Promoting an open research culture: author guidelines for journals could help to promote transparency, openness, and reproducibility. Science 348, 1422–1425. 10.1126/science.aab2374 26113702 PMC4550299

[B237] Open Science Collaboration (2015). Estimating the reproducibility of psychological science. Science 349:aac4716. 10.1126/science.aac4716 26315443

[B238] Pace V. L. Brannick M. T. (2010). How similar are personality scales of the “same” construct? A meta-analytic investigation. Pers. Individ. Dif. 49, 669–676. 10.1016/j.paid.2010.06.014

[B239] Parsons C. (1990). The structuralist view of mathematical objects. Synthese 84, 303–346 10.1007/BF00485186

[B240] Peirce C. S. (1958). Collected Papers of Charles Sanders Peirce, Vols. 1-6, eds. C. Hartshorne and P. Weiss, Vols. 7-8, ed. A. W. Burks. Cambridge, MA: Harvard University Press.

[B241] Piaget J. (1972). “The epistemology of interdisciplinary relationships,” in Centre for Educational Research and Innovation (CERI). Interdisciplinarity: Problems of Teaching and Research in Universities (Paris, France: Organisation for Economic Co-operation and Development), 127–139.

[B242] Poli R. (2001). Foreword. Axiomathes 12, 1–5. 10.1023/A:1015841116773

[B243] Poli R. Seibt J. (Eds.) (2010). Theory and Applications of Ontology: Philosophical Perspectives. Dordrecht; Heidelberg; London; New York, NY: Springer Verlag.

[B244] Poovey M. (1998). A History of the Modern Fact: Problems of Knowledge in the Sciences of Wealth and Society. Chicago: Chicago University Press.

[B245] Porter T. (1995). Trust in Numbers. The Pursuit of Objectivity in Science and Public Life. Princeton, N.J: Princeton University Press.

[B246] Ramage M. Shipp K. (2020). Systems Thinkers (2nd Edn.). London, UK: Springer.

[B247] Rasch G. (1960). Probabilistic Models for Some Intelligence and Attainment Tests. Studies Im Mathematical Psychology. Kopenhagen: Danmarks pædagogiske Institut.

[B248] Resnick M. D. (1997). Mathematics as a Science of Patterns. Oxford, England: Clarendon.

[B249] Revelle W. (2024). The seductive beauty of latent variable models: or why I don't believe in the Easter Bunny. Pers. Indiv. Diff. 221, 112552, 1–17. 10.1016/j.paid.2024.112552

[B250] Richters J. E. (2021). Incredible utility: the lost causes and causal debris of psychological science. Basic Appl. Soc. Psych. 43, 366–405. 10.1080/01973533.2021.1979003

[B251] Robinson O. C. (2011). The idiographic/nomothetic dichotomy: tracing historical origins of contemporary confusions. History Philos. Psychol. 13, 32–39. 10.53841/bpshpp.2011.13.2.32

[B252] Rogoff B. (2011). Developing Destinies: A Mayan Midwife and Town. Oxford: Oxford University Press.

[B253] Rose T. (2016). The End of Average: How to Succeed in a World that Values Sameness. New York: Harper Collins.

[B254] Rosen R. (1985). Anticipatory Systems: Philosophical, Mathematical, and Methodological Foundations. New York: Elsevier Science and Technology Books.

[B255] Rosen R. (1991). Life Itself. A Comprehensive Inquiry into the Nature, Origin, and Fabrication of Life. New York: Columbia University Press.

[B256] Rosen R. (1999). Essays on Life Itself . New York, NY: Columbia University Press.

[B257] Rudmin F. Trimpop R. M. Kryl I. Boski P. (1987). Gustav Ichhieser in the history of social psychology: an early phenomenology of social attribution. Br. J. Soc. Psychol. 26, 165–180. 10.1111/j.2044-8309.1987.tb00777.x

[B258] Rudolph L. (2013). “Qualitative mathematics for the social sciences,” in Mathematical Models for Research on Cultural Dynamics, ed. L. Rudolph (London: Routledge), 492.

[B259] Salvatore S. Valsiner J. (2010). Between the general and the unique: overcoming the nomothetic versus idiographic opposition. Theory Psychol. 20, 817–833. 10.1177/0959354310381156

[B260] Sato T. Hidaka T. Fukuda M. (2009). “Depicting the dynamics of living the life: the trajectory equifinality model,” in Dynamic Process Methodology in the Ssocial and Developmental Sciences, eds. J. Valsiner, P. C. M. Molenaar, M. C. D. P. Lyra, and N. Chaudhary (New York, NY: Springer US), 217–240.

[B261] Schimmack U. (2021). The validation crisis in psychology. Meta-Psychology 5:1645. 10.15626/MP.2019.1645

[B262] Schönemann P. H. (1994). “Measurement: the reasonable ineffectiveness of mathematics in the social sciences,” in Trends and Perspectives in Empirical Social Research, eds. I. Borg and P. P. Mohler (Berlin; New York, NY: De Gruyter), 149–160.

[B263] Schrödinger E. (1964). What is Life? Reprinted in: What is life? With Mind and Matter and Autobiographical Sketches. Cambridge, UK: Cambridge University Press.

[B264] Schwager K. W. (1991). The representational theory of measurement: an assessment. Psychol. Bull. 110, 618–626. 10.1037/0033-2909.110.3.618

[B265] Sechrest L. McKnight P. McKnight K. (1996). Calibration of measures for psychotherapy outcome studies. Am. Psychol. 51, 1065–1071. 10.1037/0003-066X.51.10.1065 8870543

[B266] Semin G. (1989). The contribution of linguistic factors to attribute inference and semantic similarity judgements. Eur. J. Soc. Psychol. 19, 85–100. 10.1002/ejsp.2420190202

[B267] Serva M. A. Fuller M. A. Mayer R. C. (2005). The reciprocal nature of trust: a longitudinal study of interacting teams. J. Organ. Behav. 26, 625–648. 10.1002/job.331

[B268] Sherry D. (2011). Thermometers and the foundations of measurement. Philos. Sci. 78, 512–528.

[B269] Shweder R. A. (1977). Likeness and likelihood in everyday thought: magical thinking in judgments about personality. Curr. Anthropol. 18, 637–658. 10.1086/201974

[B270] Shweder R. A. D'Andrade R. G. (1980). “The systematic distortion hypothesis,” in Fallible Judgment in Behavioral Research: New Directions for Methodology of Social and Behavioral Science, ed. R. A. Shweder (San Francisco, CA: Jossey-Bass), 37–58.

[B271] Simmel G. (1900/1978). The Philosophy of Money (T. Bottomore and D. Frisby, Trans.). London: Routledge.

[B272] Skinner E. A. (1996). A guide to constructs of control. J. Pers. Soc. Psychol. 71, 549–570. 10.1037/0022-3514.71.3.549 8831161

[B273] Slaney K. L. (2017). “Some conceptual housecleaning,” in Validating Psychological Constructs: Historical, Philosophical, and Practical Dimensions (London: Palgrave Macmillan), 201–234.

[B274] Smedslund G. Arnulf J. K. Smedslund J. (2022). Is psychological science progressing? Explained variance in PsycINFO articles during the period 1956 to 2022. Front. Psychol. 13:1089089. 10.3389/fpsyg.2022.1089089 36619094 PMC9810988

[B275] Smedslund J. (1978). Bandura's theory of self-efficacy: a set of common sense theorems. Scand. J. Psychol. 19, 1–14. 10.1111/j.1467-9450.1978.tb00299.x

[B276] Smedslund J. (1988). Psycho-Logic. Berlin, Heidelberg: Springer.

[B277] Smedslund J. (1991). The pseudoempirical in psychology and the case for psychologic. Psychol. Inq. 2, 325–338. 10.1207/s15327965pli0204_1

[B278] Smedslund J. (2012). The bricoleur model of psychological practice. Theory Psychol. 22, 643–657. 10.1177/095935431244127715892727

[B279] Smedslund J. (2016). Why psychology cannot be an empirical science. Integr. Psychol. Behav. Sci. 50, 185–195. 10.1007/s12124-015-9339-x 26712604

[B280] Smedslund J. (2021). From statistics to trust: psychology in transition. New Ideas Psychol. 61:100848. 10.1016/j.newideapsych.2020.100848

[B281] Spearman C. (1904). General intelligence, objectively determined and measured. Am. J. Psychol. 15, 201–293. 10.2307/141210738532229

[B282] Speelman C. P. McGann M. (2013). How mean is the mean? Front. Psychol. 4:451. 10.3389/fpsyg.2013.00451 23888147 PMC3719041

[B283] Speelman C. P. McGann M. (2020). Statements about the pervasiveness of behaviour require data about the pervasiveness of behaviour. Front. Psychol. 11:594675. 10.3389/fpsyg.2020.594675 33329258 PMC7711086

[B284] Speelman C. P. Parker L. Rapley B. J. McGann M. (2024). Most psychological researchers assume their samples are ergodic: evidence from a year of articles in three major journals. Collabra Psychol. 10:92888. 10.1525/collabra.92888

[B285] Sperber D. (1996). Explaining Culture: A Naturalistic Approach. Oxford: Blackwell.

[B286] Steinmetz G. (ed.). (2005). The Politics of Method in the Human Sciences: Positivism and its Epistemological Others. Durham, NC: Duke University Press.

[B287] Stern W. (1911). Die Differentielle Psychologie in Ihren Methodischen Grundlagen [Differential Psychology in its Methodological Foundations]. Leipzig: Barth.

[B288] Stevens S. S. (1935). The operational definition of psychological concepts. Psychol. Rev. 42, 517–527. 10.1037/h0056973

[B289] Stevens S. S. (1946). On the theory of scales of measurement. Science 103, 677–680. 10.1126/science.103.2684.677 17750512

[B290] Stevens S. S. (1958). Measurement and man. Sci. New Series 127, 383–389. 10.1126/science.127.3295.383 13506588

[B291] Storozuk A. Ashley M. Delage V. Maloney E. A. (2020). Got bots? Practical recommendations to protect online survey data from bot attacks. Quant. Methods Psychol. 16, 472–481. 10.20982/tqmp.16.5.p472

[B292] Straub D. W. Boudreau M.-C. Gefen D. (2004). Validation guidelines for IS positivist research. Commun. Assoc. Inf. Syst. 13, 380–427. 10.17705/1CAIS.0132433241613

[B293] Sugarman J. (2017). Psychologism as a style of reasoning and the study of persons. New Ideas Psychol. 44, 21–27. 10.1016/j.newideapsych.2016.11.008

[B294] Sugarman J. Martin J. (2020). A Humanities Approach to the Psychology of Personhood. London: Routledge.

[B295] Suppes P. Krantz D. Luce D. Tversky A. (1989). Foundations of Measurement, Vol. II: Geometrical, Threshold, and Probabilistic Representations. New York, NY: Academic Press.

[B296] Suppes P. Zinnes J. (1963). “Basic measurement theory,” in Handbook of Mathematical Psychology, ed. D. Luce (New York, NY: John Wiley and Sons), 1–76.

[B297] Tal E. (2020). “Measurement in science,” in The Stanford Encyclopedia of Philosophy (Fall 2020). Metaphysics Research Lab, Stanford University, ed. Edward N. Zalta. Available online at: https://plato.stanford.edu/archives/fall2020/entries/measurement-science (Accessed October 21, 2023).

[B298] Tang R. Braver T. S. (2020). Towards an individual differences perspective in mindfulness training research: theoretical and empirical considerations. Front. Psychol. 11:818. 10.3389/fpsyg.2020.00818 32508702 PMC7248295

[B299] Taylor C. (1985). “Peaceful coexistence in psychology,” in Human Agency and Language, ed. C. Taylor (New York: Cambridge University Press), 117–138.

[B300] Thomas M. A. (2019). Mathematization, not measurement: a critique of Stevens' scales of measurement. J. Methods Meas. Soc. Sci. 10, 76–94. 10.2458/v10i2.23785

[B301] Thorndike E. L. (1903). Notes on Child Study (2nd Edn.). New York, NY: Macmillan.

[B302] Thurstone L. L. (1928). Attitudes can be measured. Am. J. Sociol. 33, 529–554. 10.1086/214483

[B303] Tolman C. W. (ed.). (1992). Positivism in Psychology: Historical and Contemporary Problems. New York, NY: Springer.

[B304] Toomela A. (2000). Activity theory is a dead end for cultural-historical psychology. Cult. Psychol. 6, 353–364. 10.1177/1354067X0063005

[B305] Toomela A. (2003). “Development of symbol meaning and the emergence of the semiotically mediated mind,” in Cultural Guidance in the Development of the Human Mind, ed. A. Toomela (Westport, CT: Ablex Publishing), 163–209.

[B306] Toomela A. (2007a). Culture of science: strange history of the methodological thinking in psychology. Integr. Psychol. Behav. Sci. 41, 6–20. 10.1007/s12124-007-9004-0 17992864

[B307] Toomela A. (2007b). Sometimes one is more than two: when collaboration inhibits knowledge construction. Integr. Psychol. Behav. Sci. 41, 198–207. 10.1007/s12124-007-9015-x 18193521

[B308] Toomela A. (2007c). “Unifying psychology: absolutely necessary, not only useful,” in Psicologia: Novas direcoes no dialogo com outros campos de saber, eds. A. V. B. Bastos and N. M. D. Rocha (São Paulo: Casa do Psicologo), 449–464.

[B309] Toomela A. (2008a). Activity theory is a dead end for methodological thinking in cultural psychology too. Cult. Psychol. 14, 289–303. 10.1177/1354067X08088558

[B310] Toomela A. (2008b). Variables in psychology: a critique of quantitative psychology. Integr. Psychol. Behav. Sci. 42, 245–265. 10.1007/s12124-008-9059-6 18528738

[B311] Toomela A. (2008c). Vygotskian cultural-historical and sociocultural approaches represent two levels of analysis: complementarity instead of opposition. Culture and Psychology 14, 57–69. 10.1177/1354067X07085812

[B312] Toomela A. (2009a). “How methodology became a toolbox - and how it escapes from that box,” in Dynamic Process Methodology in the Social and Developmental Sciences, eds. J. Valsiner, P. Molenaar, M. Lyra, and N. Chaudhary (New York: Springer), 45–66.

[B313] Toomela A. (2009b). “Kurt Lewin's contribution to the methodology of psychology: from past to future skipping the present,” in The Observation of Human Systems. Lessons from the History of Anti-Reductionistic Empirical Psychology, ed. J. Clegg (New Brunswick, NJ: Transaction Publishers), 101–116.

[B314] Toomela A. (2010). “Modern mainstream psychology is the best? Noncumulative, historically blind, fragmented, atheoretical,” in Methodological Thinking in Psychology: 60 Years Gone Astray?, eds. A. Toomela and J. Valsiner (Charlotte: Information Age Publishers), 1–26.

[B315] Toomela A. (2011). Travel into a fairy land: a critique of modern qualitative and mixed methods psychologies. Integr. Psychol. Behav. Sci. 45, 21–47. 10.1007/s12124-010-9152-5 21258882

[B316] Toomela A. (2012). “Guesses on the future of cultural psychology: past, present, and past,” in The Oxford Handbook of Culture and Psychology, ed. J. Valsiner (New York: Oxford University Press), 998–1033.

[B317] Toomela A. (2014a). “Mainstream psychology,” in Encyclopedia of Critical Psychology, ed. T. Teo (New York: Springer), 1117–1125.

[B318] Toomela A. (2014b). Methodology of cultural-historical psychology. In A. Yasnitsky, R. van der Veer, and M. Ferrari (Eds.), The Cambridge Handbook of Cultural-Historical Psychology (pp. 99-125). Cambridge: Cambridge University Press. 10.1017/CBO9781139028097.007

[B319] Toomela A. (2014c). “Modern qualitative approach to psychology: art or science?,” in Multicentric Identities in a Globalizing World, eds. S. Salvatore, A. Gennaro, and J. Valsiner (Charlotte, NC: Information Age Publishing), 75–82.

[B320] Toomela A. (2014d). “A structural systemic theory of causality and catalysis,” in The Catalyzing Mind. Beyond Models of Causality, eds. K. R. Cabell and J. Valsiner (New York: Springer), 271–292.

[B321] Toomela A. (2015). Vygotsky's theory on the Procrustes' bed of linear thinking: looking for structural-systemic theseus to save the idea of ‘social formation of mind'. Cult. Psychol. 21, 318–339. 10.1177/1354067X15570490

[B322] Toomela A. (2016a). “The ways of scientific anticipation: from guesses to probabilities and from there to certainty,” in Anticipation Across Disciplines, ed. M. Nadin (Cham: Springer. 255–273.

[B323] Toomela A. (2016b). What are higher psychological functions? Integr. Psychol. Behav. Sci. 50, 91–121. 10.1007/s12124-015-9328-0 26403987

[B324] Toomela A. (2017). “Towards general-unifying theory of psychology: Engelsted and beyond,” in Catching up with Aristotle. A Journey in Quest for General Psychology, ed. N. Engelsted (Cham: Springer), 137–150.

[B325] Toomela A. (2019). The Psychology of Scientific Inquiry. Cham: Springer Nature.

[B326] Toomela A. (2020). Culture, Speech and My Self. Sepamäe: Porcos ante Margaritas.

[B327] Toomela A. (2022). “Methodology of science: different kinds of questions require different methods,” in Experimental Psychology: Ambitions and Possibilities, eds. D. Gozli and J. Valsiner (Chum: Springer), 113–151.

[B328] Toomela A. Valisiner J. (2010). Methodological thinking in psychology: 60 years gone astray? US: IAP.

[B329] Torgerson W. S. (1958). Theory and Methods of Scaling. New York, NY: Wiley.

[B330] Trendler G. (2009). Measurement theory, psychology and the revolution that cannot happen. Theor. Psychol. 19, 579–599. 10.1177/0959354309341926

[B331] Trendler G. (2013). Measurement in psychology: a case of ignoramus et ignorabimus? A rejoinder. Theor. Psychol. 23, 591–615. 10.1177/0959354313490451

[B332] Trendler G. (2019a). Conjoint measurement undone. Theor. Psychol. 29, 100–128. 10.1177/0959354318788729

[B333] Trendler G. (2019b). Measurability, systematic error, and the replication crisis: a reply to Michell (2019) and Krantz and Wallsten (2019). Theor. Psychol. 29, 144–151. 10.1177/0959354318824414

[B334] Trendler G. (2022a). Is measurement in psychology an empirical or a conceptual issue? A comment on David Franz. Theor. Psychol. 32, 164–170. 10.1177/09593543211050025

[B335] Trendler G. (2022b). The incoherence of Rasch measurement: a critical comparison between measurement in psychology and physics. Pers. Individ. Dif. 189:111408. 10.1016/j.paid.2021.111408

[B336] Uher J. (2011). Individual behavioral phenotypes: an integrative meta-theoretical framework. Why ‘behavioral syndromes' are not analogues of ‘personality'. Dev. Psychobiol. 53, 521–548. 10.1002/dev.20544 21432848

[B337] Uher J. (2013). Personality psychology: lexical approaches, assessment methods, and trait concepts reveal only half of the story-Why it is time for a paradigm shift. Integr. Psychol. Behav. Sci. 47, 1–55. 10.1007/s12124-013-9230-6 23389471 PMC3581768

[B338] Uher J. (2015a). Conceiving “personality”: psychologist's challenges and basic fundamentals of the transdisciplinary philosophy-of-science paradigm for research on individuals. Integr. Psychol. Behav. Sci. 49, 398–458. 10.1007/s12124-014-9283-1 25281293

[B339] Uher J. (2015b). Developing “personality” taxonomies: metatheoretical and methodological rationales underlying selection approaches, methods of data generation and reduction principles. Integr. Psychol. Behav. Sci. 49, 531–589. 10.1007/s12124-014-9280-4 25249469

[B340] Uher J. (2015c). Interpreting “personality” taxonomies: why previous models cannot capture individual-specific experiencing, behaviour, functioning and development. Major taxonomic tasks still lay ahead. Integr. Psychol. Behav. Sci. 49, 600–655. 10.1007/s12124-014-9281-3 25311311

[B341] Uher J. (2015d). “Agency enabled by the psyche: explorations using the transdisciplinary philosophy-of-science paradigm for research on individuals,” in Constraints of Agency: Explorations of Theory in Everyday Life. Annals of Theoretical Psychology, Vol. 12, eds. C. W. Gruber, M. G. Clark, S. H. Klempe, and J. Valsiner (New York: Springer International Publishing), 177–228.

[B342] Uher J. (2016a). “Exploring the workings of the Psyche: metatheoretical and methodological foundations,” in Psychology as the Science of Human Being: The Yokohama Manifesto, eds. Valsiner J. Marsico G. Chaudhary N. Sato T. Dazzani V. (New York: Springer International Publishing), 299–324.

[B343] Uher J. (2016b). What is behaviour? And (when) is language behaviour? A metatheoretical definition. J. Theory Soc. Behav. 46, 475–501. 10.1111/jtsb.12104

[B344] Uher J. (2018a). Quantitative data from rating scales: an epistemological and methodological enquiry. Front. Psychol. 9:2599. 10.3389/fpsyg.2018.02599 30622493 PMC6308206

[B345] Uher J. (2018b). Taxonomic models of individual differences: a guide to transdisciplinary approaches. Philos. Trans. Royal Soc. B 373:20170171. 10.1098/rstb.2017.0171 29483354 PMC5832694

[B346] Uher J. (2018c). “The transdisciplinary philosophy-of-science paradigm for research on individuals: foundations for the science of personality and individual differences,” in The SAGE Handbook of Personality and Individual Differences: Volume I: The science of Personality and Individual Differences, eds. Zeigler-Hill V. Shackelford T. K. (London, UK: SAGE), 84–109.

[B347] Uher J. (2019). Data generation methods across the empirical sciences: differences in the study phenomena's accessibility and the processes of data encoding. Qual. Quan. Int. J. Methodol. 53, 221–246. 10.1007/s11135-018-0744-3

[B348] Uher J. (2020a). Measurement in metrology, psychology and social sciences: data generation traceability and numerical traceability as basic methodological principles applicable across sciences. Qual. Quan. 54, 975–1004. 10.1007/s11135-020-00970-2

[B349] Uher J. (2020b). Human uniqueness explored from the uniquely human perspective: epistemological and methodological challenges. J. Theory Soc. Behav. 50, 20–24. 10.1111/jtsb.12232

[B350] Uher J. (2021a). Psychometrics is not measurement: unraveling a fundamental misconception in quantitative psychology and the complex network of its underlying fallacies. J. Theor. Philos. Psychol. 41:58. 10.1037/teo0000176

[B351] Uher J. (2021b). Quantitative psychology under scrutiny: measurement requires not result-dependent but traceable data generation. Pers. Individ. Dif. 170:110205. 10.1016/j.paid.2020.110205

[B352] Uher J. (2021c). Psychology's status as a science: peculiarities and intrinsic challenges. Moving beyond its current deadlock towards conceptual integration. Integr. Psychol. Behav. Sci. 55, 212–224. 10.1007/s12124-020-09545-0 32557115 PMC7801307

[B353] Uher J. (2021d). Problematic research practices in psychology: misconceptions about data collection entail serious fallacies in data analyses. Theor. Psychol. 31, 411–416. 10.1177/09593543211014963

[B354] Uher J. (2022a). Functions of units, scales and quantitative data: fundamental differences in numerical traceability between sciences. Qual. Quan. 56, 2519–2548. 10.1007/s11135-021-01215-6

[B355] Uher J. (2022b). Rating scales institutionalise a network of logical errors and conceptual problems in research practices: a rigorous analysis showing ways to tackle psychology's crises. Front. Psychol. 13:1009893. 10.3389/fpsyg.2022.1009893 36643697 PMC9833395

[B356] Uher J. (2023a). What's wrong with rating scales? Psychology's replication and confidence crisis cannot be solved without transparency in data generation. Soc. Personal. Psychol. Compass 17:e12740. 10.1111/spc3.12740

[B357] Uher J. (2023b). What are constructs? Ontological nature, epistemological challenges, theoretical foundations and key sources of misunderstandings and confusions. Psychol. Inq. 34, 280–290. 10.1080/1047840X.2023.2274384

[B358] Uher J. (2024). “Transdisciplinarity, complexity thinking and dialectics,” in The Routledge International Handbook of Dialectical Thinking, eds. Shannon N. Mascolo M. Belolutskaya A. (London: Routledge), 259–277. 10.4324/9781003317340-21

[B359] Uher J. (2025). Statistics is not measurement: the inbuilt semantics of psychometric scales and language-based models obscures crucial epistemic differences. Front. Psychol. 16:1534270. 10.3389/fpsyg.2025.1534270 40642039 PMC12244230

[B360] Valsiner J. (1998). The Guide Mind. A Sociogenetic Approach to Personality. Cambridge, MA: Harvard University Press.

[B361] Valsiner J. (2000). Culture and Human Development. London: Sage.

[B362] Valsiner J. (2007). Culture in Minds and Societies. Foundations of Cultural Psychology. Thousand Oaks, CA: Sage.

[B363] Valsiner J. (2012). A Guided Science: History of Psychology in the Mirror of Its Making. New Brunswick, NJ: Transaction Publishers.

[B364] Valsiner J. (2014a). An Invitation to Cultural Psychology. London: SAGE Publications.

[B365] Valsiner J. (2014b). Needed for cultural psychology: methodology in a new key. Cult. Psychol. 20, 3–30. 10.1177/1354067X13515941

[B366] Valsiner J. (2017). From Methodology to Methods in Human Psychology. Cham: Springer.

[B367] Valsiner J. Brinkmann S. (2016). “Beyond the “variables”: developing metalanguage for psychology,” in Centrality of History for Theory Construction in Psychology, Annals of Theoretical Psychology, eds. Klempe S. Smith R. (New York: Springer), 75–90.

[B368] van Fraassen B. C. (2008). Scientific Representation: Paradoxes of Perspective. New York: Oxford University Press.

[B369] van Geert P. (2011). The contribution of complex dynamic systems to development. Child Dev. Perspect. 5, 273–278. 10.1111/j.1750-8606.2011.00197.x

[B370] van Inwagen P. Sullivan M. Bernstein S. (2023). “Metaphysics,” in The Stanford Encyclopedia of Philosophy (Summer 2023 Edition), eds. Zalta E. N. Nodelman U.. Available online at: https://plato.stanford.edu/archives/sum2023/entries/metaphysics (Accessed May 18, 2025).

[B371] Velleman P. F. Wilkinson L. (1993). Nominal ordinal interval and ratio typologies are misleading. Am. Stat. 47, 65–72. 10.1080/00031305.1993.10475938

[B372] Vessonen E. (2017). Psychometrics versus representational theory of measurement. Philos. Soc. Sci. 47, 330–350. 10.1177/0048393117705299

[B373] von Eye A. Bergman L. R. (2003). Research strategies in developmental psychopathology: dimensional identity and the person-oriented approach. Dev. Psychopathol. 15, 553–580. 10.1017/S0954579403000294 14582932

[B374] von Eye A. Bogat G. A. (2006). Person-oriented and variable-oriented research: concepts, results, and development. Merrill-Palmer Q. 52, 390–420. 10.1353/mpq.2006.003234409987

[B375] von Helmholtz H. (1887). “Zählen und Messen, erkenntnisstheoretisch betrachtet,” in Philosophische Aufsätze, Eduard Zeller zu seinem fünfzigjährigen Doctorjubiläum gewidmet, ed. von Vischer F. T. (Leipzig: Fues' Verlag), 17–52.

[B376] von Kries J. (1882). Ueber die Messung intensiver Grössen und über das sogenannte psychophysische Gesetz. Viertelj. Wiss. Philos. 6, 256–294.

[B377] von Neumann J. (1955). Mathematical foundations of quantum mechanics [original: Mathematische Grundlagen der Quantenmechanik in 1935]. Princeton, NJ: Princeton University Press.

[B378] Vygotsky L. S. (1962). Thought and Language. Cambridge, MA: MIT Press.

[B379] Vygotsky L. S. (1982). “Istoricheski smysl psikhologicheskogo krizisa. Metodologicheskoje issledovanije. (Historical meaning of the crisis in psychology. A methodological study. Originally written in 1927; First published in 1982,” in L. S. Vygotsky. Sobranije sochinenii. Tom 1. Voprosy teorii i istorii psikhologii, eds. Luria A. R. Jaroshevskii M. G. (Moscow: Pedagogika), 291–436.

[B380] Vygotsky L. S. (1994). “The problem of the cultural development of the child (Originally published in 1929),” in The Vygotsky Reader, eds. Veer R. v. d. Valsiner J. (Oxford: Blackwell), 57–72.

[B381] Vygotsky L. S. (1997). “The historical meaning of the crisis in psychology: a methodological investigation,” in The Collected Works of L. S. Vygotsky. Volume 3. Problems of the Theory and History of Psychology, eds. Rieber R. W. Wollock J. (New York: Springer), 233–343.

[B382] Weber M. (1904–05/1992). The Protestant Ethic and the Spirit of Capitalism (T. Parsons, Trans.). New York: Routledge.

[B383] Weber M. (1949). The Methodology of the Social Sciences [Translated and edited by E.A. Shils and H.A. Finch]. New York: Free Press.

[B384] Weber R. (2012). Evaluating and developing theories in the information systems discipline. J. Assoc. Inf. Syst. 13, 1–30. 10.17705/1jais.00284

[B385] Weber R. (2021). Constructs and indicators: an ontological analysis. MIS Q. 45, 1644–1678. 10.25300/MISQ/2021/15999

[B386] Werner H. (1948). Comparative Psychology of Mental Development. New York: International Universities Press.

[B387] White R. (2011). The meaning of measurement in metrology. Accred. Qual. Assur. 16, 31–41. 10.1007/s00769-010-0698-1

[B388] Wierzbicka A. (1996). Semantics: Primes and Universals. New York, NY: Oxford University Press.

[B389] Williams R. N. (2015). “Introduction,” in Scientism: The New Orthodoxy, eds. Williams R. N. Robinson D. N. (New York, NY: Bloomsbury Academic), 1–21.

[B390] Windelband W. (1904/1998). History and Natural Science. Theor. Psychol. 8, 5–22. 10.1177/0959354398081001

[B391] Wittgenstein L. (1953). Philosophical Investigations (G. E. M. Anscombe Trans.). New York, NY: Wiley-Blackwell.

[B392] Wundt W. (1897). Outlines of Psychology. Leipzig: Wilhelm Engelman.

[B393] Yarkoni T. (2022). The generalizability crisis. Behav. Brain Sci. 45:e1. 10.1017/S0140525X20001685 33342451 PMC10681374

[B394] Zell E. Krizan Z. Teeter S. R. (2015). Evaluating gender similarities and differences using metasynthesis. Am. Psychol. 70, 10–20. 10.1037/a0038208 25581005

[B395] Zwaan R. A. Etz A. Lucas R. E. Donnellan M. B. (2017). Making replication mainstream. Behav. Brain Sci. 1–50. 10.31234/osf.io/4tg9c 29065933

[B396] Zwaan R. A. Pecher D. Paolacci G. Bouwmeester S. Verkoeijen P. Dijkstra K.. (2018). Participant nonnaiveté and the reproducibility of cognitive psychology. Psychon. Bull. Rev. 25, 1968–1972. 10.3758/s13423-017-1348-y 28744765

